# Synthesis of robust underwater glues from common proteins via unfolding-aggregating strategy

**DOI:** 10.1038/s41467-023-40856-z

**Published:** 2023-08-24

**Authors:** Yongchun Liu, Ke Li, Juanhua Tian, Aiting Gao, Lihua Tian, Hao Su, Shuting Miao, Fei Tao, Hao Ren, Qingmin Yang, Jing Cao, Peng Yang

**Affiliations:** 1 Key Laboratory of Applied Surface and Colloid Chemistry, Ministry of Education, School of Chemistry and Chemical Engineering, Shaanxi Normal University, Xi’an, 710119 China; 2 Xi’an Key Laboratory for Prevention and Treatment of Common Aging Diseases, Translational and Research Centre for Prevention and Therapy of Chronic Disease, Institute of Basic and Translational Medicine, Xi’an Medical University, Xi’an, 710021 China; 3 Department of Urology, The Second Affiliated Hospital of Xi’an Jiaotong University, Xi’an, 710004 China; 4 Key Laboratory of Archaeological Exploration and Cultural Heritage Conservation Technology, Ministry of Education, Institute of Culture and Heritage, Northwestern Polytechnical University, Xi’an, 710072 China

**Keywords:** Biomaterials - proteins, Gels and hydrogels, Gels and hydrogels

## Abstract

Underwater adhesive proteins secreted by organisms greatly inspires the development of underwater glue. However, except for specific proteins such as mussel adhesive protein, barnacle cement proteins, curli protein and their related recombinant proteins, it is believed that abundant common proteins cannot be converted into underwater glue. Here, we demonstrate that unfolded common proteins exhibit high affinity to surfaces and strong internal cohesion via amyloid-like aggregation in water. Using bovine serum albumin (BSA) as a model protein, we obtain a stable unfolded protein by cleaving the disulfide bonds and maintaining the unfolded state by means of stabilizing agents such as trifluoroethanol (TFE) and urea. The diffusion of stabilizing agents into water exposes the hydrophobic residues of an unfolded protein and initiates aggregation of the unfolded protein into a solid block. A robust and stable underwater glue can thus be prepared from tens of common proteins. This strategy deciphers a general code in common proteins to construct robust underwater glue from abundant biomass.

## Abstract (web summary)

Underwater adhesive proteins greatly inspired the development of underwater glue but except for specific proteins it is believed that abundant common proteins cannot be converted into underwater glue. Here, the authors demonstrate, using bovine serum albumin as model protein, that unfolded common proteins exhibit high affinity to surfaces and strong internal cohesion via amyloid-like aggregation in water.

## Introduction

Robust and stable underwater glues have important applications in industry and biomedical field, as well as daily life^1–4^. However, synthetic adhesives developed for dry applications often perform poorly in high-humidity conditions because they tend to interact with water rather than a material surface. Even if some adhesive systems are able to penetrate the hydrated layers on the solid surface, the bulk of the solidified glue will gradually disintegrate as a result of the hydration and decomposition from water during long-term application^5,6^. In contrast, powerful underwater adhesion in nature occurs ubiquitously from microscopic cell adhesion^7^ to macroscopic adhesion of marine organisms fixed to an external host^8^. With inspiration from these organisms, a series of outstanding underwater adhesion systems have been carefully orchestrated through a fascinating blend of catechol groups^9,10^, polyelectrolyte assemblies^11,12^, and supramolecular architectures^4,13^. Most systems have disadvantages such as high cost, low atom economy, tedious procedures, oxidization of catechol groups, and swelling that leads to a decrease in adhesion^9,14^. More importantly, few protein-based adhesives have an adhesion strength and stability in water comparable to those of natural bioglues.

Barnacle cement, one of the few protein-based adhesives that exhibit strong underwater adhesion^15^, can reach a strength on the megapascal level^16^. The strength and stability of this adhesive are beyond the reach of most synthetic glues. Extensive studies have demonstrated that the basic mechanisms underlying the adhesion of barnacle cement are related to the *β*-sheet-rich assembly of the protein, which is called an amyloid structure^17,18^. Thus, the amyloid structure is considered to have enormous potential in the development of robust and stable underwater glues. This conclusion is also supported by the fact that the amyloid structure is recognized to be involved in the adhesion of biofilms of gram-negative bacteria such as *Escherichia coli* and *Salmonella*^7^. However, except for some amyloid structures of particular adhesion-related proteins found in barnacle cement and bacteria, most classic amyloid assemblies, such as traditional amyloid fibers assembled by common proteins^19^, do not exhibit underwater adhesion ability. For example, the adsorbability of lysozyme amyloid fibrils on bare Au sheets is only 65 ng/cm^2^, which is even much lower than that of native lysozyme (~2000 ng/cm^2^)^20^. Therefore, we urgently need to elucidate the mechanism underlying amyloid-mediated bioadhesion and thereby design a straightforward strategy to transform common commercial proteins into robust underwater glue. However, there are two greatest challenge in transferring common protein to a strong and robust underwater glue: (i) how to break through the hydration layer to allow protein to come into contact with the substrate; (ii) how to make these water-soluble proteins produce strong cohesive forces to resist the dissolving and swelling effects of water.

In this work, we discover that the mechanism of amyloid-mediated bioadhesion includes conformation-directed adhesion enhancement to transform common proteins into reliable underwater glue. Based on this principle, a simple unfolding-aggregating strategy is developed to afford simultaneously high affinity to surfaces and strong internal cohesion for a protein-based underwater glue. Specifically, common proteins are unfolded by cleavage of their native disulfide bonds and stabilized into a single-molecular unfolded state by interacting with a stabilizer. In comparison with native proteins, the stabilized unfolding protein chains have more functional groups exposed outwards, with dramatically enhanced chain freedom. As a result, once the stabilizer is removed by quick exchange with water, unfolded proteins with flexible chain freedom can fully interact with a surface through exposed functional groups along protein chains. In such a case, the intermolecular interactions among protein chains that lead to strong internal cohesion via the formation of amyloid structures as well as the interactions between protein chains and surfaces to promote interfacial adhesion are highly strengthened. By this rule, tens of common proteins and protein-containing biological fluids could be transformed into robust underwater glues with unprecedented high adhesion strength (~20 times stronger than mussel byssal threads) and stability (at least 2 years of underwater immersion). This system may significantly lower the fabrication threshold of underwater glue owing to its low cost, high atom economy, simple procedure as well as strong and stable underwater adhesion. This finding highlights the role of protein conformation control in the design of underwater bioglues, which has not been fully recognized to date.

## Results

### The principle of unfolding-aggregation-based underwater adhesive

**Fig. 1:**
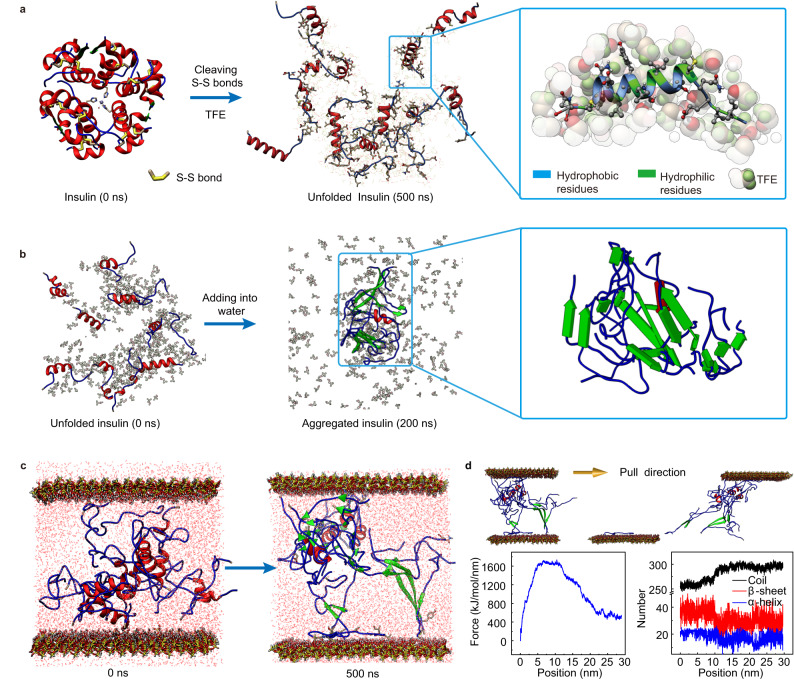
The unfolding and aggregation process of insulin and its adhesive behavior based on MD simulation. **a** The unfolding of insulin in a TFE aqueous solution (80%) after cleavage of the disulfide bonds. **b** Aggregation of the unfolding insulin chains in water with TFE on their surface. **c** The adhesive behavior of unfolded insulin between two layers of silica. **d** Snapshot, pull force curve and second structural development of unfolded insulin in the steered MD simulation. Silica bonded by the unfolded insulin chain was subjected to a pull force in the direction parallel to the silica sheets.

Adhesive-related proteins are usually unstructured to provide more flexible molecular chains that can aggregate into solidified blocks with adhesive ability^21–23^. Therefore, it has been speculated that the critical procedure to transform common proteins into underwater glue is protein unfolding. Generally, the physiological structure of proteins is stabilized by intramolecular disulfide bonds and some noncovalent interactions including electrostatic, hydrophobic, and hydrogen bonding interactions. Thus, to obtain unfolded proteins, the disulfide bonds were cleaved^24^, and the stabilizer, such as trifluoroethanol (TFE) or urea, which surrounds the protein chains more favorably than water, was used to shield noncovalent interactions among the protein chains to stabilize the unfolded protein against aggregation^25^. Here, a small protein, insulin (1.4 kDa), was used as a model protein and TFE was used as a typical stabilizer for theoretical analysis. A molecular dynamics (MD) simulation of insulin was carried out by cleaving all disulfide bonds and dissolving the protein in a TFE aqueous solution (80% in volume). Snapshots (Fig. 1a, Supplementary Fig. 3a) and the observed increase in the radius of gyration (Rg) (Supplementary Fig. 3b) and solvent-accessible surface area (SASA) (Supplementary Fig. 3c) showed that each chain of insulin gradually extended and separated. Due to the amphiphilic nature of TFE, the hydrophilic hydroxyl group in TFE tended to interact with the hydrophilic residues of proteins, and the fluoroalkyl groups in TFE preferentially interacted with the hydrophobic residues of proteins. In this way, the TFE molecules densely aggregated around the polypeptide chain of insulin and shielded the nonbonding interactions among protein chains. As a result, the number of intermolecular hydrogen bonds decreased significantly, and the hydrophobic and electrostatic interactions weakened (Supplementary Fig. 3d–f). These results suggested that the cleavage of the disulfide bonds and the presence of TFE in the protein solution could generate a stabilized unfolded protein phase by screening intermolecular interactions.

To achieve protein aggregation, destabilization of the unfolded protein surrounded by TFE is needed. Considering the good water solubility of TFE (i.e., strong hydration of TFE by water), TFE-stabilized unfolded proteins were added to water to efficiently remove the TFE molecules surrounding the proteins. According to the snapshots shown in Fig. 1b, the TFE molecules around the insulin chains rapidly diffused into the water, exposing the protein chains, which quickly approached each other to initiate aggregation (Supplementary Fig. 4a). The exchange between TFE and water was further proven by the increase in the distance between the polypeptide chains and the TFE molecules (Supplementary Fig. 4b). Furthermore, the aggregation of unfolded proteins upon TFE removal was observed by the decrease in the Rg of insulin (Supplementary Fig. 4c). The aggregation of unfolded protein was mainly driven by van der Waals and hydrogen bond interactions between protein chains (Supplementary Fig. 5), which resulted in the transformation of α-helices to β-sheets (Supplementary Fig. 4d). A large amount of *β*-sheet stacking may provide unfolded insulin with sufficient adhesion energy to achieve glue development. The underwater adhesive ability of the unfolded insulin was then demonstrated by sandwiching it into the gap between two silica sheets. According to the MD simulation, the unfolded insulin placing between two silica sheets (at 0 ns) simultaneously aggregated and interacted with the surface of silica at 500 ns (Fig. 1c). To test the adhesion strength, a steered MD simulation was conducted by applying a pull force in a direction parallel to the silica sheets. The unfolded insulin showed a high bonding force between the two layers of silica, and the tensile breaking force could reach ~1600 kJ/mol/nm (the adhesion strength was ~400 kPa), indicating the excellent underwater adhesion performance of the unfolded proteins (Fig. 1d, Supplementary Movie 1). An obvious transformation of *β*-sheets into random coils was further observed as the distance between the two silica sheets increased to 10 nm, indicating that the effective energy dissipation during the tension process may dissipate the stress in the tension to strengthen the toughness of the glue.

**Fig. 2:**
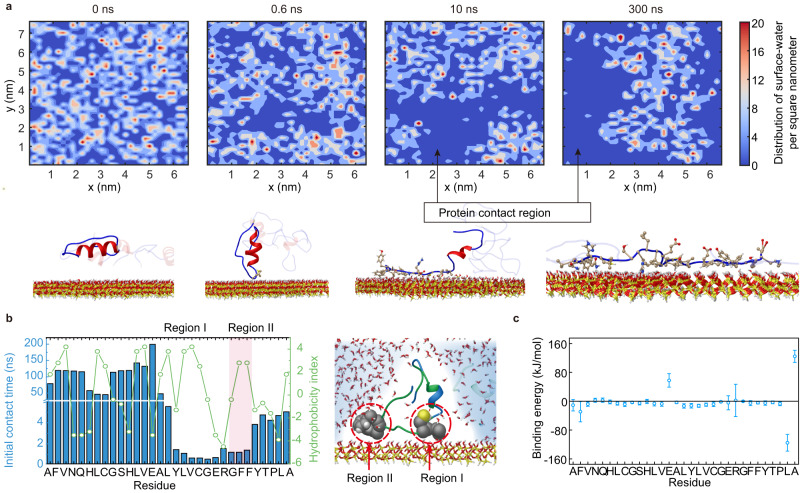
The interaction between unfolded insulin and the silica surface (MD simulation). **a** Time progression of the distribution of surface water (water molecules within a height of <0.35 nm above the surface) on the surface of silica and corresponding snapshots of unfolded insulin interacting with silica. **b** The initial contact time of each residue of insulin chain B to the silica surface, the corresponding hydrophobicity index, and the snapshot at the beginning stage of the contacting process of insulin with the surface (2 ns). The residues of two hydrophobic regions preferentially expel the bound water and interact with the silica. **c** The binding energy between each amino acid residue and the surface of silica in water. Data are mean ± S.D. *n* = 50 independent samples per group.

The presence of stable hydration layers on a substrate surface results in strong repulsion of most adhesives^26,27^. In contrast, the unfolded protein, after releasing the stabilizer (TFE), could sweep away the hydration layer on the surface of the substrate. The distribution of the hydration layer on the surface of silica in the MD simulation (Fig. 2a) clearly indicated that before the insulin chain was anchored on the silica, the silica surface was almost entirely covered with water molecules. After insulin contacted the surface for 0.6 ns, the water molecules on some discontinuous substantial portions of the surface were depleted. This indicated that the proteins initiated the breaking of the hydration layer on the silica probably through some amino acid residues of unfolded protein chains. Then, these depleted regions further expanded to form large continuous area as an increasing number of protein residues came into contact with the silica surface at 10~300 ns. The above results clearly revealed that the unfolded protein could dry the underwater surface. After penetrating the hydration layer, some regions of the unfolded insulin chain spontaneously contacted the surface and then dragged the whole chain along the surface. This whole process was accompanied by the rotation of amino acid residues to better interact with silica. Analysis of the initial contact time between each residue of insulin and silica as well as the corresponding hydrophobicity index (Fig. 2b) clearly revealed that two hydrophobic residue-enriched regions (Region I and Region II) in the random coil had the shortest contact time, which implied a preferential binding of Region I and Region II to the interface. In detail, Region I consists of leucine, valine, and cysteine, and Region II is composed of two phenylalanine residues. A snapshot of two regions interacting with the surface showed that these two regions punctured the hydration layer on the silica surface like nails and tightly adhered to it. We, therefore, concluded that forming flexible hydrophobic regions of unfolded protein serves as a precursor to dry the surface, which is consistent with previous reports on the adhesion principle of mussel foot protein^27^. (Supplementary Fig. 6) Following that, other residues began to interact with the interface based on their intrinsic properties. In particular, except for the negatively charged acidic (glutamic acid) and carbon-terminal (alanine) amino acid residues with positive binding energies, the other residues spontaneously adhered to the silica surface, as shown by their negative end binding energies (Fig. 2c). Such adhesion is co-contributed from gradually increasing hydrogen bonding as well as energetically favored van der Waals (vdW) and electrostatic interactions (Supplementary Fig. 7). Among these residues, lysine showed the highest binding ability to silica due to the electrostatic interaction between the weakly negatively charged surface of silica and lysine.

### Construction of protein-based underwater glue

**Fig. 3:**
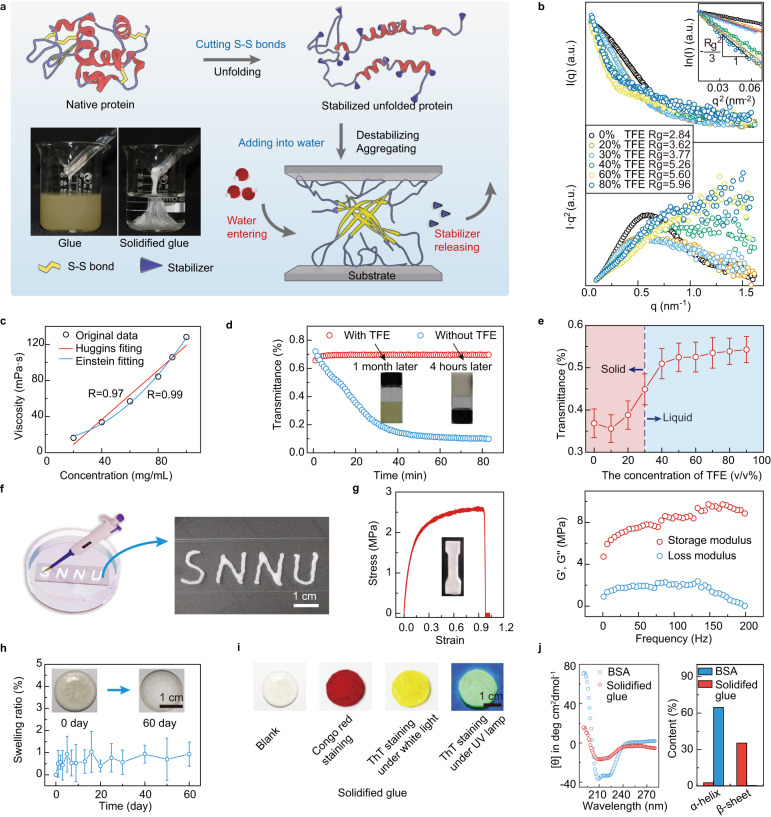
The unfolding and solidifying of BSA. **a** Schematic of the unfolding-aggregating strategy to transform common proteins into underwater glue and photograph of the unfolded BSA and its aggregation bonding on the bottom of a beaker filled with water. **b** Scattering plots (*I*~*q*) and corresponding Kratky plots (*I*·*q*^2^ ~ *q*) of BSA in different concentrations of TFE solution with TCEP added. The Rg was calculated according to the Guiner curves (ln(I) ~ q^2^) (inset). *I* scattering intensity, *q* scattering vector. **c** BSA concentration dependence of the viscosity change of unfolded BSA in TFE solution (80% in volume). The result shows better fitting with the Einstein equation (*R*^2^ = 0.99) than with the Huggins equation (*R*^2^ = 0.97). **d** Dynamic relative light transmittance (600 nm) of unfolded BSA with and without TFE added, and the corresponding photograph of stable unfolded BSA solution with TFE added after 1 month (left) and coagulated unfolded BSA without TFE added after 4 h (right). **e** Light transmittance (wavelength = 600 nm) of unfolded BSA solution with TFE added at different concentration. **f** The word SNNU written on an underwater glass slide by using the unfolded BSA glue. **g** Stress–strain curve of the solidified unfolded BSA glue and a photograph of the dumbbell sample to be tested. Frequency dependence of the storage modulus G′ (solid symbols) and loss modulus G′′ measured by DMA with 1% strain. **h** Swelling ratio of the solidified unfolded BSA glue soaked in water. **i** Photograph of ThT and Congo red staining of the solidified unfolded BSA glue. **j** CD spectra of the unfolded BSA and its aggregation triggered by water and the corresponding secondary structure quantitative analysis by BeStSel. All data in (**e**, **h**) are mean ± S.D. *n* = 3 independent samples per group.

The MD simulation results for insulin were further experimentally verified by manipulating the unfolding aggregation of proteins, which transform versatile common commercial proteins into strong underwater adhesives. A feature of this concept is the stabilization of unfolded protein chains by small molecular stabilizers and the induction of adhesive aggregation of unfolded proteins by removal of the stabilizer. As shown in Fig. 3a, tris(2-carboxyethyl)phosphine (TCEP), a reductant that cleaves disulfide bonds under mild conditions^28^, was added to different proteins in TFE aqueous solutions to obtain unfolded protein chains. These resultant solutions were then mixed with water to induce the removal of the TFE stabilizer and subsequent adhesive aggregation of the unfolded protein chains in water. As listed in Table 1, most proteins, such as insulin, *α*-lactalbumin, lysozyme, *β*-lactoglobulin, ovalbumin (OVA), bovine serum albumin (BSA), human serum albumin (HSA), and so on, can solidify and adhere to substrates in water (Supplementary Fig. 8, 9), indicating the successful transformation of common proteins into underwater glue. In contrast, a small number of proteins, including *β*-galactosidase, amylase, collagenase, and *β*-galactosidase, cannot solidify in water. This significant difference is a result of the differentiated amyloid assembly propensity of proteins. Based on ZipperDB, a web database tool to predict amyloid fibril-forming segments^29^, the energetic fit of amino acid residues in each protein was evaluated by Rosetta energies. Because amino acid residues with Rosetta energies equal to or <−23 kcal/mol were self-complementary and had a high amyloid assembly propensity^29^, the proportion of high amyloid-propensity (*P*_HAP_) segments (Rosetta energy ≤ −23 kcal/mol) in the whole protein sequence was statistically analyzed. The results showed that the proteins with *P*_HAP_ values higher than 16.77% could be transformed into underwater glue because these proteins contain more segments that tend to undergo amyloid assembly. Thus, as shown in Table 1, at least 14 types of common proteins with high *P*_HFP_ values are effective candidates for transformation into underwater glue. To summarize, the unfolding-aggregating approach allows for the transformation of common commercial proteins into underwater glue. This process requires the protein to meet particular sequence criteria, such as having a *P*_HAP_ value of at least 16.77%.

**Table 1:** Solidification properties of different proteins and their corresponding *P*_HAP_

Index	Protein name	*P*_HAP_	Solidification properties
1	Insulin	43.75%	Yes
2	Trypsin	32.94%	Yes
3	OVA	30.16%	Yes
4	Hemoglobin	25.00%	Yes
5	Lysozyme	23.39%	Yes
6	*α*-Lactalbumin	23.02%	Yes
7	HSA	20.26%	Yes
8	Chymotrypsin	19.62%	Yes
9	Transferrin	19.32%	Yes
10	*β*-Lactoglobulin	18.42%	Yes
11	Myoglobin	18.40%	Yes
12	BSA	18.36%	Yes
13	Lactoferrin	17.14%	Yes
14	Thyroglobulin	16.77%	Yes
15	*β*-Glucosidase	15.27%	No
16	Amylase	13.96%	No
17	Collagenase	13.79%	No
18	*β*-Galactosidase	12.66%	No

We then chose one of the least expensive proteins, BSA, as the model protein to explore the microscopic structure and performance of the resultant underwater glue. As shown in Fig. 3a, upon diffusion of the stabilizer into the water, the unfolded BSA chains pre-stabilized by TFE were destabilized and transformed into glue that could tightly adhere to a glass rod and the bottom of a beaker upon stirring after injection into water. During the unfolding process, most disulfide bonds in BSA were reduced by TCEP, as determined from the sharply decreased peak between 480~570 cm^−1^ in the Raman spectra for disulfide bond (Supplementary Fig. 10a)^30^. This was further supported by the increase in *N*-(1-pyrenyl) maleimide (NPM) fluorescence assay^31^, in which the conjugation of NPM with the free thiol groups from reduced disulfides significantly enhanced the fluorescence intensity at 380 nm (Supplementary Fig. 10b). Protein unfolding also caused a decrease in the intramolecular packing density of native proteins, as determined by synchrotron small-angle X‐ray scattering (SAXS) (Fig. 3b). With an increase in the TFE concentration from 0% to 30%, the Rg of BSA increased slowly from 2.84 nm in the native state to 3.77 nm, followed by a sudden increase from 3.77 nm to 5.26 nm when the TFE concentration was higher than 30%. This result indicated that after disulfide bond reduction by TCEP, TFE induced a swelling effect on proteins to increase their Rg^25^. At a low TFE concentration (<30%), the treated protein gave rise to a distinct peak in the *Iq*^2^~*q* curve (Kratky plot)^32^ obtained via SAXS, indicating that the protein structure was similar to that of the native protein. Once the concentration of TFE was higher than 30%, the Kratky plots of treated BSA showed a plateau, suggesting that the treated BSA was noticeably unfolded. Protein unfolding was further proven by viscosity analysis (Fig. 3c, Supplementary Fig. 10c). The change in viscosity with the concentration of BSA in a TFE solution (80% in volume) indicated a better fitting with the Einstein equation (*R*^2^ = 0.99) than with the Huggins equation (*R*^2^ = 0.97). This suggested that the unfolded BSA adopted a chain-like structure instead of its native spherical structure^33^. In such an unfolded state, the *α*-helical structure was even more significant than that from native BSA, indicating a strong stabilization effect from TFE on the maintenance of unfolded protein conformation (Supplementary Fig. 10d). The stabilizing effect of TFE on unfolded proteins was further confirmed by measuring the change in relative light transmittance (at a wavelength of 600 nm) of unfolded BSA. The relative light transmittance of unfolded BSA stabilized with TFE was almost unchanged, but the transmittance of unfolded BSA without TFE added decreased rapidly due to uncontrollable aggregation and phase separation (Fig. 3d). The unfolded BSA stabilized with TFE can be kept in the liquid state for over 1 month at room temperature, and the lowest volume ratio of TFE in water to keep such liquid state was then measured as 30% (Fig. 3e, Supplementary Fig. 11). In contrast, the unfolded BSA with the TFE concentration <30% or without adding TFE solidified after 4 h.

Following stabilizer-controlled unfolding, the solidification of unfolded proteins to form underwater glue could be triggered by the stabilizer (TFE) removal in water. Upon injection into water for 5 min, the glue solidification occurred sufficiently to afford a written pattern tightly adhered on the glass surface that withstand strong rinsing with tap water from the faucet (Fig. 3f, Supplementary Movie 2). The protein adhesive presented good stretching and fracture stability, as the protein glue solidifying in a dumbbell-like shape could be stretched more than 100% before breaking, with a breaking strength of ~2.5 MPa and good flexibility as reflected by a low Young’s modulus of 14.3 MPa (Fig. 3g). Dynamic mechanical analysis (DMA) of the solidified BSA glue showed that the storage modulus (G′) was always higher than the loss modulus (G′′). This proved that the elastic strain was greater than the friction loss, reflecting a possible network structure inside the solidified BSA glue. The existence of a network structure was also supported by the good anti-swelling property of the solidified glue, since the volume of the solidified BSA glue remained almost unchanged after soaking in water for two months (Fig. 3h). Taken together, the above results demonstrated that controllable underwater aggregation of unfolded protein chains can lead to the formation of a new kind of robust underwater protein glue with high mechanical strength and remarkable anti-swelling properties.

The aforementioned MD results indicated that the formation of the protein adhesive was governed by *β*-sheet-directed aggregation. This analysis was further supported by the structural characterization of the solidified unfolded BSA glue. With Congo red staining, a visible red color was present on the glue due to the specific binding of Congo red with the *β*-sheet structure inside the adhesive (Fig. 3i)^34^. Further thioflavin-T (ThT) staining of the solidified glue resulted in significant enhancement of the ThT fluorescence intensity, indicating the possible presence of amyloid-like structures in the protein glue^35^. In addition to the staining assay, spectral analysis including far-ultraviolet circular dichroism (far-UV CD) and Fourier-transform infrared spectroscopy (FTIR), provided direct evidence of the conformational change during protein unfolding and aggregation. According to BeStSel^36^, an online tool that uses CD spectra to quantify the second structure, solidified unfolded BSA glue displayed a 35% increase in β-sheet compared with unsolidified unfolded BSA glue (Fig. 3j). Deconvolution of the amide I FTIR spectra during the solidification further demonstrated that the characteristic *β*-sheet peaks (1630 and 1692 cm^−1^) increased significantly compared to those of unfolded BSA (Supplementary Fig. 12)^28^. This *β*-sheet-directed amyloid-like aggregation was also observed in the adhesives prepared from proteins other than BSA, such as insulin, lysozyme, OVA, lactoglobulin, and lactalbumin, as reflected by Congo red and ThT staining as well as CD and FTIR spectra (Supplementary Figs. 13–16). As a typical feature in silkworms^37^, spider silk^38^, and wool^39^ presenting robust mechanical strength, *β*-sheet stacking inside the protein adhesive is an important structural factor contributing to the strong mechanical strength and reliable stability of the protein glue in water.

Furthermore, we found that urea was also effective in stabilizing unfolded proteins in addition to TFE. As urea tends to bind to protein hydrophobic domains similarly to TFE^40^, it also prevents unfolded proteins from aggregating as well. Upon urea-stabilized unfolded proteins being added to water, urea molecules diffuse and expose the hydrophobic domain in the proteins, forming amyloid-like aggregates and adhesion forces (Supplementary Fig. 17).

### Adhesion performance of unfolded BSA as an underwater glue

**Fig. 4:**
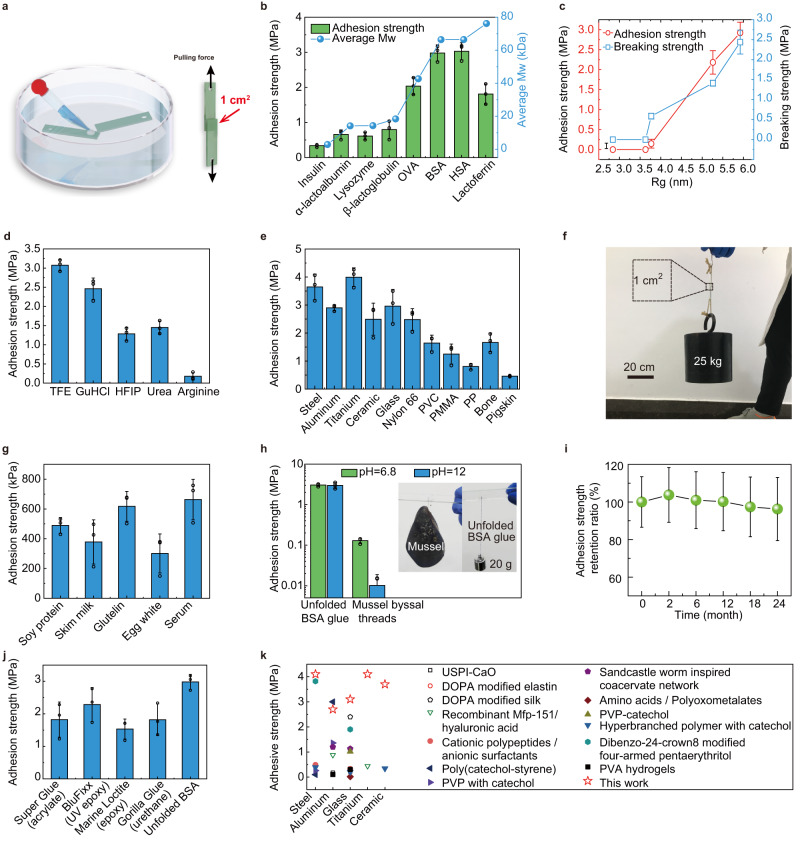
Performance of unfolded protein-based underwater glue. **a** Schematic of the measurement of the underwater adhesive strength of unfolded BSA glue. All adhesion tests were performed in water. **b** The underwater adhesion strength from different protein-based glues. The corresponding average molecular weight (Mw) of the polypeptide chains contained in each protein is also noted. **c** The adhesion strength of unfolded BSA glue to glass sliders and the breaking strength of the solidified glue at various degrees of unfolding. The Rg was employed to quantify the unfolding degree of a protein. **d** The adhesion strength of unfolded BSA prepared with different stabilizers. **e** The adhesion strength of unfolded BSA glue applied to bond a variety of materials. **f** Photograph of unfolded BSA-bonded glass slides lifting a 25 kg weight (with a bonding area of 1 cm^2^). **g** The adhesion strength of the glue to bond glass fabricated from the crude protein-containing mixture (reductant: TCEP, stabilizer: urea). **h** Comparison between unfolded BSA glue and mussel byssus bonding to glass sliders and their alkali resistance. Inset photographs showing a mussel (34 g) hanging on glass slides by four byssal threads (left) and a weight (20 g) hanging on glass slices by a string bonded to unfolded BSA (right). **i** Long-term bonding stability of unfolded BSA glue in water. **j** Comparison of adhesion strength of the unfolded BSA glue with commercial glues in water. **k** Comparison of the adhesion strength (lap shear strength) of unfolded BSA glue and those from previous reports^3,13,41,58–67^. Only the underwater glues were considered, of which all the steps of the adhesion process were performed underwater. (USPI urushiol grafted soy protein isolate, DOPA polydopamine, PVP polyvinyl pyrrolidone, PVA polyvinyl alcohol). All data in (**b**–**e**, **g**–**j**) are mean ± S.D. *n* = 3 independent samples per group.

By sandwiching 10 μL of the protein glue between two glass slides with an overlapping area of 1 cm^2^ for 24 h in water, the underwater adhesion strength of the bioglue was directly measured in a water-filled container (Fig. 4a, Supplementary Fig. 18). By systematically modulating the preparation conditions, including the pH of the system; the concentrations of TFE, BSA, and TCEP; and the reaction time, the bonding strength of the glue to glass was optimized to be approximately 3 MPa in water (Supplementary Fig. 19). The unfolded BSA glue solidified to produce considerable instant adhesion strength (~100 kPa) in 100 s (Supplementary Movie 3), and reach the maximum value after about 10 h (Supplementary Fig. 20). Notably, the underwater adhesion ability was closely dependent on the average molecular weight of each peptide chain of the protein (Fig. 4b, Supplementary Fig. 21). The larger the molecular weight was, the stronger the adhesion ability was. This may be because the cohesive force of the solidified glue enhanced with the increasing molecular weight of the proteins. However, the adhesion strength further decreased when the molecular weight was larger than 70 kDa, since the increased cohesion in high-molecular-weight protein chains weakened the infiltration ability of the glue (through the hydration layer on the substrate), resulting in poor contact between the glue and the substrate^41^. Aside from the molecular weight of the protein, the unfolding degree of the protein also has a significant impact on the adhesive strength. As illustrated in Fig. 4c, the greater the unfolding degree, the stronger the mechanical and adhesion strength. It’s possible to expose more hydrophobic groups and give better molecular chain flexibility for interfacial adhesion when proteins are more unfolded. Highly unfolded proteins, on the other hand, allow a single protein chain to participate in several nucleation events, resulting in the formation of a network structure that ensures cohesiveness, as seen by the rising breaking strength of the formed glue. This demonstrates that a significant degree of unfolding is critical for a protein’s underwater adhesion.

A variety of reagents can be selected for the cleavage of unfolded disulfide bonds and stabilizing the unfolding state of protein. The disulfide reductants, including TCEP, mercaptoethanol, thioglycerol, cysteine, and glutathione, could cleave the disulfide bonds of BSA and trigger the formation of underwater glue. The bonding strength was dependent on the reducing capacity of reductants. For this reason, TCEP, mercaptoethanol, and thioglycerol, which exhibit higher activity toward disulfide reduction, provided higher adhesion strength than cysteine and glutathione (Supplementary Figs. 22 and 23)^42^. Additionally, there are also several alternate choices for stabilizing agents, such as TFE, hexafluoroisopropanol (HFIP), urea, guanidine hydrochloride (GuHCl), and arginine (Fig. 4d)^43^. Their corresponding adhesion strengths are distinctly different. The TFE-stabilized unfolded BSA presented the highest strength since the binding ability of TFE to proteins was higher than that of the other stabilizers^44^. Numerous reducing agents and stabilizers are available for use in a variety of application contexts. For example, TFE/TCEP-containing glue is appropriate in some emergency circumstances to achieve the highest adhesive strength, GuHCl/TCEP-containing glue may be appropriate for industry applications, and the urea/cysteine system, with low toxicity to bioactive compounds and similar functions for stabilizing unfolded proteins, is suitable for application in living organisms.

As a general adhesive to virtually arbitrary materials, unfolded BSA glue showed remarkable underwater adhesion strengths (Fig. 4e), with the ability to bond a variety of synthetic materials and biological tissues together, including steel (3.64 MPa), aluminum (2.89 MPa), titanium (3.99 MPa), glass (2.96 MPa), ceramic (2.49 MPa), nylon 66 (2.48 MPa), polyvinyl chloride (PVC) (1.64 MPa), poly (methyl methacrylate) (PMMA) (1.25 MPa), pig bone (1.67 MPa) and pigskin (0.46 MPa). Consistent with the MD simulation results (Supplementary Fig. 24), the adhesion strength to polypropylene (PP) (0.81 MPa) was weaker than those of other hydrophilic materials because of the lack of strong interaction between PP and unfolded proteins. The unfolded BSA glue also performed the bonding behavior well in water between two different types of materials typically including glass-steel (3.11 MPa), glass-ceramic (3.13 MPa), glass-nylon 66 (3.31 MPa), titanium-bone (2.03 MPa) and bone-skin (0.42 MPa) (Supplementary Fig. 25). The corresponding adhesion strength between two kinds of materials is determined by that of the weak side. In another case, two glass slides bonded with the glue at the bonding area of 1 cm^2^ were sufficient to lift a 25 kg weight (Fig. 4f), and the glue-bonded nylon 66 boards could bear the weight of an ~75 kg person (with a 25 cm^2^ of bonding area) (Supplementary Movie 4). As another feature distinct from existing strategies, our method not only effectively transformed pure proteins into underwater glue but also efficiently converted biological mixtures or crude protein mixtures, typically including soy protein, skim milk powder, glutenin, egg white, and serum, into underwater adhesives. Although their adhesion strengths were weaker than those of pure proteins (Fig. 4g), the low cost and high accessibility of these materials are beneficial for some high-dose applications. In this way, even ordinary citizens can use high-protein ingredients to prepare underwater glue in a kitchen. In addition to working in aqueous solutions, the glue also exhibited remarkable adhesion in a dry environment (Supplementary Fig. 26) and various strong polar organic solvents (Supplementary Fig. 27). The stronger the polarity of the solvent and the lower the viscosity of the solvent, the stronger the adhesion strength in the corresponding solvent. Notably, the adhesion strength in ethanol and THF was higher than that in water. This may be due to the low viscosity and high solubility of TFE in these solvents, which led to a thorough removal of TFE around the unfolded proteins.

Our glue showed robust stability in extreme environments such as pure organic solvent, low/high temperatures (e.g., 0 °C, 100 °C), acid/base solutions (e.g., pH = 3, pH = 12), 5% saline (7 days), sonication (120 W, 1 h), and reciprocating pull and pressure (±100 N, 500 times), showing highly stable adhesion strength without obvious loss (Supplementary Fig. 28). In the dry environment, such glue can also work well at extreme high (200 °C) and low temperature (in liquid nitrogen) (Supplementary Fig. 29). Our material further showed better stability than conventional biological adhesives. For instance, under neutral pH, the adhesion strength of mussel byssal threads was measured to be ~131 kPa^45^, which is much less than that of unfolded BSA glue (Fig. 4h, Supplementary Fig. 30). In particular, when the solution pH was increased to 12 and the experimental system was soaked in such a solution for 48 h, there was no change in the adhesion strength of the unfolded BSA glue, while the adhesion of mussel byssal threads almost disappeared due to the easy destabilization of the catechol group in the mussel adhesive at high pH. As a result, upon washing with tap water, the byssal threads adhered to the glass sheet were easily washed away, but the solidified protein glue could not be peeled off (Supplementary Movie 5). A long-term bonding stability test further proved that our adhesive could bond the glass slides in tap water for at least 2 years without obvious loss of the adhesion strength (Fig. 4i), which is longer than the stability of currently reported underwater glues^13^. Comparing with common commercial glues such as Super Glue (acrylate), BluFixx (UV epoxy), Maine Loctite (epoxy), and Gorilla Glue (urethane), the adhesion strength of unfolded BSA glue was the highest (Fig. 4j). While commercial epoxy-type adhesives can produce megapascal levels of strength as well, it is due to surface water on the substrate being released as a result of extrusion, which allows the glue to interact with substrates and produce strong adhesion. A demonstration of this is the fact that commercial glues cannot bond the hydrogel (which contains 99 percent water), since the hydrogel can constantly seep water molecules to the surface during the extrusion, but our unfolded protein glue can, and its strength is even greater than that of the hydrogel itself (Supplementary Fig. 31). Moreover, unfolded BSA glue has a significantly higher adhesion strength than most protein-based adhesives and even compares to some synthetic adhesives (Fig. 4k).

**Fig. 5:**
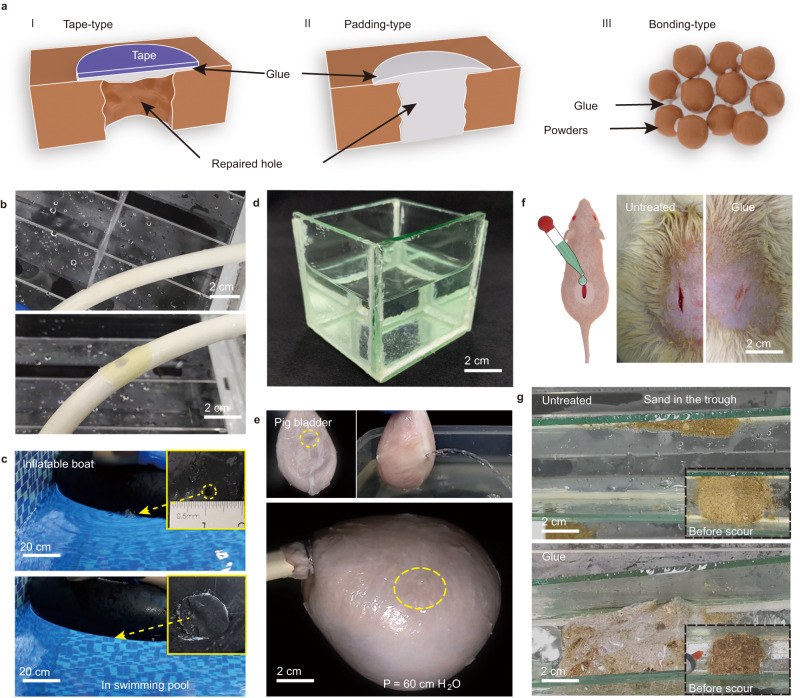
Applications of the unfolded BSA glue. **a** Schematic of three types of application manners: tape-type, padding-type, and bonding-type. **b** Patching of a rubber pipe with a hole (5 mm in diameter) by a rubber sheet coated with the unfolded BSA glue. **c** Repairing of the inflatable boat with a broken hole (2 mm) by a rubber sheet coated with the unfolded BSA glue. **d** A glass container constructed by using the unfolded BSA glue. **e** A pig’s bladder with a 5 mm hole patched by the unfolded BSA glue, which can withstand a pressure of 60 cm H_2_O. The dotted yellow circle is the location of the hole. **f** The unfolded BSA glue works as a tissue glue to close incisions in rats. **g** The performance of the unfolded BSA glue for rapid sand fixation in a glass trough.

Because of its reliable adhesion behavior, our glue has excellent promise in practical applications. The application methods can be divided into three types: tape-type, padding-type, and bonding-type (Fig. 5a). In the tape-type application, the unfolded BSA glue can repair defects on a water container or pipeline in real time. Here, we applied it to a rubber sheet to then patch a leaking rubber pipe with a 5 mm hole. Approximately 1 min later, the patched rubber pipe worked well under 100 kPa of water pressure (approximately equivalent to the working pressure of a pressure cooker) (Fig. 5b, Supplementary Movie 6). Likewise, the unfolded BSA glue can be applied to deal with some emergencies, e.g., repairing the inflatable boat with a broken hole (2 mm in diameter) to recover normal rowing (Fig. 5c, Supplementary Movie 7).

In the padding-type application, a glass container can also be glued by the unfolded BSA adhesive to hold water without leaking (Fig. 5d). Further, a pig’s bladder with a 5 mm hole could be patched by unfolded BSA glue to withstand 60 cm H_2_O of pressure, which is higher than the safe intravesical pressure limit (40 cm H_2_O^46^) (Fig. 5e, Supplementary Movie 8). A number of clinically used tissue adhesives, such as fibrin, gelatin resorcinol, and cyanoacrylates, are considered inadequate due to mechanical properties and potential toxicities^47^. Differently, this protein-based glue has adjustable low modulus from tens of megapascals to tens of kilopascals (Supplementary Fig. 32) matching with different human tissues. In vitro, experiment showed that the unfolded protein glue had no obvious effect on the structure of serum proteins (Supplementary Fig. 33) and can be completely hydrolyzed by trypsin after 12 h **(**Supplementary Fig. 34). According to cytotoxicity tests using neuronal cells (HT22) (Supplementary Fig. 35) and various visceral cells (HepG2, HUH-7, HL-7702, and HEK293) (Supplementary Figs. 36–38), hemolysis (Supplementary Fig. 39), acute toxicity (Supplementary Table 2), and pathology examinations of organs (Supplementary Fig. 40), the urea-stabilized unfolding BSA adhesive does not exhibit obvious biotoxicity. To further demonstrate the usefulness of protein glue, the in vivo degradability of the glue was also determined. By mixing Cy5.5-labeled BSA into the glue, real-time protein degradation was observed in SD rats (Supplementary Figs. 41, 42). As reflected in the decrease in the fluorescence range of the glue sample (5 mm × 5 mm × 1 mm), the glue sample was totally degraded after 50 days. Confocal microscopy and H&E staining of tissue sections revealed that a large number of blood vessels are newly formed near the implantation site, and a large number of cells infiltrate the sample to degrade it (Supplementary Figs. 43, 44). Based on CD68 staining of macrophages (Supplementary Fig. 45), it was found that after 10 days of implantation, macrophages infiltrated the protein glue. After 50 days, macrophage numbers decreased significantly, indicating that macrophages play a major role in degradation. These results indicate the unfolded protein glue degrades in vivo well depending primarily on macrophages. Also, serum biochemical analyses were conducted during the entire implantation period (Supplementary Table 3) of rats, and the results indicated that the various indicators in the blood were within the normal range, which indicates that the glue has almost no influence on the function of organs and tissues of rats, as well as the metabolic state. Further, the unfolded protein glue was used to close the incision of SD rat. The result showed that the incision can be closed within 60 s without blood or fluid leakage (Fig. 5f, Supplementary Movie 9). After 7 days, wounds treated with the unfolded BSA glue completely healed without visible scars, and the wound fracture strength in the unfolded BSA glue group was higher than those of the sutures and untreated groups (Supplementary Fig. 46). Although these cell and animal experiments have provided preliminary evidence of the biosafety of glue, further research is still needed to prove its usefulness in clinical applications.

In the bonding-type application, this glue could bond sand together rapidly to withstand flushing with water (Fig. 5g, Supplementary Movie 10), demonstrating its potential to quickly repair a leaky dam during a flood. In addition, a glue composed of inexpensive soy protein was used to bond versatile powder-like materials to bulk materials. As a result, protein glue could be utilized to fabricate a series of solid blocks with molding into different shapes by bonding microorganisms (e.g., *chlorella* and *yeasts*), pine sawdust, corn stalk powder, cellulose powder, shrimp shell powder, and chitosan powder together (5% of the powder in volume), with bending strengths higher than ~10 MPa (Supplementary Fig. 47).

### Solidification and adhesion mechanism of the unfolded protein glue

**Fig. 6:**
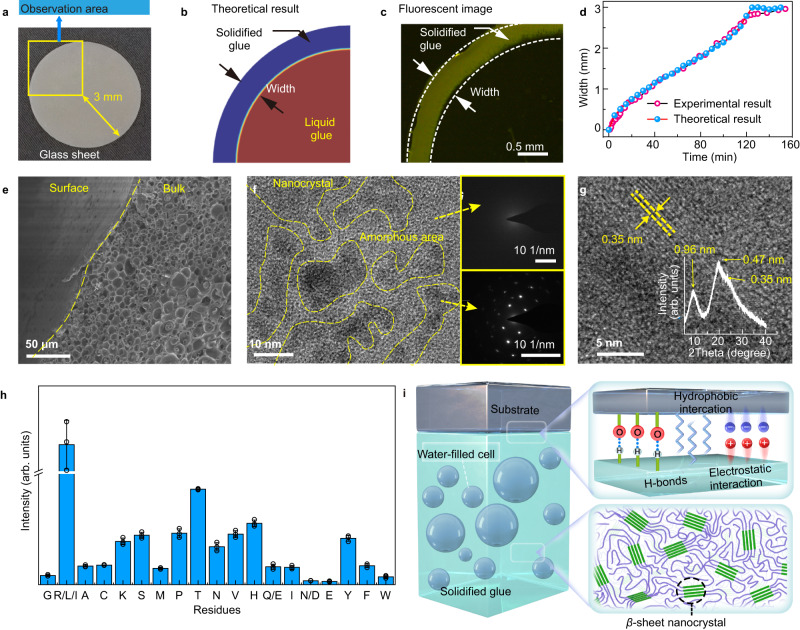
Solidification process of the unfolded BSA glue and characteristics of the inner structure and surface chemistry of the solidified glue. **a** Photograph of a round glass sheet (6 mm in diameter) that was used to analyze the solidification process of the unfolded BSA glue. The observation area is marked with a yellow box. The FE simulated results (**b**) and ThT staining fluorescence image (**c**) of the glue solidification between two glass slides after immersing in water for 10 min, and the width change of the solidified glue ring against time calculated from the above two methods (**d**). **e** Field emission SEM (FE-SEM) images of the highlighted section of the glue after solidification. The upper left region corresponds to the surface of the glue bonded to the glass slide, and the lower right region corresponds to the inner morphology of the solidified glue. **f** The ultrathin-section TEM image of the solidified glue in bulk. The light area is amorphous, and the black areas are nanocrystals. The corresponding selected area electron diffraction (SAED) patterns were displayed on the right. **g** Local magnification of the black area, and XRD pattern of the freeze-dried solidified glue (inset, Bruker D8 Advance, Cu target, wavelength = 0.154 nm). **h** Amino acid composition analysis on the surface of the solidified BSA glue as characterized by TOF-SIMS. Data are mean ± S.D. *n* = 3 independent samples per group. **i** Schematic of the solidified glue structure, where the green cuboid bulk represents the nanocrystalline region of *β*-sheet aggregation, and the blue curve represents the amorphous region. The experiments in (**c**, **e**–**g**) were repeated independently at least three times with similar results.

The high adhesion strength and robust stability in water (also in other environments) of our protein glue can rationally be attributed to both strong bulk cohesion and interfacial adhesion. First, strong bulk cohesion is closely related to the solidification-induced microscopic morphology evolution of the unfolded protein glue. The solidification of unfolded protein glue between two round glass sheets (Fig. 6a) was first simulated by the finite element (FE) method and then experimentally verified by luminescence microscopy. According to previous MD simulation results, water molecules are gradually exchanged with the TFE surrounding the unfolded protein chains to remove the stabilizer. As a result, water diffused from the outside into the glue, accompanied by the aggregation of unfolded protein chains. Thus, an Fick’s second law-based dichotomy model was then constructed for theoretical analysis of the solidification process, in which the glue was considered to solidify immediately as long as the concentration of water molecules in the glue was greater than 38.9 mol/L (70% in volume) (as also judged from Fig. 3e). As water molecules diffused into the glue, the outer glue being the first to contact with water would solidify and proceed into a ring-like pattern (Fig. 6b, Supplementary Fig. 48). The simulated results of the solidification model are similar to those observed from the ThT-stained fluorescence photographs (Fig. 6c, Supplementary Fig. 49). The development trends in the width of the glue solidification ring obtained by the FE and fluorescence images are highly coincident (Fig. 6d), indicating that the solidification process was driven by the diffusion of water molecules, by which the TFE molecules around the protein were gradually expelled.

The FE results further indicated the change in water molecule concentration along the solidified glue showed a fast-to-slow gradient from the edge to the interior of the glue along the radial direction (Supplementary Fig. 50). This is consistent with the finding that the diffusion coefficient of water molecules increased significantly after the unfolded protein glue solidification, as measured through attenuated total reflection Fourier-transform infrared (ATR-FTIR) (Supplementary Fig. 51)^48^. It was then speculated that the high diffusion coefficient of water in the solidified glue was related to the formation of a large number of pores, which provided a high-speed channel for water molecules to pass through. As shown in the cross-sectional scanning electron microscopy (SEM) (Fig. 6e), different from the smooth contact surface induced by the substrate plane, the internal solidified glue consisted of a large number of cells ~10 μm in size that connected with each other. These cells corresponded to the space formed by the diffusion of stabilizers and the aggregation of unfolded proteins (Supplementary Fig. 52). They were filled with water to facilitate fast water diffusion, forming a porous solid-liquid foam similar to sandcastle glue. Such a structure has been considered an important building block to achieve robust adhesion through osmotic prestressing and hydraulic toughening^49^. Ultrathin-section transmission electron microscopy (TEM) further revealed that there were some nanocrystals with a lattice constant of 0.35 nm distributed sporadically in the internal structure of the solidified glue, and it was hard to find any nanocrystals in the light-colored area (Fig. 6f, g). The powder X-ray diffraction (XRD) results showed that the dispersed nanocrystals exhibited a predominant diffraction peak at 19.2° (0.46 nm) associated with the interchain distance within the hydrogen-bonded *β*-sheets and a diffraction feature at 23.9° (0.35 nm) as the indication of the in-plane spacing between adjacent *β*-sheets^50^. The appearance of nanocrystals inside the non-crystallized bulk phase indicated that the interior morphology of the solidified BSA glue is a sea-island structure, in which *β*-sheet nanocrystals are embedded in amorphous continuous phase. Because amyloid crystals (*β*-sheet crystals) are the most thermodynamically stable assembly of proteins, the relaxed and flexible chains stabilized by TFE gradually destabilized and assembled into nanocrystals^51^. The *β*-sheet nanocrystals then behaved as crosslinking locks to provide strong internal cohesion among the polypeptide chains, enabling the amorphous domains to stretch significantly with high robustness^37^.

Besides tough bulk cohesion, the strong interfacial adhesion of the unfolded protein glue was resulting from a series of functional groups including -NH_2_, -COOH, -OH, -SH, -(CH)_n_, -CH_3_, and aromatic rings on the glue surface, which supported the co-contribution from ligand bonds, electrostatic interactions, hydrogen bonds, and hydrophobic interactions with metal, organic and inorganic material surfaces^52^. Actually, as seen from the results of time-of-flight secondary ion mass spectrometry (TOF-SIMS) (Fig. 6h, Supplementary Fig. 53, Supplementary Table 4) and X-ray photoelectron spectroscopy (XPS) (Supplementary Fig. 54), the solidified glue surface was enriched with various chemical groups. These abundant hydrophilic and hydrophobic chemical groups on the surface could thus provide a large number of sites for hydrogen bonding as well as electrostatic and hydrophobic interactions, thus providing considerable interfacial adhesion. We thus attributed the strong and robust underwater adhesion of this protein-based glue to two factors (Fig. 6i). One is the high bulk strength induced by a large number of *β*-sheet-rich nanocrystals in the interior of solidified glue, and the toughness of which is improved by the presence of water-filled cells. And the other is the abundant chemical groups on the substrate-contacting glue surface to afford a large number of various and strong noncovalent interactions.

## Discussion

In summary, we overcome the limit that only some specific proteins exhibit underwater adhesion by providing a universal unfolding-aggregating strategy to transform common commercial proteins into underwater glue. Unlike other nature-inspired underwater glues that need specific functional groups, polymer network structures, or geometries of materials^4^, our glue is fabricated from fairly common commercial proteins through solvent-mediated stabilization-aggregation of biopolymer chains. By this general strategy, dozens of common proteins and their mixtures are first unfolded and stabilized by solvent to suppress aggregation, and then, unfolded proteins can undergo controllable amyloid-like aggregation in water through gradual diffusion of solvent stabilizers. During this process, the exposed flexible hydrophobic region of unfolded protein chains can dry the wetted surface, followed by dynamic adjustment of both hydrophilic and hydrophobic residues to preferably interact with solid surfaces and thereby provide strong interfacial adhesion. Furthermore, the solidified glue exhibits a sea-island structure, in which amyloid nanocrystals provide strong and substantial crosslinking points to ensure tough internal cohesion. The contributions from both robust interfacial adhesion and cohesion endow this glue with high strength and excellent adhesion stability against extreme conditions (at least 2 years of underwater immersion). Such adhesive can be applied as a tape-type, padding-type, and bonding-type glue to meet different demands. This strategy confirms that controlling conformational changes in biopolymer chains is quite significant for underwater adhesion instead of introducing only some specific groups or structures. This may open up a new field for conformation-directed underwater adhesion.

## Methods

### Preparation of protein glue

The protein (1.25 g of BSA, insulin, α-lactalbumin, lysozyme, β-lactoglobulin, OVA, HSA, lactoferrin, BSA, myoglobin, hemoglobin, trypsin, transferrin or crude protein) was dissolved in 80% (v/v) TFE, 50% (w/v) urea, 50% (w/v) GuHCl or 20% (w/v) arginine aqueous solution (4.5 mL), followed by the addition of 500 μL of a disulfide bond reducer, such as TCEP (600 mM, pH = 2), cysteine (600 mM, pH = 2), GSH (600 mM, pH = 2) and mercaptoethanol (600 mM, pH = 2). Stirring the reaction system with a glass rod to make it fully mixed, and the unfolded protein glue can be obtained after standing at room temperature for 4 h.

### Synchrotron small-angle X-ray scattering

SAXS data were obtained on the 1W2A beamline a Beijing Synchrotron Radiation Laboratory. Scattered X-ray intensities were measured by a Mar 165 CCD (2048 × 2048 pixels) at an X-radiation wavelength (*λ*) of 1.54 Å. The distance between the sample and the detector was set at 3 m. Each sample was measured with an exposure time of 100 s. The scattered signals of BSA (10 mg/mL) in 0% ~80% TFE aqueous solutions containing TCEP (60 mM) were measured. The blank groups were the corresponding aqueous solutions without TCEP. The acquired raw data were analyzed by FIT2D.

### Viscosity measurements

The viscosities of the native and unfolded BSA solutions of different concentrations were measured using a DVS+ Digital Viscometer (Brookfield) with an RV2# rotator at a speed of 10 r.p.m. The measured data were fitted by the Huggins equation and the Einstein equation. The change in viscosity with increasing concentration of the chain-like structures can be fitted using the Huggins equation. In contrast, the viscosity of spherical particles in solution accords with the Einstein equation. The *R*^2^ calculated from the fittings of the Huggins equation and the Einstein equation was used to evaluate whether the protein is similar to a chain-like (unfolded) or spherical (native) structure.

### Measurement of dynamic swelling kinetics

The solidified BSA glue sample (10 mm diameter 3.5 mm height) was weighed after removing the water on the surface with filter paper and then immersed in phosphate-buffered saline (PBS). At predetermined intervals ranging from 1 day to 2 months. The swollen samples were removed from the PBS, and then the volume was measured again (Vs). The swelling ratio was calculated using the following equation: Swelling ratio (%) = [(*Vs*–*Vi*)/*Vi*] ×100, where *Vs* is the volume of the swollen sample and *Vi* is the initial volume of the sample.

### ThT and Congo red staining

The cationic fluorescent dyes ThT and Congo red were used to investigate the amyloid structure of the solidified glue. The solidified proteins were immersed in an aqueous solution of dyes (100 μM) for 15 min in a dark environment. The photograph of ThT-stained solidified glue was taken under ultraviolet lamp irradiation.

### Tensile tests

The sample for tensile tests was prepared by pouring glue into a water-soluble container and waiting for 4 h until the glue completely solidified. The solidified glue at the bottom of the container was peeled off and cut into dumbbell-shaped blocks measuring 4 cm in length, 1 cm in width, and 0. 1 cm in thickness. The two ends of the sample were then fixed onto the fixture of a universal material testing machine with a 100 N load cell. (UTM2103, Shenzhen Suns Technology STOCK Co., Ltd.). The stress–elongation curve was recorded at 10 mm/min at room temperature. The tensile strength was obtained from the failure point, and the elastic modulus was determined by the average slope over 0–20% of elongation from the stress–elongation curve.

### Adhesion tests

#### Substrate preparation

All the substrates to be tested, including steel, aluminum, titanium alloy, ceramics, glass, nylon 66, PVC, PMMA, PP, bone, and pigskin, were cut into 1 cm × 5 cm strips, and a small hole is opened at one end of the strip for fixation during tensile testing. No further treatment was carried out on any of the substrates.

#### Underwater adhesion experiment

Firstly, a glass crystallizing dish with a diameter of 180 mm was filled with two-thirds of the total volume of tap water to it. Then, placed the substrate flat in the bottom of the container and used a pipette to drop 10 μL of protein glue on the end of the substrate away from the hole. Quickly covered it with another piece of substrate with an overlap area of 1 cm × 1 cm and clamped the two substrates to prevent slipping before the glue solidifies. After 24 h, the two substrates will be firmly bonded and the clamp was removed.

#### Lap shear adhesion

The bonded substrate was transferred into a custom-made transparent box filled with water to ensure that the entire test is carried out underwater. The adhesion strength was measured by a universal material machine with a 100 N load cell (UTM2103, Shenzhen Suns Technology STOCK Co., Ltd.). During the measuring, the holders and samples were immersed in water. The motorized part was moved along the vertical (shear adhesion test) axis with a retraction rate of 1 mm/min until the adhesive interface separated. The adhesion strength of the glue was determined by dividing the maximum force by the overlapping area. Each condition was tested with at least three samples.

### Mussel adhesion test

Mussels (*Perna viridis*, 30–40 g, ~6 month) were fished from the sea area of Rongcheng, Shandong Province by cutting the byssus. Immediately, the Mussels were transfer to a thermostatic water tank with local seawater under 22 °C. The water tank was transported to Xi’an by vehicle. They were fed with *Chlorella* once a day. After the experiment finished, they were released to their home sea area after the study.

The original byssus of the mussel was cutoff along the shell and then placed on a glass sheet in a seawater-filled aquarium. After 2 weeks, the newly settled mussels secreted new byssus and adhered to the glass. Then, the mature byssus of the mussel was cutoff, and the glass with many byssus was removed. The adhesion force between the glass sheet and the mussel byssus was measured by a universal material tester. The adhesive strength was determined by dividing the maximum force by the adhesive area of the mussel foot counted by ImageJ.

To investigate the resistance of mussel byssus to alkaline solution, the samples were soaked in an alkaline solution at pH = 12 for 48 h. The BSA glue adhesive on glass slides was also treated under the same conditions.

### DMA

The sample for DMA tests was prepared by pouring adhesive into the water and waiting for 4 h until the adhesive completely solidified. The solidified adhesive was then peeled off from the bottom of the container and cut into a rectangular block measuring 1 cm in length, 1 cm in width, and 0.1 cm in thickness. DMA850 equipment (TA Instruments). Frequency-sweeps were performed in the range of 0.1–200 Hz to the sample at room temperature with a strain amplitude of 0.05%. Master curves of storage modulus G′, loss modulus G′′, were recorded.

### Far-UV CD

Far-UV CD spectra were collected by using a Chirascan spectrophotometer (Applied Photophysics, Ltd., England). In detail, the unfolded BSA was diluted to a concentration of 0.1 mg/mL by an 80% TFE aqueous solution, and the CD spectrum was recorded from 190 nm to 260 nm with a bandwidth of 2.0 nm. Native BSA at the same concentration was also measured.

### XRD measurements

The freeze-dried protein glue samples were carried on X-ray diffractometer (Bruker D8 Advance, Cu target, wavelength = 0.154 nm), the scanning rate was set as 1 degree/min.

### TEM

The solidified glue block was obtained by vacuum freeze-drying to retain its original structures as much as possible. Then, the block was immobilized by using epoxy embedding medium (Epon_812 substitute, Sigma-Aldrich), and 70-nm sections were cut with an ultramicrotome (Leica EM UC7). The sections were examined with a FEI Tecnai G2 F20 Plus TEM.

### SEM

Field emission scanning electron microscopy (FE-SEM) observations were conducted on an SU-8020 (Hitachi, Japan). The solidified glue was freeze-dried, and the cross-section with gold sputtering was then observed after fracturing the samples.

### ToF-SIMS analysis

The specimens for ToF-SIMS analysis were prepared as that of XPS. ToF-SIMS data were acquired on an ION-ToF ToF. SIMS 5 instrument (ION_ToF, Germany) using a Bi^3+^ primary ion source with an electric current of 1 pA. The corresponding spectra were obtained from an area of 100 μm × 100 μm, and the positive ion spectra of each sample were collected 20 times.

### Wound closure

All animal experiments were approved by the Animal Ethical Committee of the Shaanxi Normal University (no. 2020209). All rates were housed in an experimental animal room maintained on a 12:12-hour, light:dark cycle. The temperature and humidity were maintained ~20 °C and between 45–55% respectively. In order to assess the wet wound closure and biocompatibility of the unfolded protein glue, 15 male SD rats (250−300 g, 6–8 weeks old) were randomly divided into three groups (*n* = 5 in each group) consisting of a control group, suture group, and glue group. Considering our animal experiment, however, only requires cutting the skin of the mouse, so rats are not required to be fully anesthetized. Therefore, rats were kept semi-conscious by injecting pentobarbital sodium as well as lidocaine intraperitoneally for local anesthesia, which also reduced the toxic effects of excessive doses of pentobarbital sodium, such as respiratory depression and low blood pressure^53^. Specifically, pentobarbital sodium (30 mg/kg) was injected intraperitoneally to induce anesthesia, and then the exposed skin was washed with saline and disinfected with iodophor. Prior to operation, lidocaine solution (1% w/v) was smeared on the depilation area. 2 min later, the back surface of the rats was longitudinally cut (2 cm). Control group wounds were not treated, while suture group wounds were sutured with catgut. For the experimental group, 50 mL of urea-stabilized BSA glue was smeared on the incision, and then 2 mL of normal saline was applied quickly to solidify the glue. Pictures were taken on the 0th and 3rd day to see how the wound was healing. On days 3 and 7 after CO_2_ euthanasia, the skin near the wounds was harvested. The collected skin (3 × 1 cm) was utilized for both mechanical strength measurements and histological analysis. To measure the wound-breaking strength of the rejoined tissue, the skin was loaded on the universal testing machine with a crosshead speed of 5 mm/min. For histological analysis via hematoxylin and eosin (H&E) staining, the remaining skin section was fixed in 10% buffered formalin (Sigma). To confirm collagen synthesis, Masson’s trichrome (MT) staining was performed. All imaging analyses were performed on a Leica microscope. The significance of differences between results was assessed via two-sided Tukey’s multiple comparisons test.

### Patching rubber pipe

Our glue (50 μL) was applied on a rounded rubber sheet (the diameter was 2 cm) and pressed tightly on the hole (5 mm) of a leaking rubber pipe under a water pressure of 100 kPa.

### Repairing the accidental puncture of inflatable boat

An inflatable boat was punctured with a broken hole (2 mm) in the swimming pool. The unfolded BSA glue (100 μL) was applied on a rounded rubber sheet (the diameter was 3 cm) and pressed tightly on the hole. After ~70 s later, the repaired inflatable boat had recovered normal rowing.

### Fixing the sand in the trough

A pinch of sand (~200 g) was placed in the middle of a sloping trough and injected unfolded BSA glue (3 mL) into it. Then washing it with water (the average flow is 20 mL/s) to see if the sand can be fixed without being washed away.

### Bonding to a glass container

Five pieces of 5 × 5 cm glass sheets were coated with our glue on the edges, formed into a cube, and then injected with water to solidify it. Thus, a glass box was obtained after 30 min.

### Closure of bladder foramen

A fresh pig bladder with a 5 mm hole was prepared first. The unfolded BSA glue (~100 μL) was coated on the hole and washed with copious amounts of saline to solidify the glue. Two hours later, saline was injected into the bladder with a maximum pressure of 60 cm H_2_O.

### MD simulations

All the MD simulations in this work were performed in the NPT ensemble using the Gromacs 5.1.5 package with the forcefield of Gromos96 (53a6)^54^. The visualization was generated via Chimera 1.14^55^. All the bonds with hydrogen atoms were constrained by the LINCS algorithm^56^. The cutoff for the nonbonded van der Waals interaction and the long-range electrostatic interaction were set as 1.0 nm. The experimental temperature was maintained at 310 K with V-rescale, and the pressure was kept at 1 bar using Parrinello-Rahman. The simple point charge water model was used as the solvent. Each system was performed with a time step of 2 fs. The secondary structure changes were carried out by the do_dssp tool in the Gromacs package. Insulin was selected as a model molecule because of its small molecular size to reduce the consumption of computer resources.

Four systems were investigated. (i) The unfolding of insulin. An octamer of insulin with all disulfide bonds cleaved was dissolved in the TEF-filled box and simulated by the NPT ensemble for 200 ns. Insulin with intact disulfide bonds was used as a control. The change in root-mean-square deviation and the distance among every peptide were measured. (ii) The coacervation of unfolded insulin. The unfolded insulin chains prepared by removing the TFE molecules were put into a water-filled box. (iii) The interaction between the unfolded protein and the surface. The unfolded insulin was placed above the silica or PP slide at a distance of 1 nm. Water molecules were added to the system and simulated for 300 ns. The binding free energy of each residue was calculated by the MMPBSA algorithm^57^. The initial contact time of each residue to the surface of silica and PP was recorded. The contact time was defined as the moment that the minimum distance between the residue and interface was less than 0.35 nm (the diameter of water molecules). (iv) Adhesion of unfolded protein between two layers of silica. The unfolded insulin chains were placed between two layers of silica followed by adding water into the system. To simulate the adhesion process, the system was conducted for 600 ns by an NPT ensemble. The obtained structure of an unfolded insulin-bonded silica slide was placed into a box with a size of 10 × 10 × 20 nm. After pre-equilibration, a steered molecular dynamic simulation was conducted to analyze the tensile stress. In detail, one silica slide was fixed, and another silica slide was dragged by a constant force of 1000 kJ/mol/nm along the *y* axis direction. The shear adhesion strength (*τ*) can be calculated by Eq. 1

1 $$\tau=\frac{F}{A}$$

where *F* is the tensile breaking force and the *A* is the area of the silica slide.

### Numerical study

When BSA glue was placed in a water bath, diffusion interchange occurred between the two phases. The diffusion process of water molecules can be described by Fick’s second law. BSA glue underwent phase transformation and solidification when encountering water. Therefore, we have to consider two states: one is the liquid state, and the other is the solid state after solidification. Water diffusion coefficients in these two states are different, and the relationship of the mass transfer process needs to be expressed by a piecewise function

2 $$\frac{\partial c}{\partial t}=\left\{\begin{array}{c}{D}_{1}\cdot {\nabla }^{2}c,\,c\, < \, {C}_{c}\\ {D}_{2}\cdot {\nabla }^{2}c,\,c\ge {C}_{c}\end{array}\right.$$

where $t$ is the diffusion time, ${D}_{1}$ is the diffusion coefficient of water in the liquid BSA glue, ${D}_{2}$ is the diffusion coefficient of water in the solidified glue, and ${C}_{c}$ is the critical concentration of water that leads to glue curing.

To understand the above equation, a FE method-based solver, COMSOL Multiphysics, was used.

### Reporting summary

Further information on research design is available in the Nature Portfolio Reporting Summary linked to this article.

### Supplementary information

Supplementary Information

Peer Review File

Description of Additional Supplementary Files

Supplementary Movie 1

Supplementary Movie 2

Supplementary Movie 3

Supplementary Movie 4

Supplementary Movie 5

Supplementary Movie 6

Supplementary Movie 7

Supplementary Movie 8

Supplementary Movie 9

Supplementary Movie 10

Reporting Summary

### Source data

Source Data

## Data Availability

The data supporting the findings from this study are available within the Article, Supplementary Information, or Source Data file. Source data are provided with this paper.
