# Supplementary material for: Synthesis of robust underwater glues from common proteins via unfolding-aggregating strategy
Source: Nat Commun. 2023 Aug 24;14:5145. doi: 10.1038/s41467-023-40856-z (PMC10449925; doi:10.1038/s41467-023-40856-z)
Supplement: Supplementary file 1 — Supplementary Information [file 41467_2023_40856_MOESM1_ESM.pdf]

Supplementary Information

Synthesis of robust underwater glues from common proteins via unfolding-aggregating strategy

Yongchun Liu[1#], Ke Li[2#], Juanhua Tian[3], Aiting Gao[1], Lihua Tian[1], Hao Su[1], Shuting Miao[1], Fei Tao[1], Hao Ren[1], Qingmin Yang[1], Jing Cao[4], Peng Yang[*1]

[1] Key Laboratory of Applied Surface and Colloid Chemistry, Ministry of Education, School of Chemistry and Chemical Engineering, Shaanxi Normal University, Xi'an 710119, China.

[2] Xi'an Key Laboratory for Prevention and Treatment of Common Aging Diseases, Translational and Research Centre for Prevention and Therapy of Chronic Disease, Institute of Basic and Translational Medicine, Xi'an Medical University, Xi'an, 710021, China.

[3] Department of Urology, The Second Affiliated Hospital of Xi'an Jiaotong University, Xi'an 710004, China.

[4] Key Laboratory of Archaeological Exploration and Cultural Heritage Conservation Technology, Ministry of Education, Institute of Culture and Heritage, Northwestern Polytechnical University, Xi' an 710072, China.

Email: yangpeng@snnu.edu.cn

#These authors contribute equally.

1 Supplementary method

1.1 Materials

Bovine serum albumin (BSA), lysozyme, thioflavin-T (ThT), N-(1-pyrenyl) maleimide (NPM), and agarose were purchased from Sigma-Aldrich. Other proteins, cysteine and glutathione (GSH) were used as received from Shanghai Yuanye Bio-Technology Co., Ltd. Tris(2-carboxyethyl)phosphine hydrochloride (TCEP), 2,2,2-trifluoroethanol (TFE), dimethyl formamide (DMF), 1,1,1,3,3,3-hexafluoro-2-propanol (HFIP) and other organic solvents were supplied by TCI. Guanidine hydrochloride (GuHCl) and urea were purchased from Aladdin. Ultrapure water was used in all experiments and was supplied by Milli-Q Advantage A10 (Millipore, USA).

1.2 Experimental section

NPM staining. NPM (300 μL, 10 mM) in DMF and TCEP (50 μL, 0.6 M) was added to a quartz cell, followed by the addition of BSA (2650 μL, 20 mg/mL) in an 80% TFE solution. The sample was then measured by a fluorescence spectrophotometer (excitation wavelength 330 nm, emission wavelength 380 nm) with a bandwidth of 5 nm. Native BSA at the same concentration was also analyzed for comparison.

FTIR measurements. To obtain the sample for FTIR measurement, the solidified protein glues were freeze-dried and mixed with dry KBr with a weight ratio of 0.5 ~ 1%. By using a Bruker Vertex 70v spectrometer, the FTIR spectra were obtain with the wavenumber from 400 to 4000 cm^{-1}.

Raman spectra. Aqueous solutions of unfolded BSA (50 μL, 200 mg/mL) and native BSA (50 μL, 200 mg/mL) were dropped onto a glass slide and dried at room temperature. Then, the Raman spectra were analyzed with a Renishaw Raman inVia Reflex spectrometer (diode laser wavelength of 785 nm).

Cytotoxicity test. A CCK-8 assay was used to determine the in vitro cytotoxicity of unfolded BSA glue. Cell lines of hepatocellular carcinomas (HepG2, HUH-7) and normal cells (HL-7702, HEK293, HT22) were cultured in 96-well plates with 12000 cells per well, and then mixed with different concentrations of glue (243, 81, 27, 90 μg/mL). After removing the old culture medium, 10% CCK-8 was added to the colorless medium. Upon incubation for 2 hours, the absorbance of each well was measured at 450 nm. The cell survival rate of each well was calculated based on each well in the control group.

Double staining with Calcein/PI was used to evaluate the cytotoxicity of unfolded BSA glue on Hippocampal Neuronal Cell Line (HT22). Calcein/PI dye was added to the medium after washing it twice with PBS to observe whether the cells survive or die using a fluorescence microscope.

Conogenic assay. Cell suspensions were prepared by digesting HEK293, HL-7702, HepG2, HUH-7 cells with trypsin. And then, these cells were inoculated in a 6 cm dish with a concentration of 2000 cells/dish. A gradient of glue concentrations of 200 ug/mL, 100 ug/mL, and 50 ug/mL was mixed into the cells. Culture was continued until the 7th day, and cell state was monitored. After cloning, the cells were fixed with 4% paraformaldehyde and stained with crystal violet dye solution. In the end, we counted the number of clones in each dish.

Hemolysis test. We prepared 2% red blood cell suspension in normal saline. A glue solution with a final concentration of 2 mg/mL was mixed into the cell suspension, while 1% triton X-100 solution and normal saline were used as controls. The samples were incubated at 37 °C for 2 hours, and then centrifuged for 3 minutes at 9425 g. The absorbance of supernatants at 545 nm was measured, and triton X-100 group was used as a reference for 100% hemolysis.

Acute toxicity test. All animal experiments were approved by the Animal Ethical Committee of the Shaanxi normal University (No. 2020209). All mice were housed in experimental animal room maintained on a 12:12-hour, light:dark cycle. The temperature and humidity were maintained ~20 °C and between 45-55% respectively. Sixteen healthy female BALB/c mice (~20 g, 6-8 weeks old) were divided into two groups, half males and half females in each, and given intravenous diluted glue (2 mg/mL) and normal saline, respectively. During the experimental period, each mouse was injected with 200 uL of sample, and he or she was prohibited from eating or drinking for six hours before and four hours after injection. We recorded the physical symptoms of mice, including intraocular hypertension, messy hair, difficult breathing, convulsions, aphasia, and coma. For histopathological analysis, all mice were killed and the internal organs (heart, liver, spleen, lung and kidney) of each group of mice were removed, embedded in paraffin, and sectioned.

In vivo degradability

For monitoring the degradation of unfolded BSA glue, Cy5.5-labeled BSA was mixed into the urea-stabilized unfolded BSA glue with a final concentration of 0.1% (w/w%). In order to obtain the solidified glue brick, glue was added to water. In order to prepare the body implanted into rats, we cut solidified glue brick into cuboid shapes measuring 5×5×1mm (wet weight: 0.0269 g, dry weight: 0.0028 g).

All animal experiments were approved by the Animal Ethical Committee of the Shaanxi normal University (No. 2020209). All rates were housed in experimental animal room maintained on a 12:12-hour, light:dark cycle. The temperature and humidity were maintained ~20 °C and between 45-55% respectively. During the surgery, 20 male SD rats (5-6 weeks old) weighing 180 - 200g were kept in the SPF animal room for a week to adapt to the environment. Then, their backs were depilated, and the isoflurane-anesthetized rats were fixed on the cork platform. After disinfecting the back, a scalpel was used to cut open the skin and bluntly separate it. After implanting the glue sample 1 cm into the incision, the wound was sutured and disinfected. Lidocaine is then dripped into the wound for analgesia, and surgical wound glue is then applied to close the incision. Local analgesia was maintained for 48 hours after the wound was examined and disinfected for three consecutive days. In the following seven days, the wound must be examined and nursed as necessary. After that, 4 SD rats were selected every 10 days to observe the implanted sample. In detail, after gas anesthesia, the fluorescence was observed and photographed in vivo by using the small animal living imaging system (Visque invivo Smart-LF, Vieworks, South Korea). Detail parameters are Cy5.5 excitation and emission, exposure time 2 s, visual field 12.5 × 12.5 cm, and floor temperature 37 °C. Following deep anesthesia, skin tissue with residual samples was collected. The tissue samples were then characterized by histological staining with H&E and immunohistochemistry with CD68. Furthermore, blood biochemistry analysis was performed on whole blood and serum.

Bonding versatile powder-like materials to bulk material. One gram of glue composed of inexpensive soy protein (50% urea and 100 mg/mL soy protein) was mixed with 9 g of powder-like materials, including microorganisms (e.g., chlorella and yeasts), pine sawdust, corn stalk powder, cellulose powder, shrimp shell powder, and chitosan powder. After sufficient stirring, these mixtures were put into a mold and treated by hot compacting (30 MPa, 80 °C) for 30 min.

Determination of the water diffusion coefficient. The diffusion coefficient of water was measured by Fourier transform infrared-attenuated total reflection (FTIR-ATR) spectroscopy. This technique has increasingly been used to study absorption kinetics in polymer systems and has proven to be accurate and reliable.[1] **Supplementary Fig. 1** shows the schematic of the experiment. BSA glue or solidified glue was cast onto the surface of the ATR prism and then immersed in pure water. The penetration depth of the IR beam in the sample can be calculated by Equation 1.

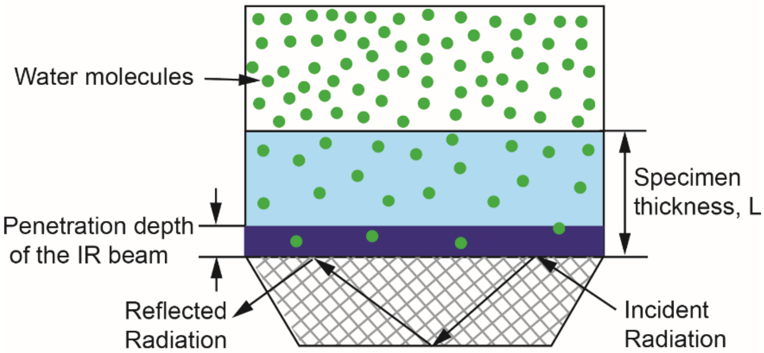

Supplementary Fig. 1 Schematic of the experimental setup for measuring the diffusion coefficient of water.

$$dp = \frac{\lambda}{2\pi({n_1}^2 sin^2\theta - {n_2}^2)^{1/2}} \qquad (1)$$

where dp is the depth of penetration of the evanescent wave, λ is the wavelength of the infrared beam, n_1 and n_2 are the refractive indices of the flat crystals and protein solutions, respectively, and θ is the incident angle of the beam.

According to the Beer-Lambert law (Equation 2), the peak intensity is related to the water concentration c,

$$A = abc \qquad (2)$$

where a is the absorption of the component at a measured frequency and b is the pathlength of the component.

As a result, the Fickian concentration profile can be developed by Equation 3 while $A_t/A_\infty \geq 0.5$ (Fieldson, Barbari, 1993).

$$\frac{A_t}{A_\infty} = 1 - \frac{8\gamma}{\pi[1-\exp(-2\gamma l)]} \times \left[\frac{\exp(\frac{-D\pi^2 t}{4l^2})\left(\frac{\pi}{2l}\exp(-2\gamma l)+(2\gamma)\right)}{\left(\frac{\pi^2}{4l^2}+4\gamma^2\right)}\right] \quad (3)$$

where $\gamma = \frac{1}{dp}$ and l is a half of the film thickness.

Although the value of γ depends on the refractive index of the protein solution, it can be simplified as a constant if it meets the following condition

$$4\gamma^2 \gg \frac{\pi^2}{4l^2} \text{ and } 1 \gg \exp(-2\gamma l) \quad (4)$$

Therefore, Equation 3 can be rewritten as

$$\ln\left(1 - \frac{At}{A_\infty}\right) = \ln\left(\frac{4}{\pi}\right) - \frac{D\pi^2}{4l^2}t \quad (5)$$

As shown in **Supplementary Fig 1**, a tube open at both ends was fixed on ATR prism, and the lip was sealed with Vaseline. BSA glue or solidified glue was added to the tube, followed by the addition of pure water. The characteristic peak of water in the region between 3800 cm^{-1} and 2750 cm^{-1} was measured by a Vetex 70v (Bruck), and the diffusion coefficient of water was calculated based on Equation 5.

X-ray photoelectron spectroscopy (XPS). Silica wafers cleaned with piranha solution and a PTFE sheet were bonded together with BSA glue. After 4 hours, the PTFE sheet was removed from the silica wafers. The solidified glue remained almost completely on the wafer because the adhesion to the wafer is much greater than that of PTFE. Then, XPS was performed with an AXIS ULTRA from Kratos Analytical, Ltd.

1.3 Theoretical calculation section

Numerical study of the solidification of the protein glue.

The model of the BSA glue solidification process is shown in **Supplementary Fig 2**. In detail, the solidification process of BSA glue between two round glass slides in water was calculated. Because the glass slide is centrally symmetrical, only a 1/4 cylinder needs to be calculated. The parameters used in COMSOL are listed in **Supplementary Tab. 1.**

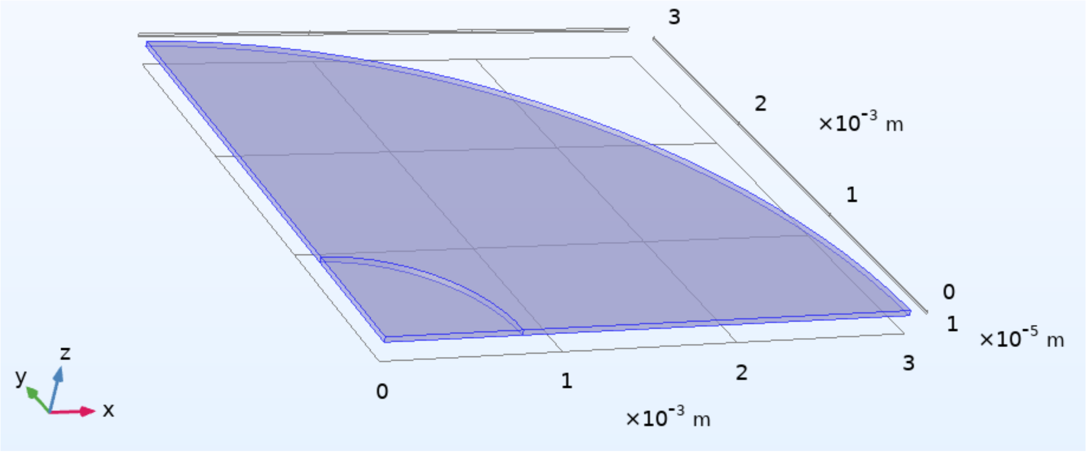

Supplementary Fig. 2 The model for finite element calculation.

2. Supplementary Figures

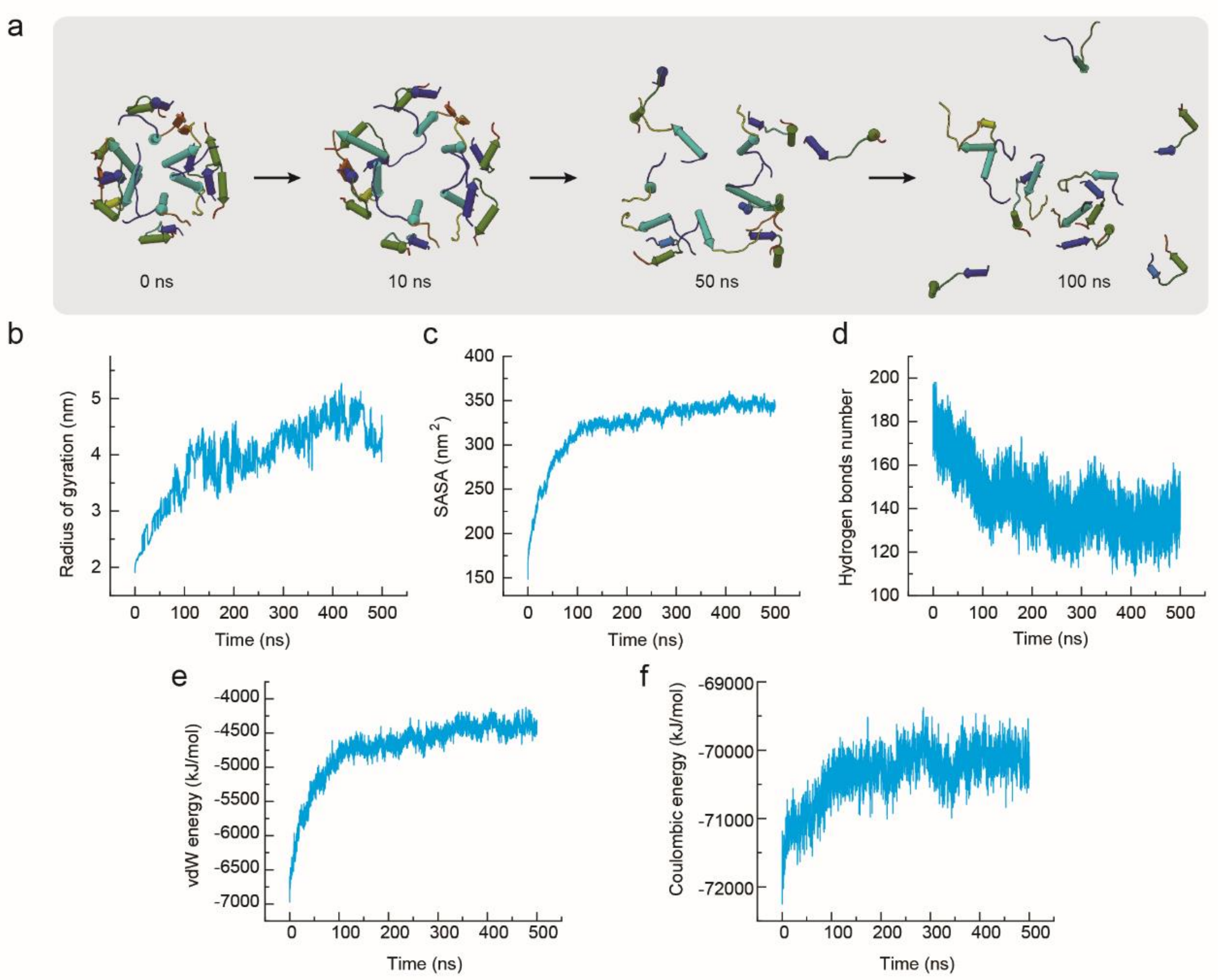

Supplementary Fig. 3 MD simulation results of S-S bond-reduced insulin in TFE. **a** Snapshots of the MD indicating the insulin unfolded in TFE. The radius of gyration (Rg) (**b**) and the solvent accessible surface area (SASA) (**c**) change of S-S bond-reduced insulin during the 500 ns of simulation. The increase in these two factors indicates that the existence of TFE could make insulin unfold. The development of hydrogen bond number (**d**), van der Waals (vdW) energy (**e**), and coulombic energy (**f**) between the polypeptide chains of insulin in TFE after its S-S bonds were reduced. The decrease in hydrogen bond number and the increase in vdW and coulombic energy suggest that the TFE surrounding the polypeptide chain can shield the nonbonded intermolecular interaction.

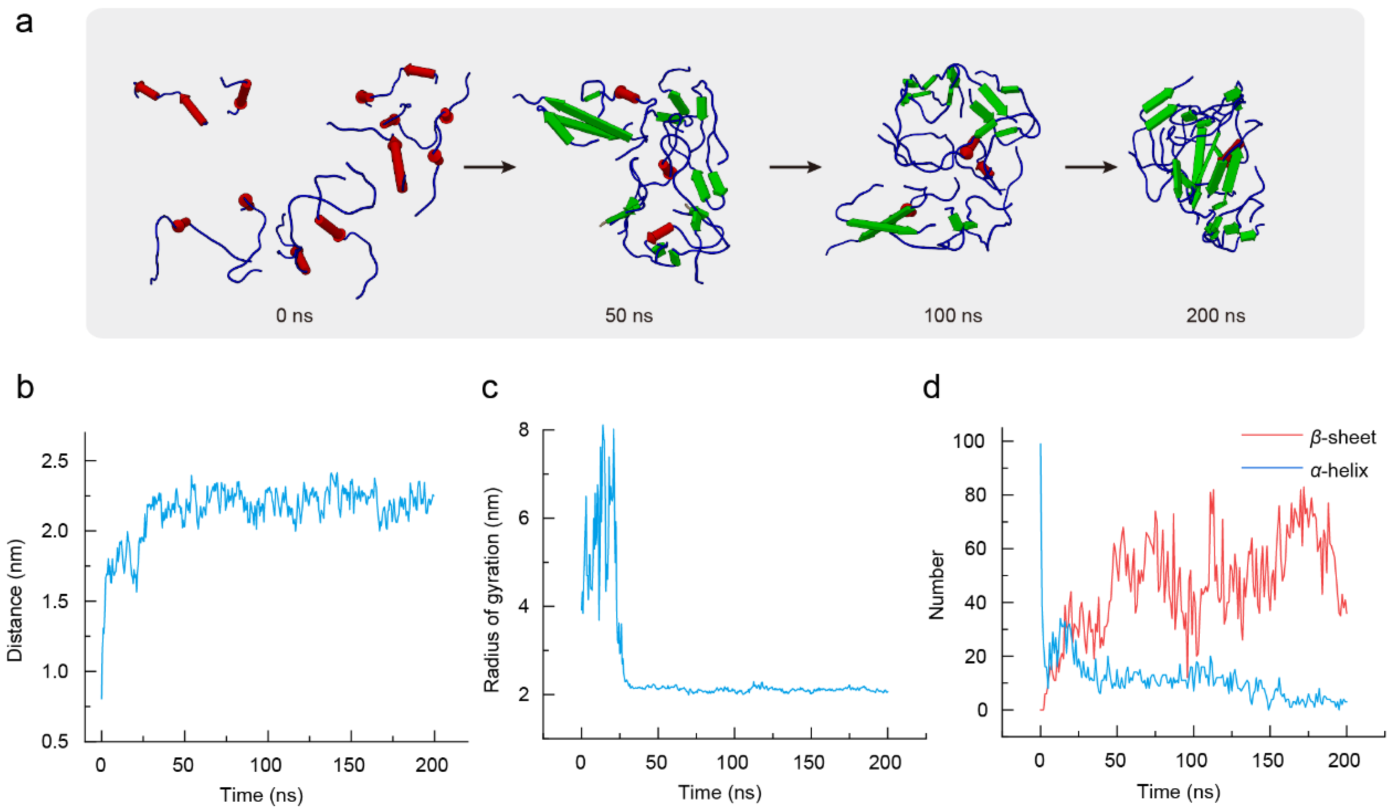

Supplementary Fig. 4 a Snapshots of the aggregation process of the unfolded insulin chains. The corresponding evolution of the distance between TFE and insulin (**b**), the radius of gyration (**c**) and the secondary structure change (**d**) of the protein during the aggregation process. The distance between TFE and insulin shows an obvious increase, indicating that TFE was removed from the surface of unfolded insulin.

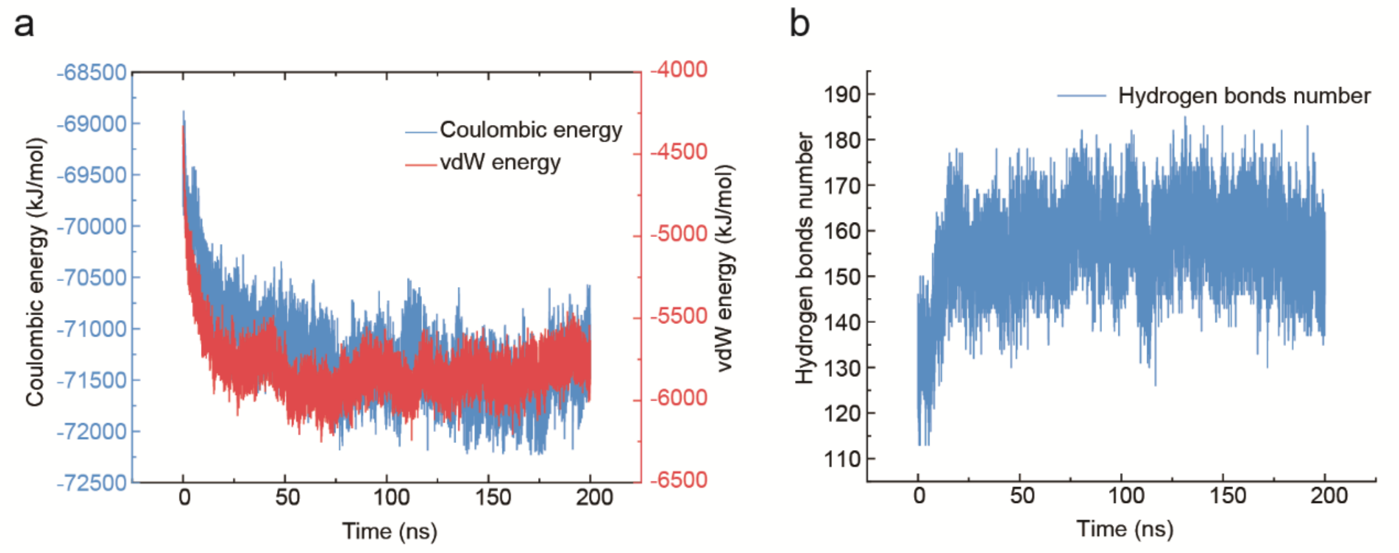

Supplementary Fig. 5 The interaction among the unfolded insulin chains during aggregating process. The development of van der Waals (vdW) energy, coulombic energy (**a**) hydrogen bond number (**b**), between the polypeptide chains of unfolded insulin in water.

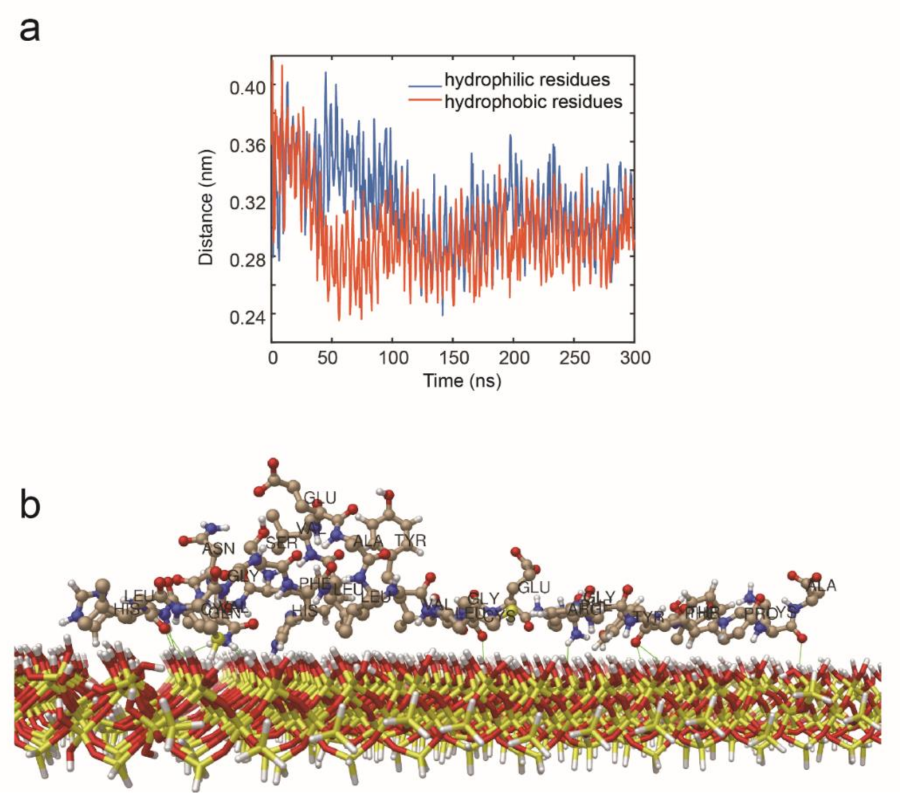

Supplementary Fig. 6 a Distance change between each residue and the silica sheet. **b** The interaction between each residue of the insulin and the silica sheet. The yellow lines stand for hydrogen bonds.

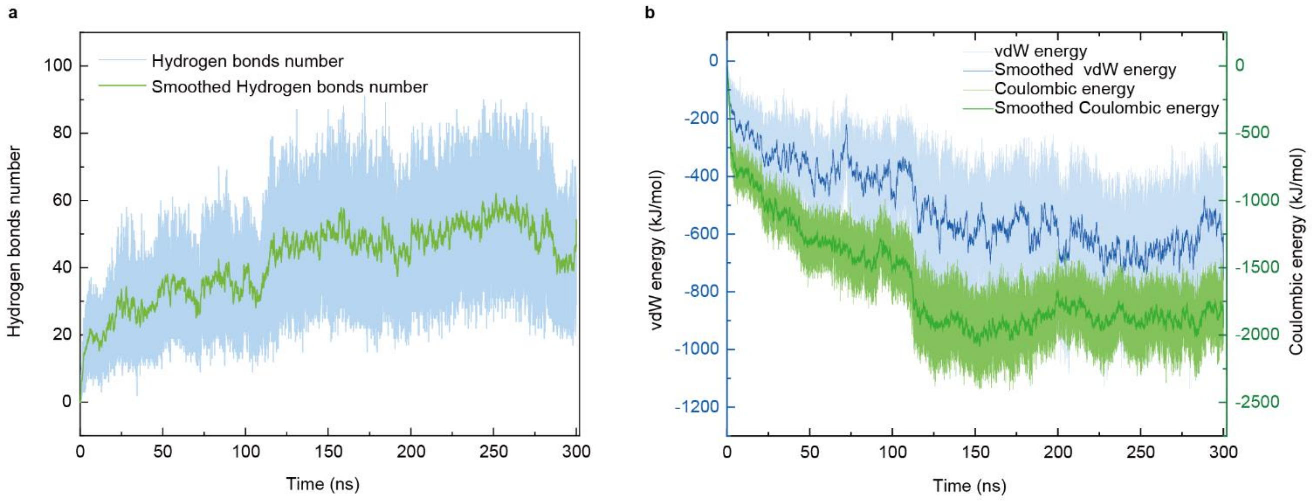

Supplementary Fig. 7 The hydrogen bond number (**a**) as well as electrostatic and van der Waals (vdW) interactions (**b**) change during the interaction of unfolded insulin with the silica surface. The growing hydrogen bond number as well as decreasing electrostatic and hydrophobic (vdW) interaction energy indicated that these interactions play a key role in the adhesion of proteins on the surface.

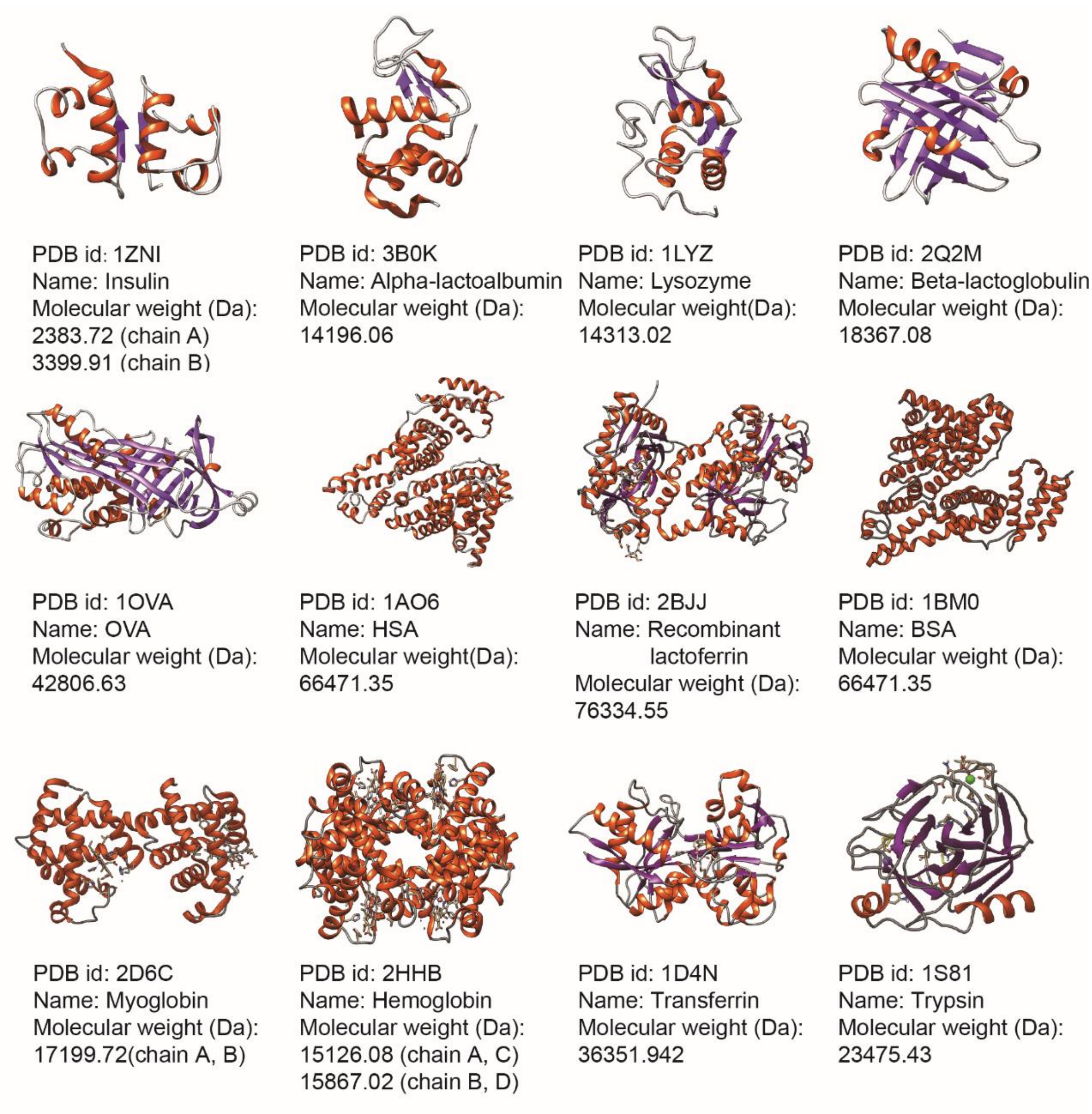

Supplementary Fig. 8 The molecular structure and molecular weight of different proteins used in this work.

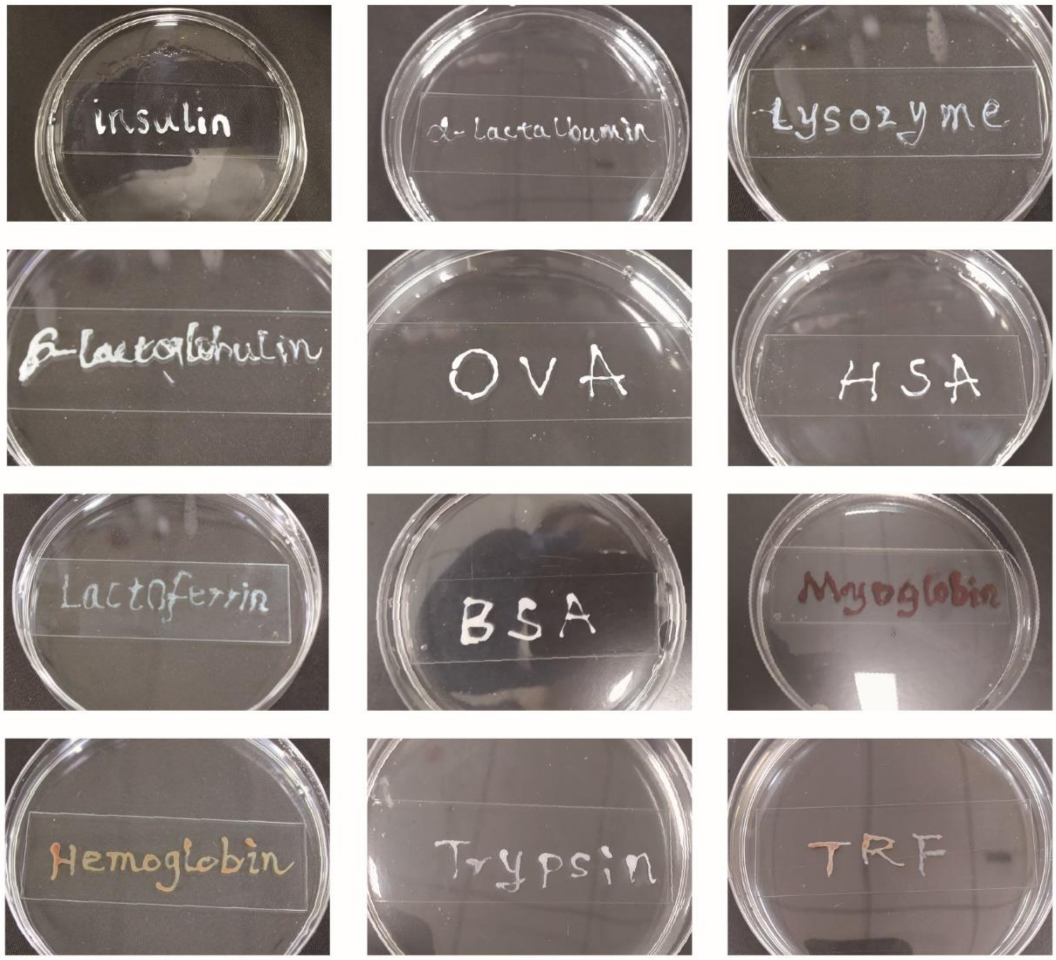

Supplementary Fig. 9 The words written by different unfolded protein samples in water, including insulin, α-lactalbumin, lysozyme, β-lactoglobulin, OVA, HSA, lactoferrin, BSA, myoglobin, hemoglobin, trypsin and transferrin (TRF). These word patten still remained mostly after being rinsed with tap water for 30 seconds, indicating that these glues have strong interface adhesion capacity.

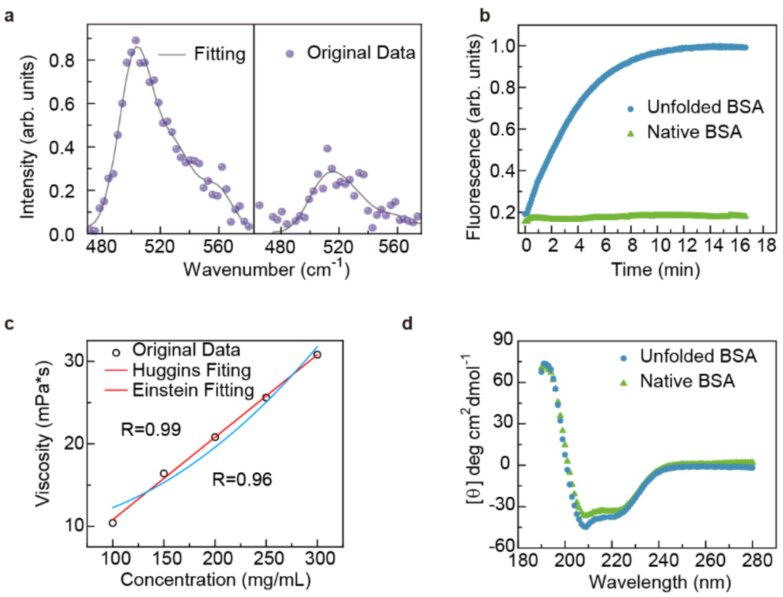

Supplementary Fig. 10 a Raman spectra of native BSA and unfolded BSA. The peak between 480 ~ 570 cm^{-1} belongs to the S-S bond of the protein. **b** The fluorescence of the NPM assay with native BSA and unfolded BSA. **c** Concentration dependence of the viscosity change of native BSA aqueous solution. It shows a better fitness with the Huggins equation (R^2 = 0.99) than with the Einstein equation (R^2 = 0.96), indicating that the native BSA is spherical. **d** CD spectra of native and unfolded BSA. In the unfolded protein solution, a stronger feature of α-helical structure was obvious, which was ascribed to the trifluoroethanol-induced stabilization of the α-helical structure.[2]

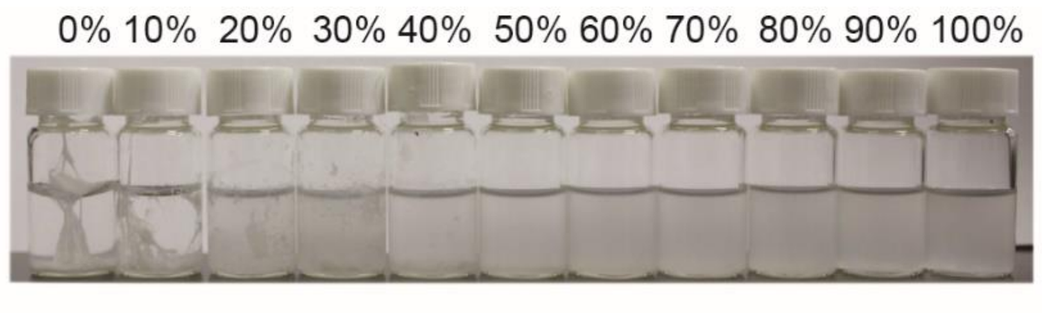

Supplementary Fig. 11 Photographs of the unfolded BSA glue in TFE solutions with different TFE concentrations.

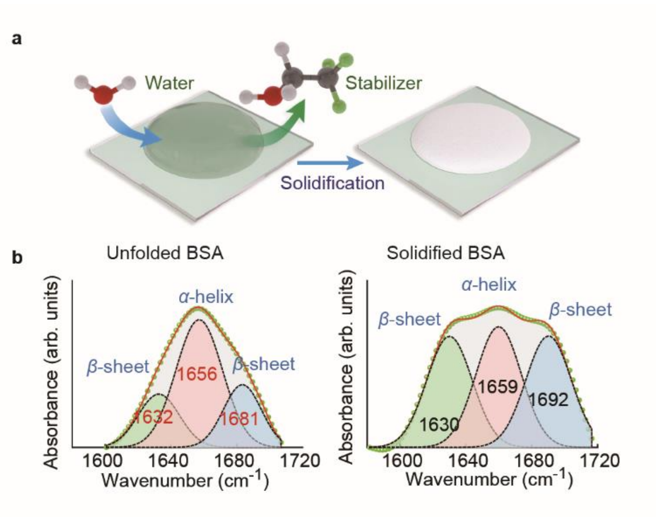

Supplementary Fig. 12 a Schematics of the solidification of unfolded BSA in water. **b** The deconvolution of amide I from 1600 to 1720 cm^{-1} for unfolded BSA and solidified BSA.

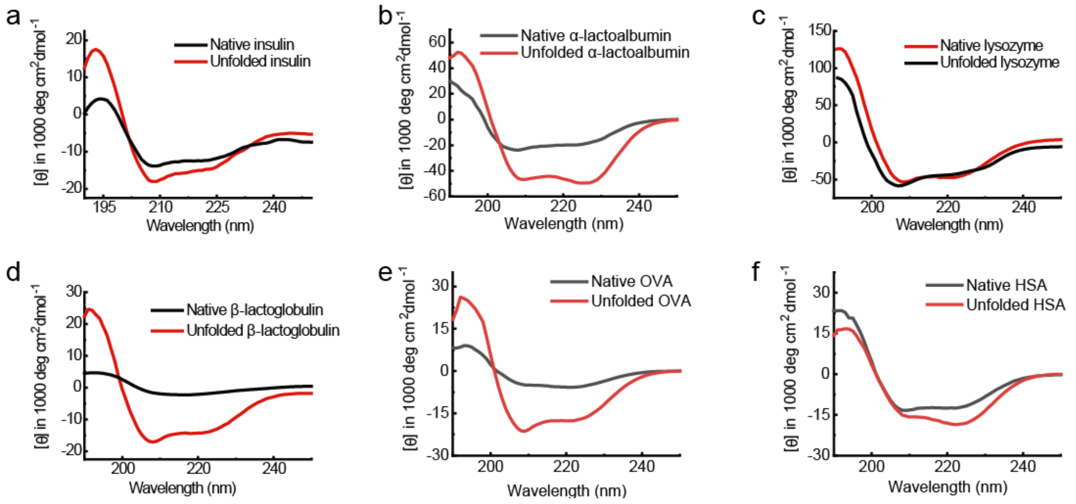

Supplementary Fig. 13 CD spectra of native and unfolded insulin (**a**), native and unfolded α-lactalbumin (**b**), native and unfolded lysozyme (**c**), native and unfolded β-lactoglobulin (**d**), native and unfolded OVA (**e**), and native and unfolded HSA (f).

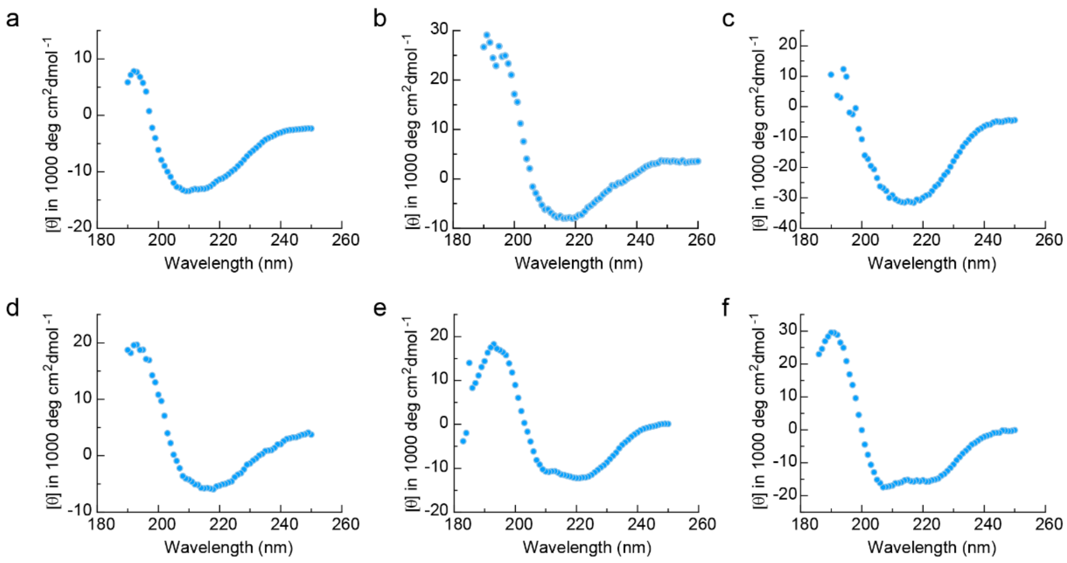

Supplementary Fig. 14 CD spectra of solidified unfolded proteins including insulin (**a**), α-lactalbumin (**b**), lysozyme (**c**), β-lactoglobulin (**d**), OVA (**e**) and HSA (**f**).

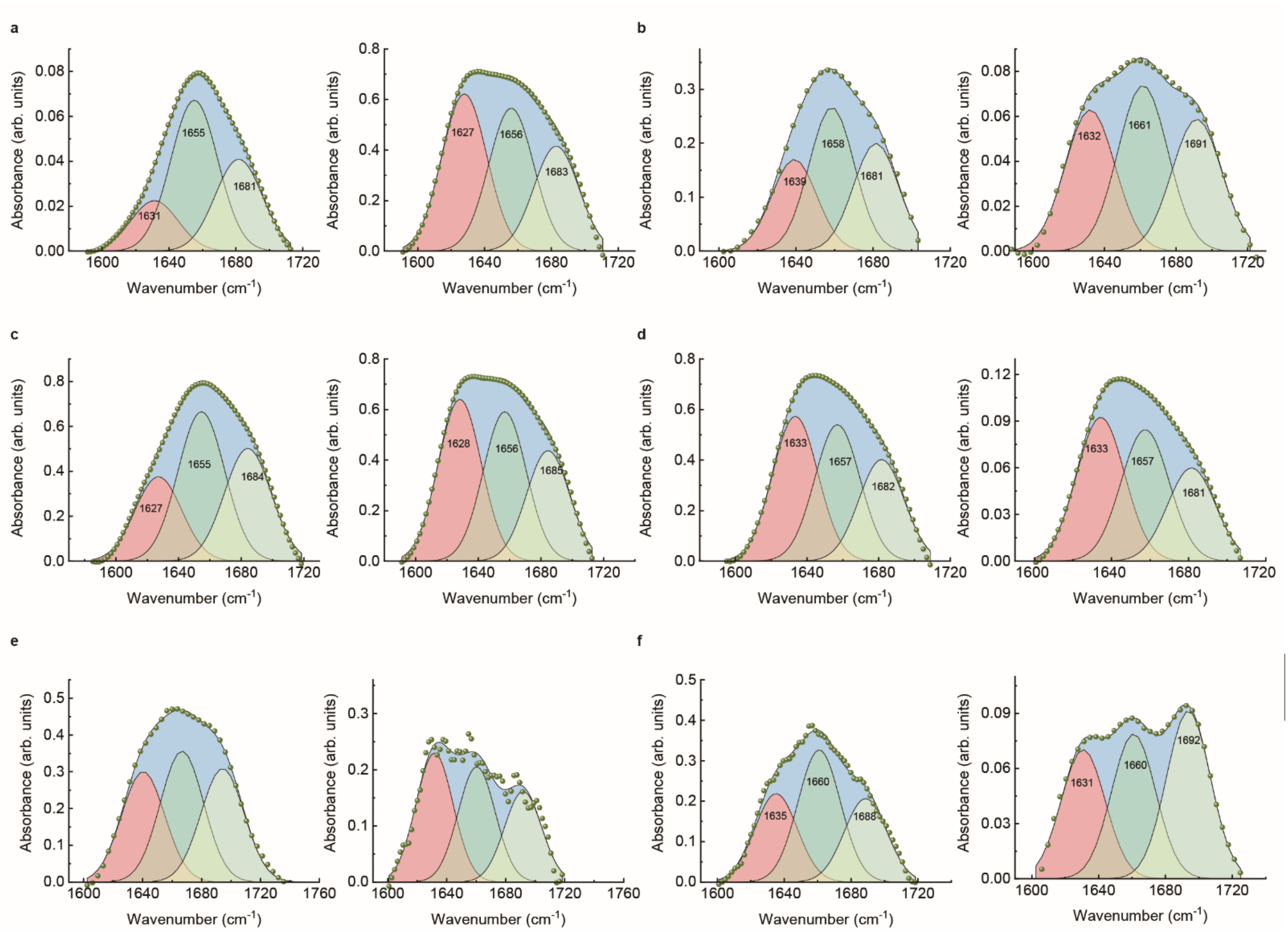

Supplementary Fig. 15 FTIR spectra of amide I of native and solidified unfolded proteins including insulin (**a**), *α*-lactalbumin (**b**), lysozyme (**c**), *β*-lactoglobulin (**d**), OVA (**e**) and human serum albumin (HSA) (**f**). In (**a-f**), the left panel is for native protein, and the right panel is for the solidified unfolded protein sample.

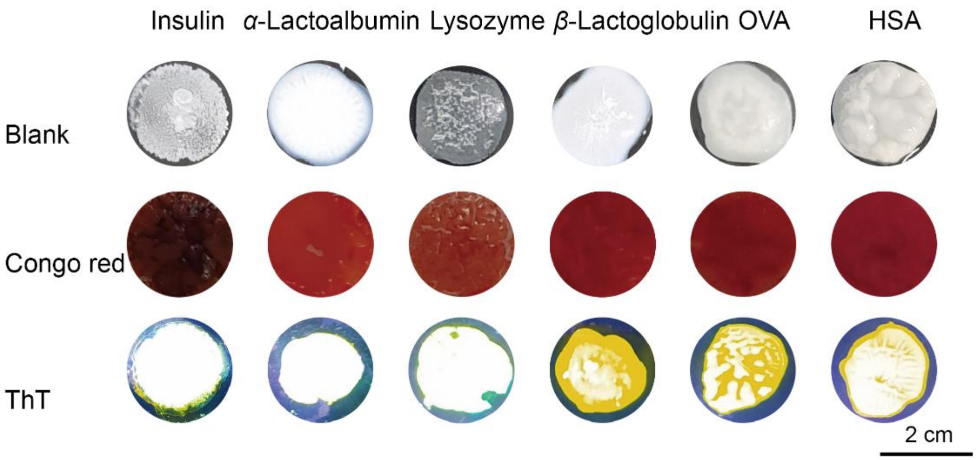

Supplementary Fig. 16 Photographs of the solidified unfolded protein glue after ThT and Congo red staining.

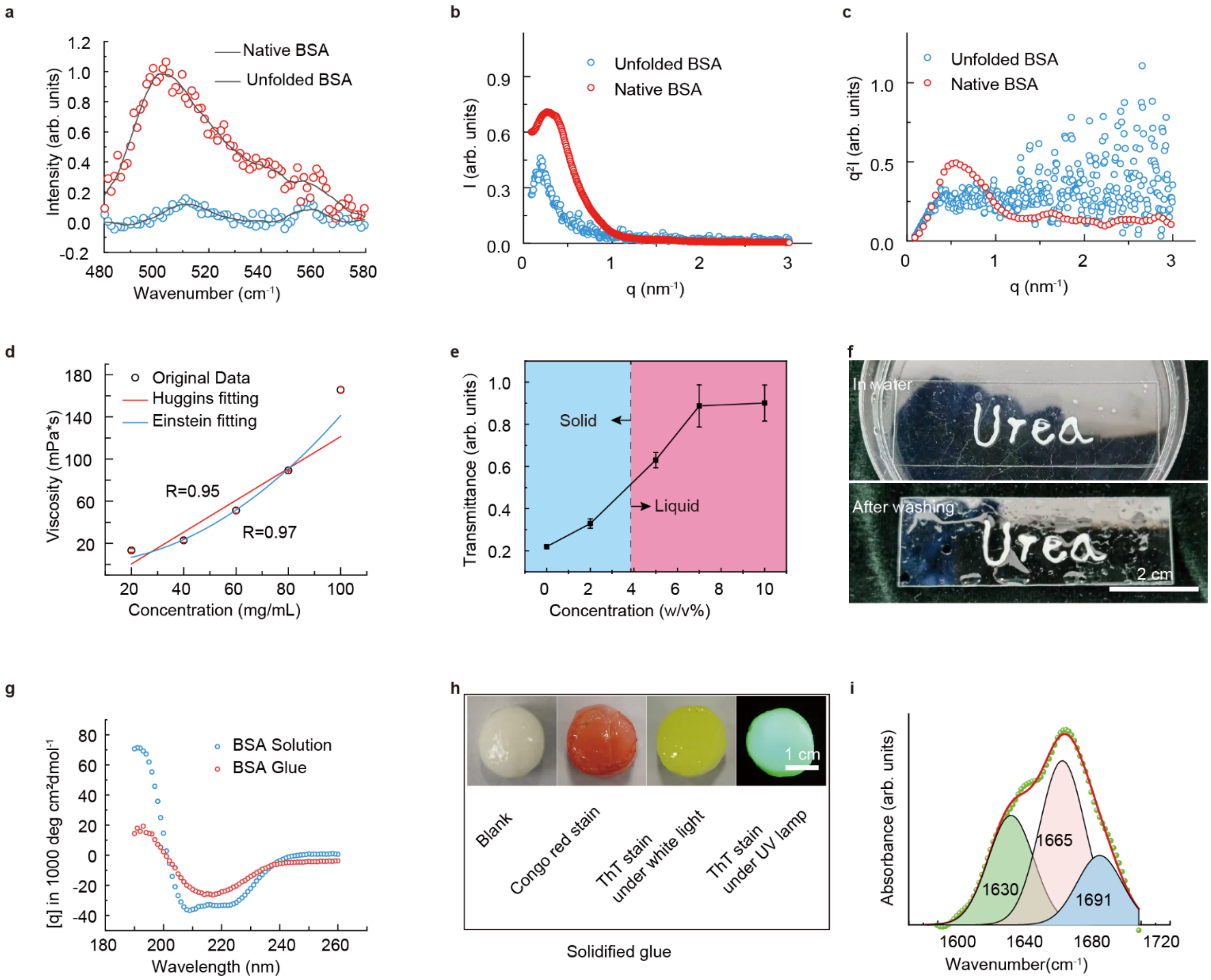

Supplementary Fig. 17 The unfolding and aggregation of urea/cysteine system-treated BSA. **a** Raman spectra of native BSA and unfolded BSA. The peak between 480 ~ 580 cm^{-1} belongs to the S-S bond of the protein. Scattering plots (I ~ q) (**b**) and corresponding Kratky plots (I·q2 ~ q) (**c**) of native and unfolded BSA. I, scattering intensity; q, scattering vector. **d** Concentration dependence of the viscosity change of unfolded BSA solution. It shows a better fitness with the Einstein equation (R^2 = 0.97) than with the Huggins equation (R^2 = 0.95), indicating that the BSA in this system is unfolded. **e** Light transmittance (wavelength = 600 nm) of unfolded BSA solution with urea added at different concentration. Data are mean ± S.D. n = 3 independent samples per group. **f** The word "Urea" written on an underwater glass slide by using the unfolded BSA glue and it can withstand strong rinsing with tap water from the faucet. **g** CD spectra of the unfolded BSA and its aggregation triggered by water. **h** Photograph of ThT and Congo red staining of the solidified unfolded BSA glue. **i** The deconvolution of amide I from 1600 to 1720 cm^{-1} for solidified BSA.

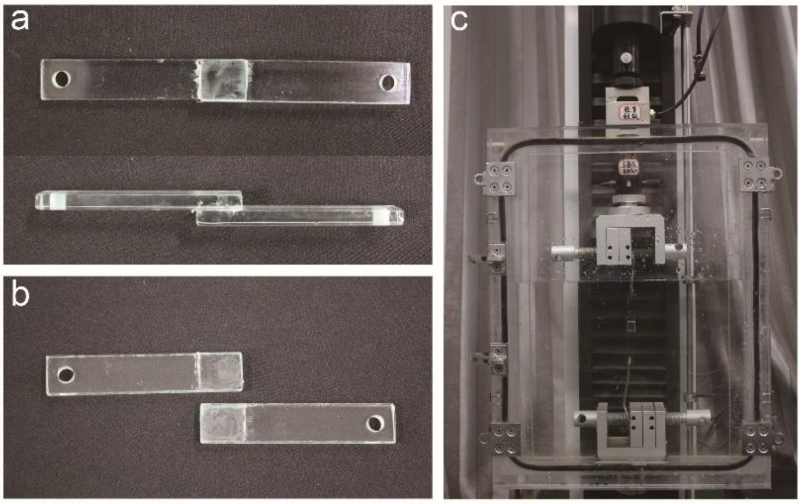

Supplementary Fig. 18 Bonded glass slides (**a** and **b**) and adhesion strength testing equipment (**c**). Overlapping area is 1 cm^2.

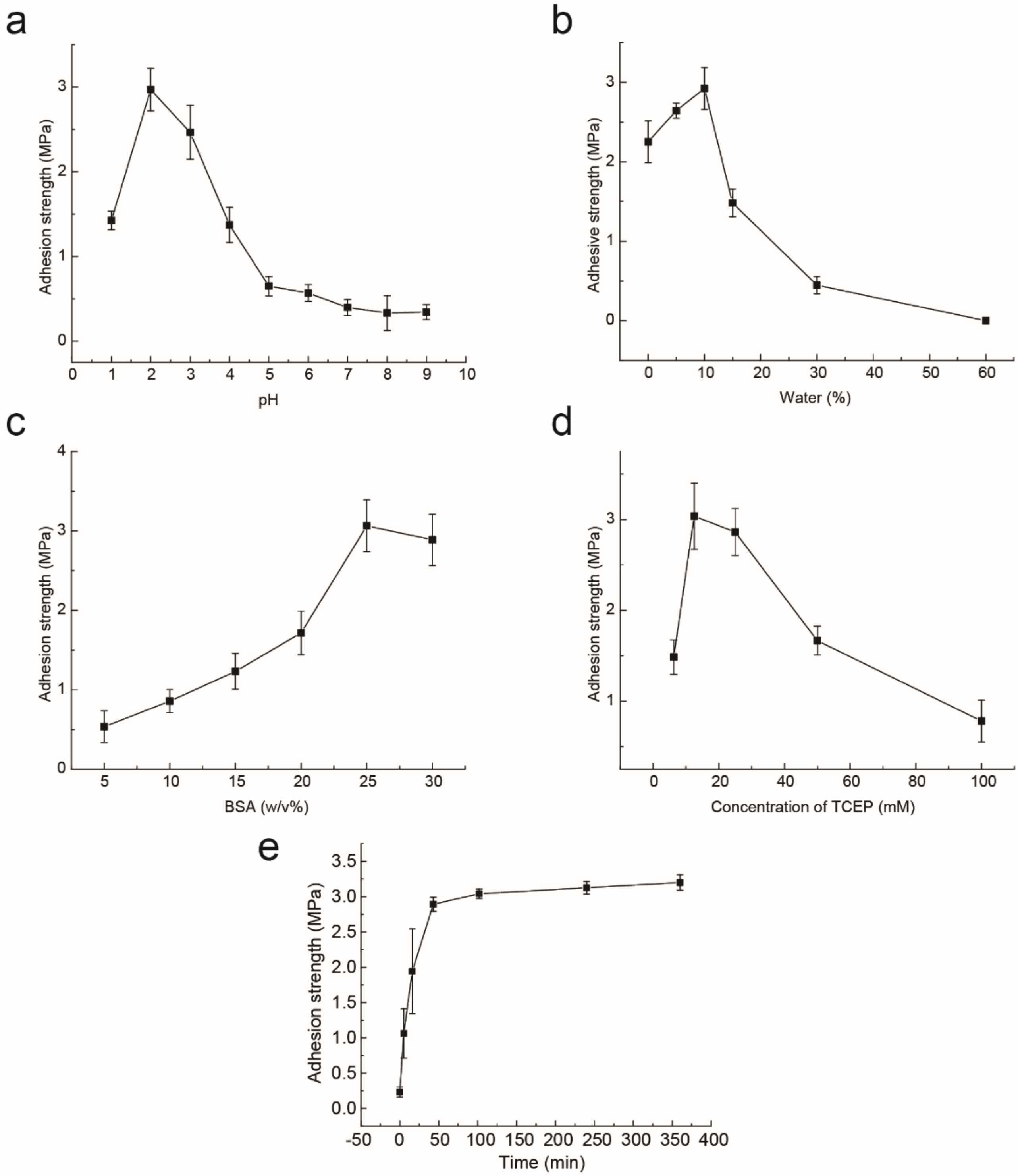

Supplementary Fig. 19 Adhesion strength of unfolded BSA glue prepared under different conditions: (**a**) pH of the solution, (**b**) volume ratio of water, (**c**) BSA and (**d**) TCEP concentration in the solution and (**e**) reaction time after adding reducer and stabilizer. The optimization condition for preparing BSA glue is thus obtained, that is, pH 2, 80% TFE, 25% BSA, 20 mM TCEP and 20 minutes of reaction time. All data are mean ± S.D. n = 3 independent samples per group.

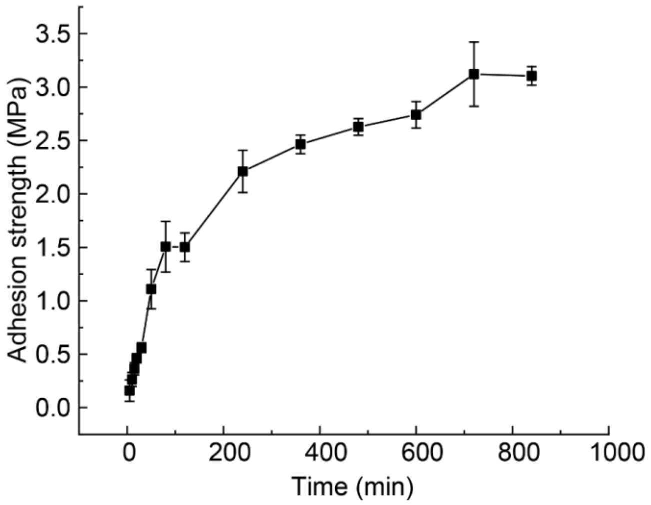

Supplementary Fig. 20 Adhesion strength of the unfolded BSA glue with different solidification time. Data are mean ± S.D. n = 3 independent samples per group.

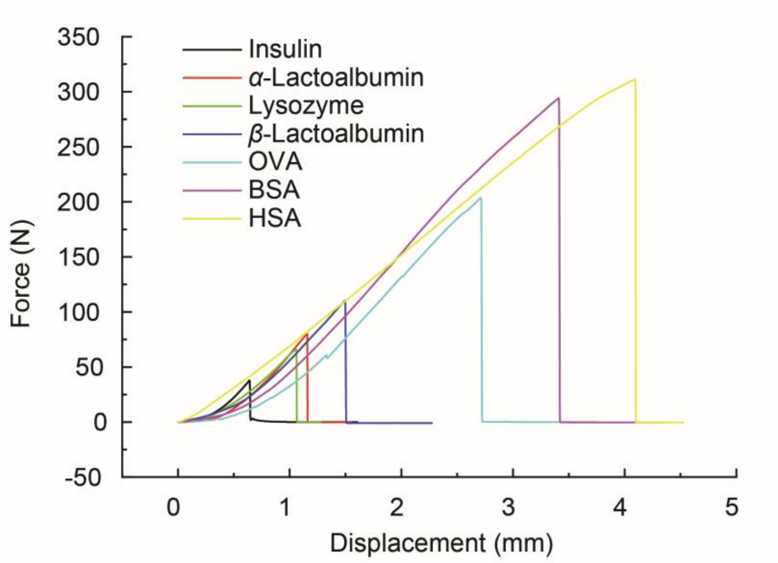

Supplementary Fig. 21 Displacement-force curves of glass slides bonded by different protein-based glues with an overlapping area of 1 cm^2.

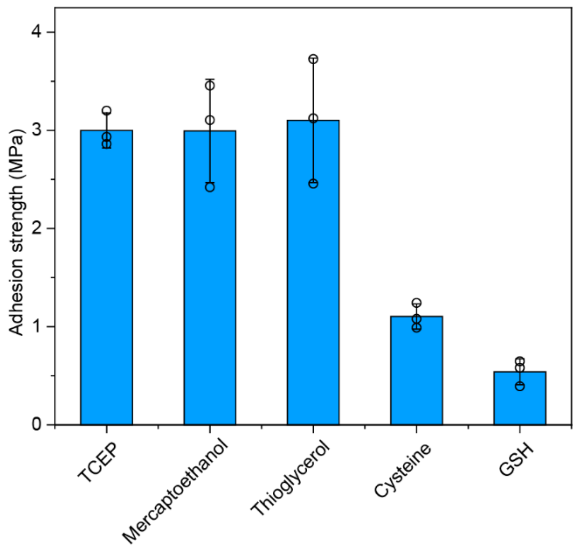

Supplementary Fig. 22 The adhesion strength of unfolded BSA prepared with different reductants. Data are mean ± S.D. n = 3 independent samples per group.

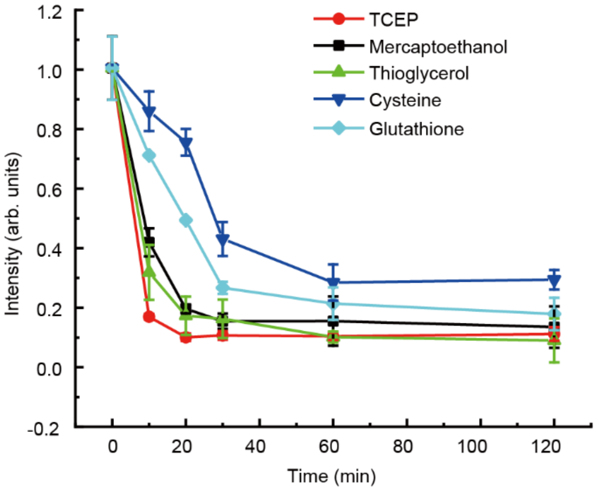

Supplementary Fig. 23 The time-dependent Raman spectra change of glues prepared with different reductants. The intensity of Raman spectra at 505 cm^{-1} (the characteristic peak of the disulfide bond) was measured and normalized to the magnitude of the symmetric ring breaking peak (1003 cm^{-1}). Data are mean ± S.D. n = 3 independent samples per group.

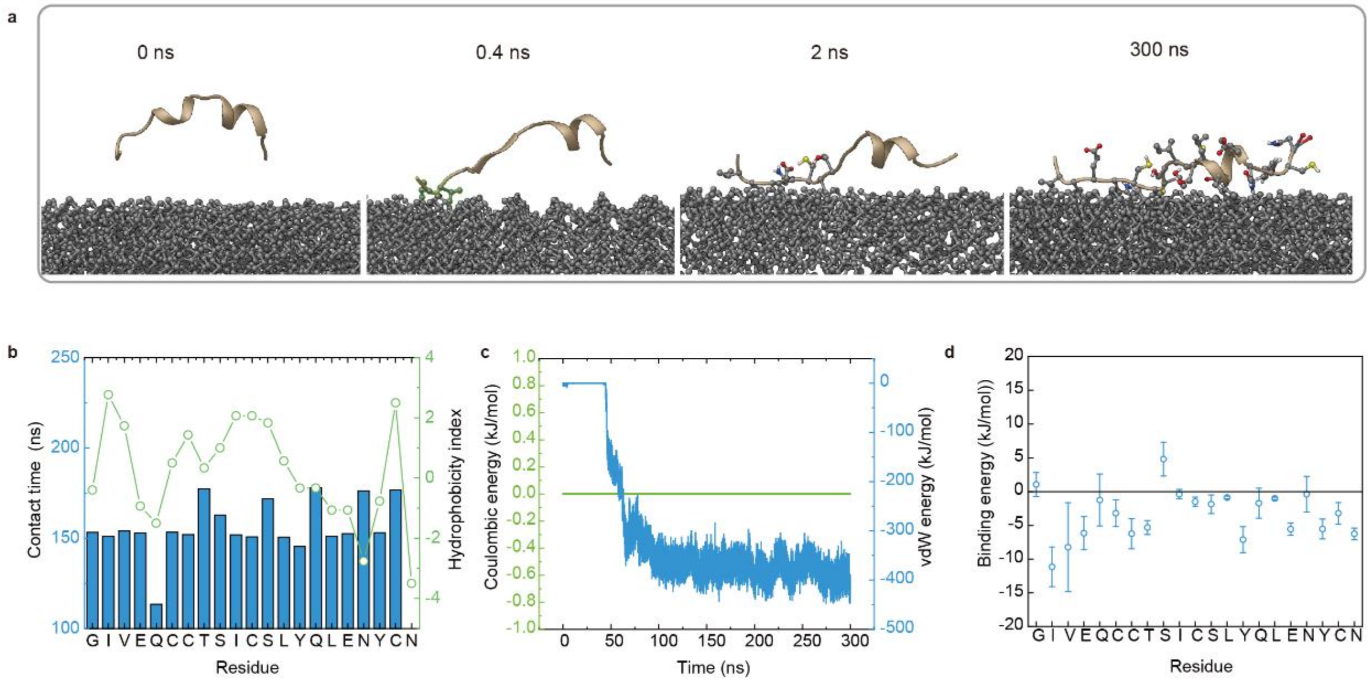

Supplementary Fig. 24 The interaction of insulin chain A with the PP surface by MD simulation. **a** Time-dependent snapshot of the insulin chain approaching the PP surface. **b** The initial contact time of insulin chain A interacting with PP. **c** The changes of electrostatic and vdW energy as well as hydrogen bond number during the interaction of insulin with the PP surface. **d** The binding energy of each residue after the whole protein chain adheres to PP. In contrast to the interaction between unfolded insulin and silica, the interaction between unfolded insulin and PP does not show any obvious selectivity towards hydrophilic and hydrophobic residues. Due to the hydrophobicity of PP, it may not produce significant electrostatic or hydrogen bond interactions except for hydrophobic interactions with unfolded insulin. Therefore, its adhesion strength is lower than that of hydrophilic materials. Data are mean ± S.D. n = 50 independent samples per group.

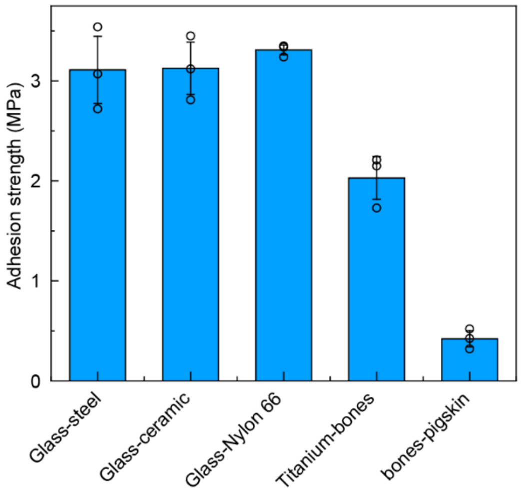

Supplementary Fig. 25 Adhesion strength of the unfolded BSA glue to bond different materials together. The adhesion strength between two different materials is near to the strength from the material side having a relative low adhesion strength with the glue. Data are mean ± S.D. n = 3 independent samples per group.

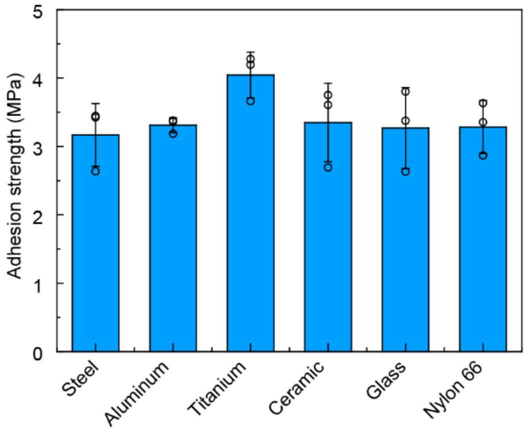

Supplementary Fig. 26 The performance of unfolded BSA glue to bond the same type of material together in a dry environment. Data are mean ± S.D. n = 3 independent samples per group.

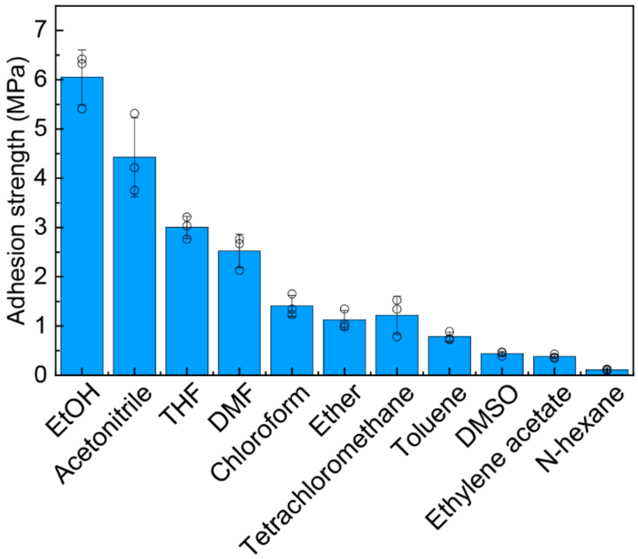

Supplementary Fig. 27 The performance of unfolded BSA glue in different organic solvent. Data are mean ± S.D. n = 3 independent samples per group.

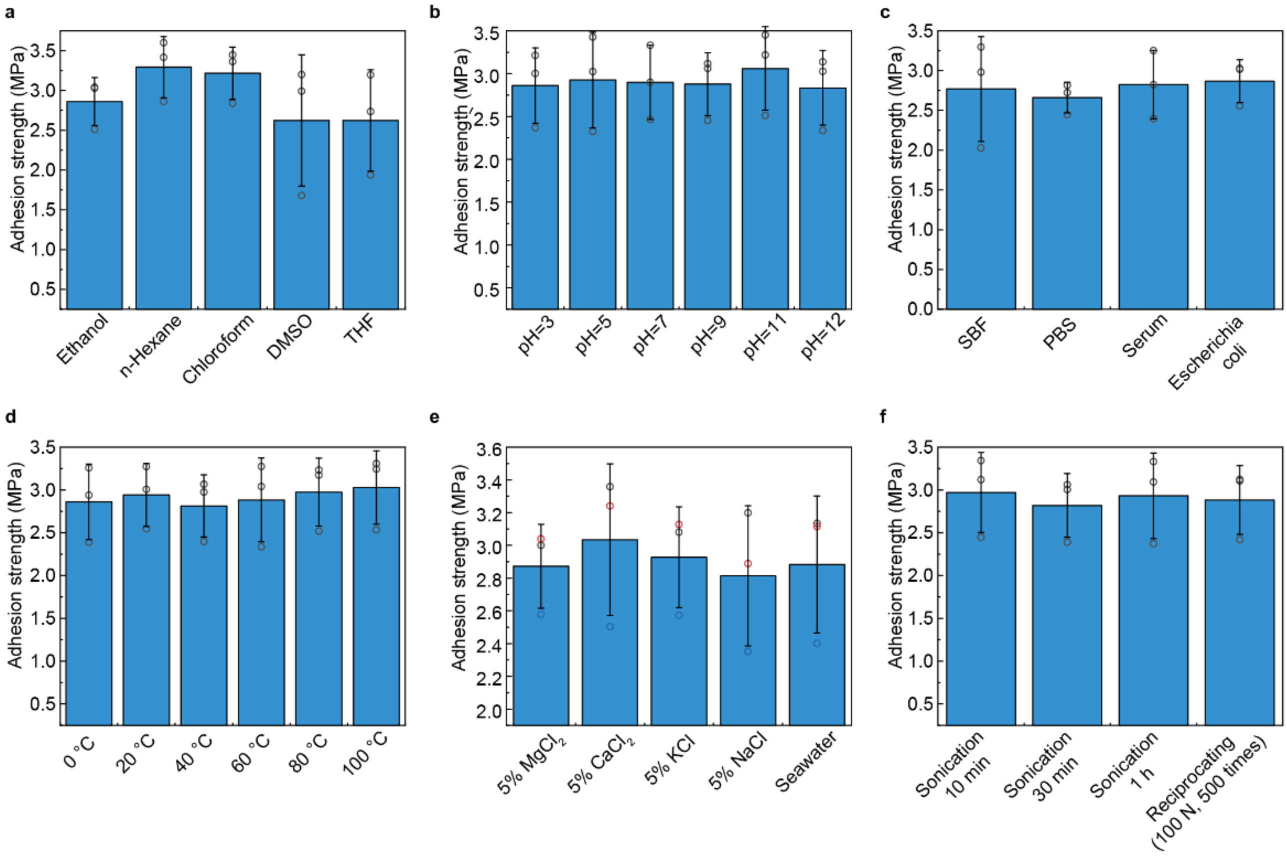

Supplementary Fig. 28 The stability of unfolded BSA glue under different conditions. The adhesive strength of BSA glue-bonded glass slides after immersion into different organic liquid including ethanol, n-hexane, chloroform dimethyl sulfoxide (DMSO) and tetrahydrofuran (THF) for 24 hours (**a**), aqueous solutions with different pH form 3 to 12 for 24 hours (**b**), some biological liquids such as simulated body fluid (SBF), PBS, fetal calf serum and the suspension of *E.coli* (ATCC8739) (3 months) (**c**), aqueous solutions with different temperatures (**d**), inorganic salt solution and seawater (7 days) (**e**), and ultrasonic processing for 10 min, 30 min and 1 hour, and the reciprocating for 500 times with the largest tension and stress setting as 100 N (**f**). All data are mean ± S.D. n = 3 independent samples per group.

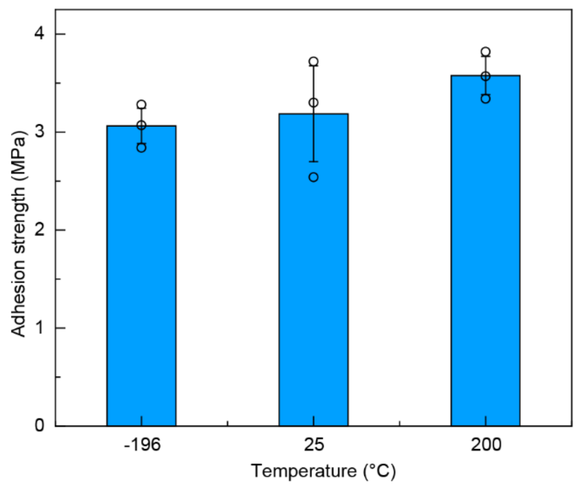

Supplementary Fig. 29 The adhesion stability of the unfolded BSA glue-bonded glass under different temperatures in dry environments. The adhesive strength of BSA glue-bonded glass slides after being treated by liquid nitrogen (-196 °C), room temperature or the oven (200 °C) for 24 hours. Data are mean ± S.D. n = 3 independent samples per group.

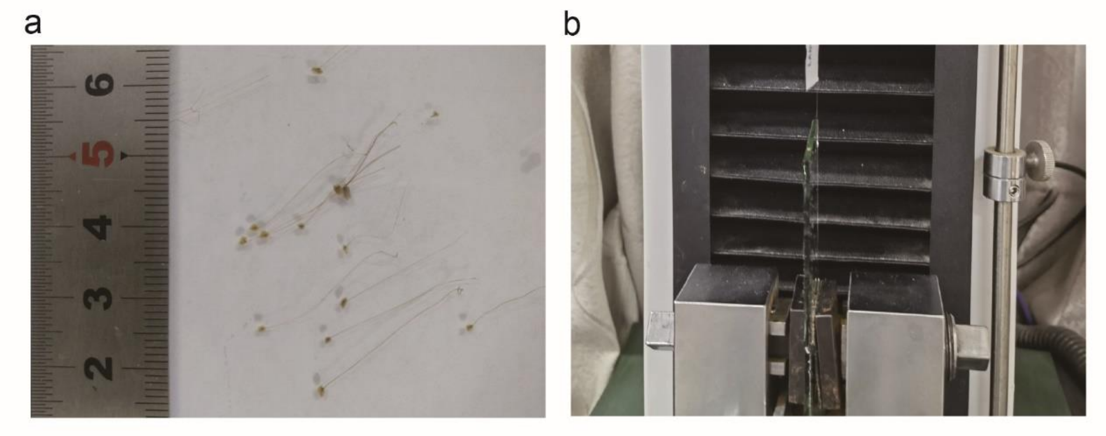

Supplementary Fig. 30 Photographs of mussels byssus bonded on glass (**a**) and the measurement of its adhesion strength (**b**).

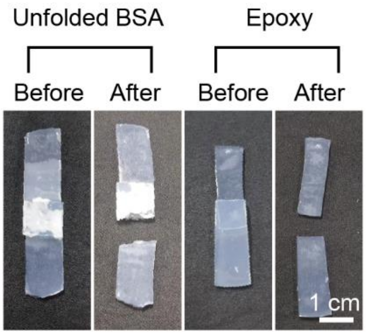

Supplementary Fig. 31 Comparing the adhesion of unfolded BSA glue with a commercial glue (epoxy) for 1% agarose gel.

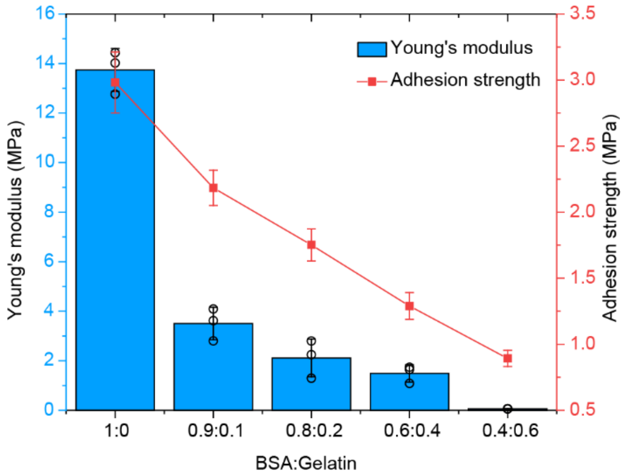

Supplementary Fig. 32 The modulus and adhesion strength of protein glue with different mass ratio of BSA to gelatin. The modulus and adhesive strength of the solidified glue decrease as the gelatin dosage rises. When the quantity of gelatin is increased to 60%, the underwater glues have an adhesion strength of approximately 0.9 MPa, and the modulus of the solidified can reach 73 kPa, which is more in line with the hardness of softer organs than the original composition. Data are mean ± S.D. n = 3 independent samples per group.

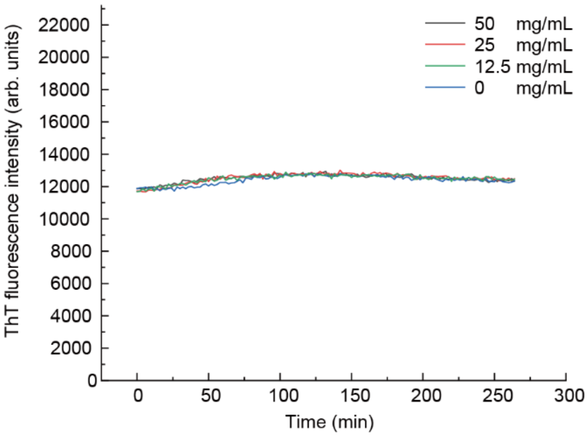

Supplementary Fig. 33 The ThT fluorescence of serum (10%) added with different concentration of BSA glue (urea/cysteine system). The ThT fluorescence does not rise, indicating that the glue will not cause amyloid deposits in blood.

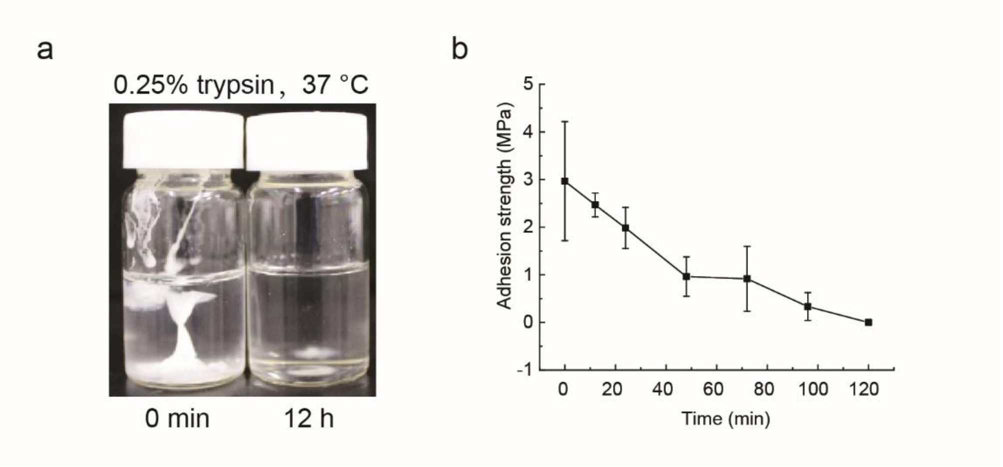

Supplementary Fig. 34 Trypsin hydrolyzed solidified glue. **a** Photograph of solidified glue immersed in 0.25% trypsin for 12 h. The glue bulk was completely hydrolyzed after 12 h. **b** Adhesion strength developing of BSA glue to glass slides after being treated by 0.25% trypsin solution at 37 °C. Data are mean ± S.D. n = 3 independent samples per group.

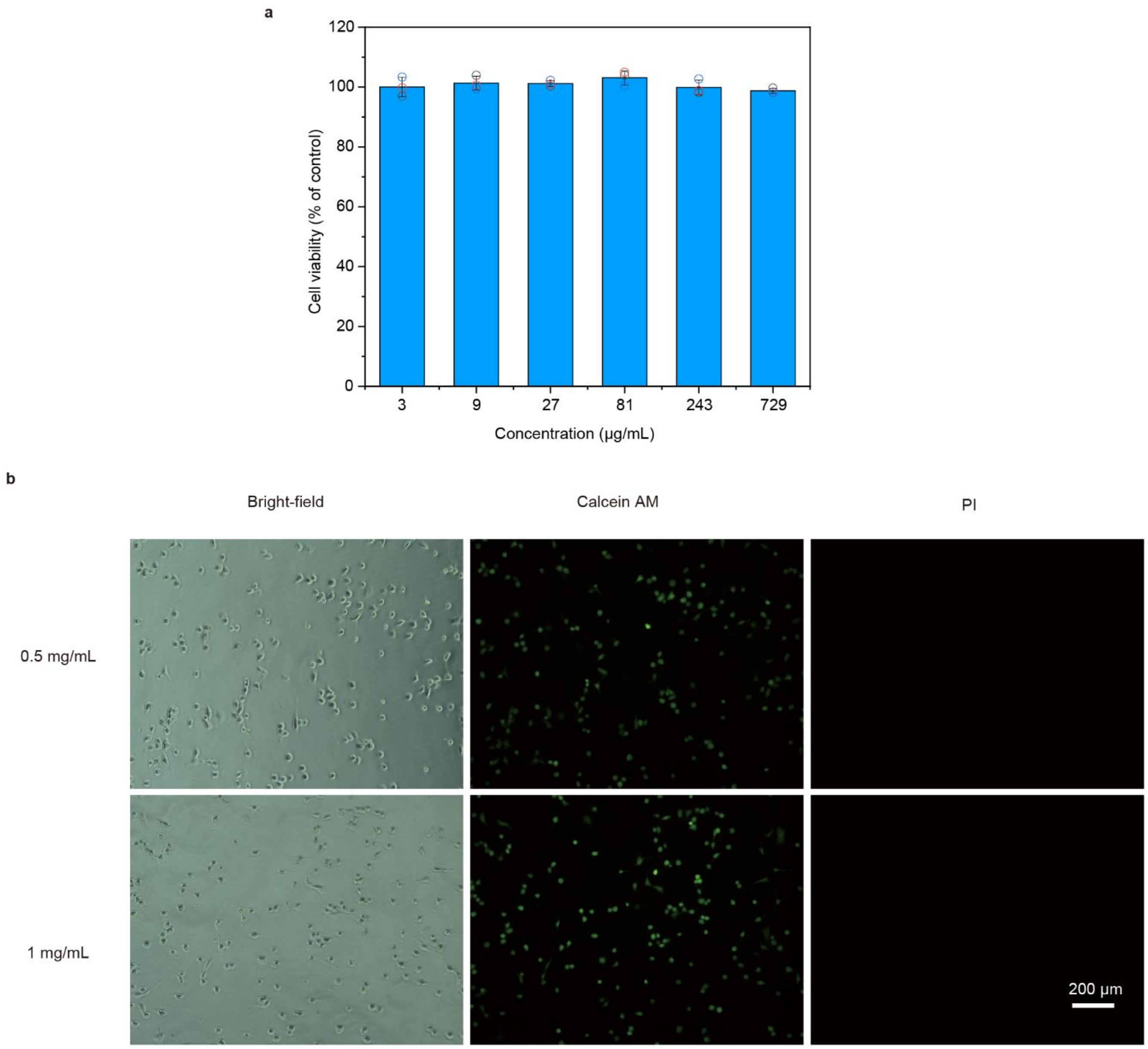

Supplementary Fig. 35 A biosafety assessment of unfolded BSA glue for hippocampal neuronal cells. **a** Viability of HT22 cells after incubation with various concentrations of BSA glue. Data are mean ± S.D. n = 3 independent samples per group. **b** Microscopic visualization of HT22 cells double stained with Calcein AM and PI.

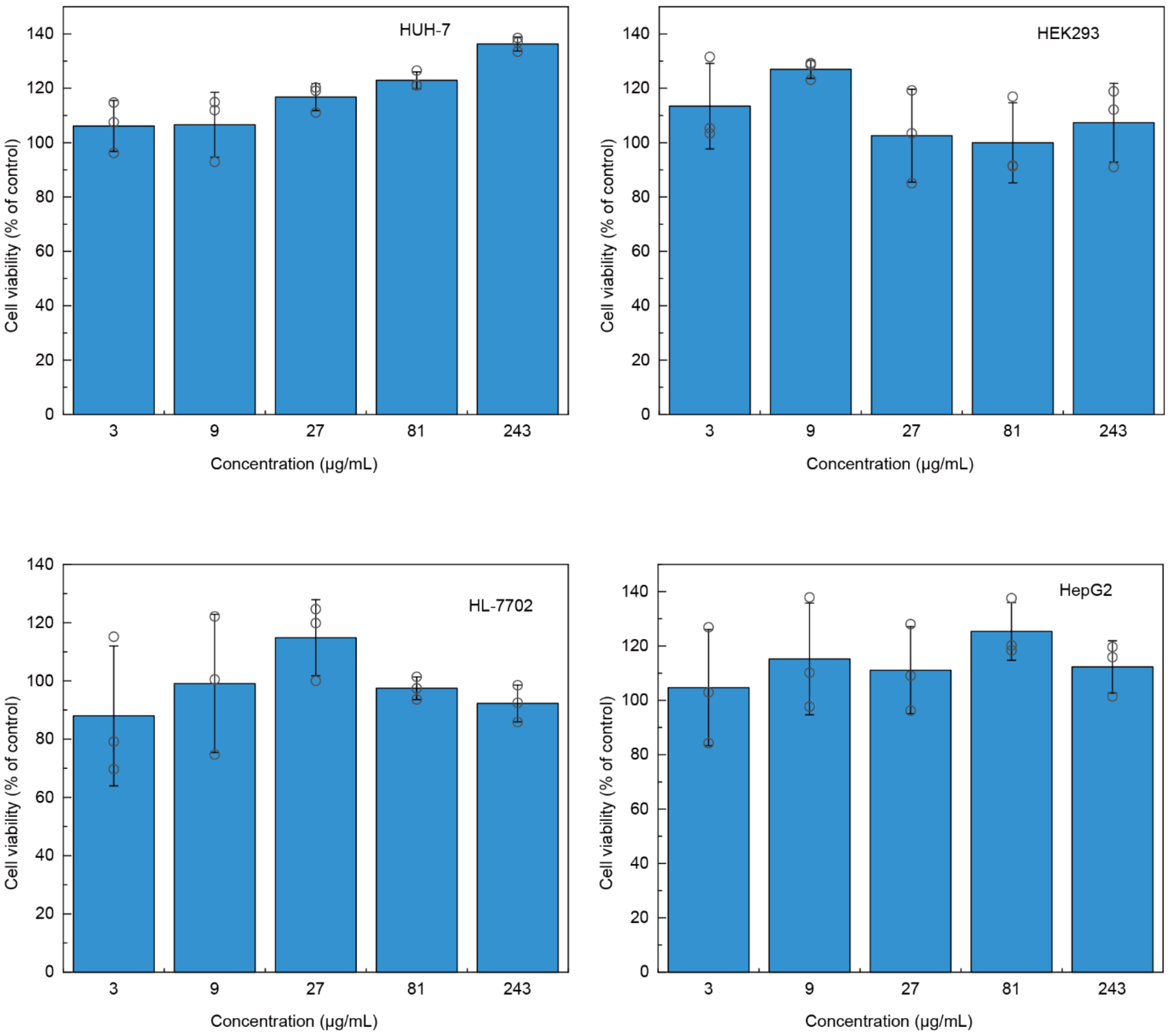

Supplementary Fig. 36 The relative viability of HUH-7, HepG2 (Human hepatocellular carcinoma), HEK293 (Human embryonic kidney cells), and HL-7702 (Normal human liver cells) after incubation with BSA glue. All data are mean ± S.D. n = 3 independent samples per group.

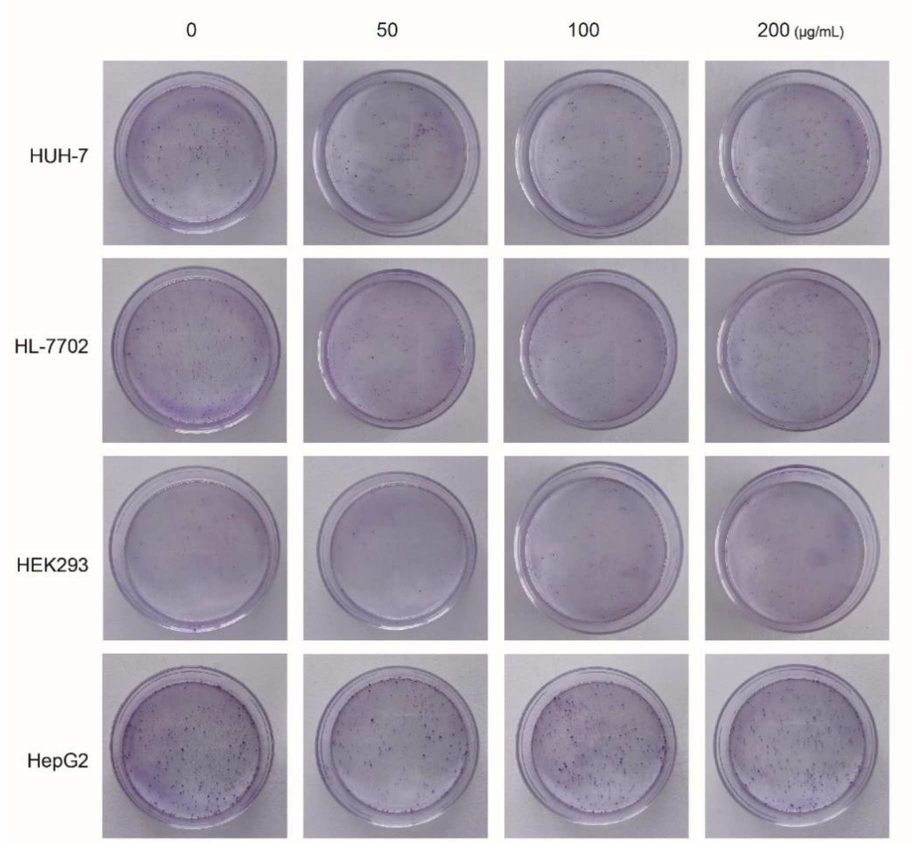

Supplementary Fig. 37 Photograph from conogenic assay of HUH-7, HepG2, HEK293 and HL-7702 cells incubated with different concentrations of unfolded BSA glue.

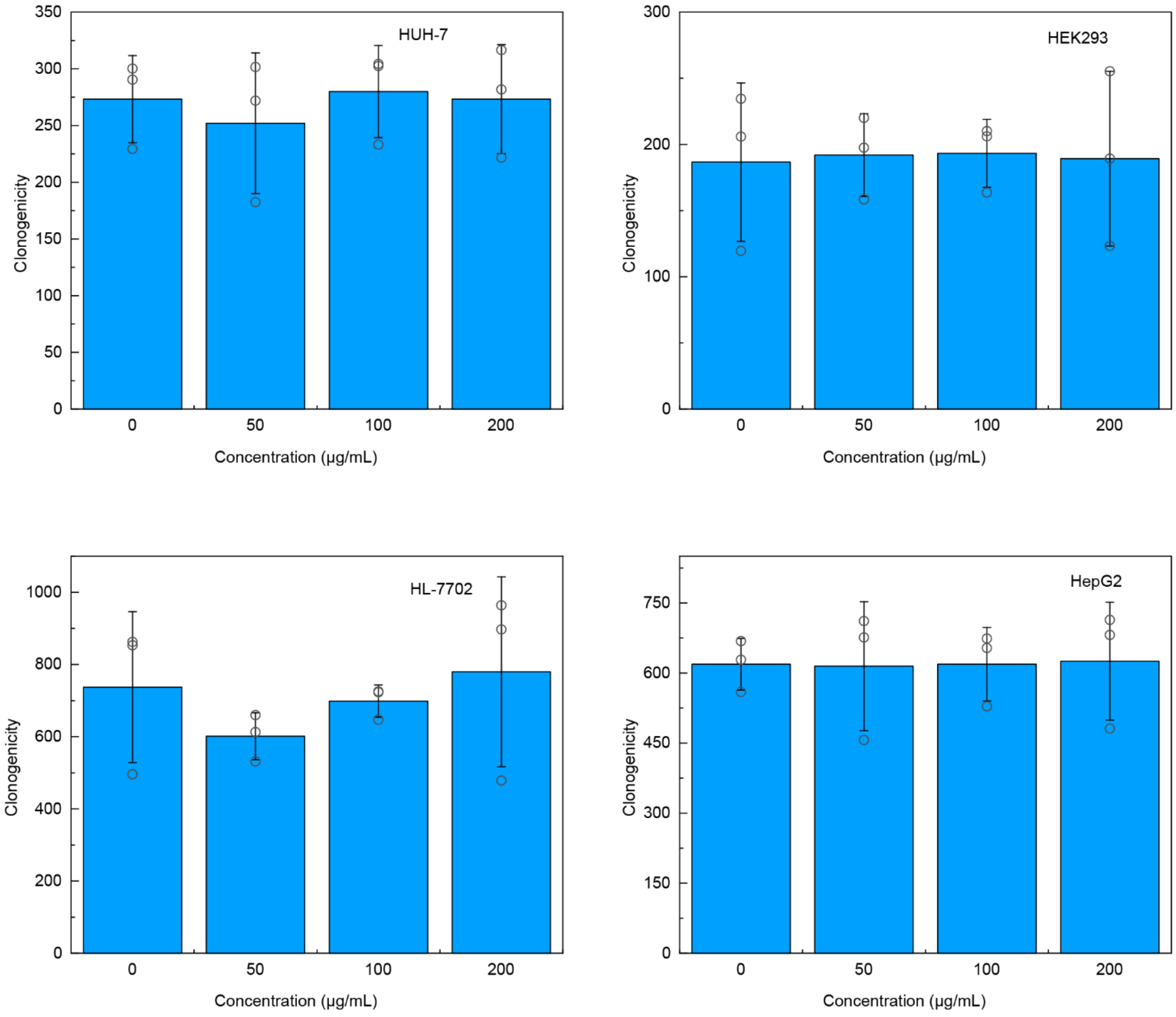

Supplementary Fig. 38 Clonogenicity determined by statistical analysis of Supplementary Fig. 37. All data are mean ± S.D. n = 3 independent samples per group.

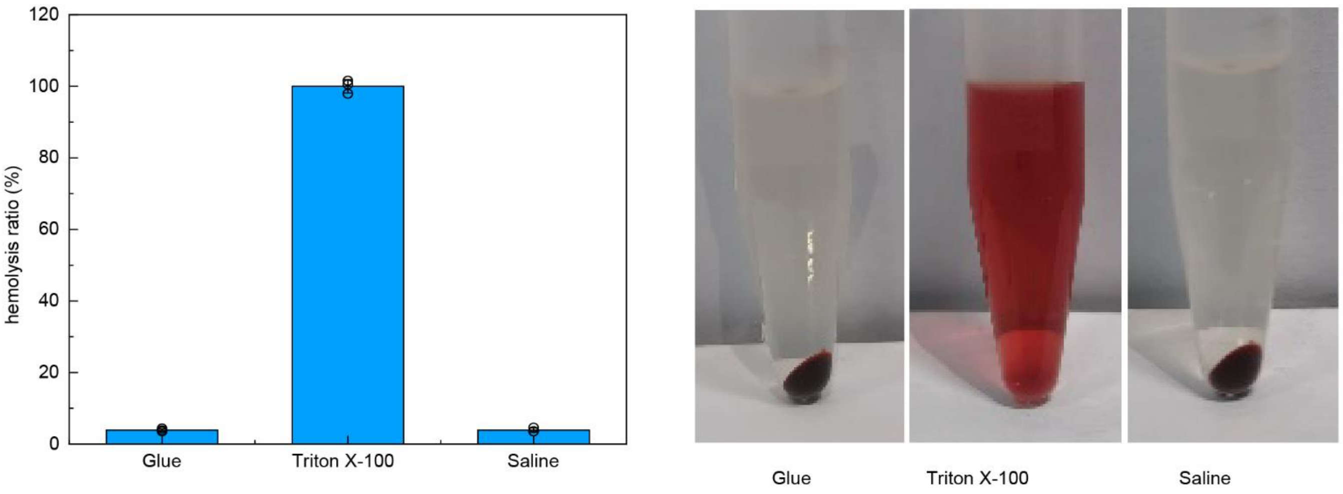

Supplementary Fig. 39 Hemolytic effect of the unfolded BSA glue on mouse red cell suspension (RBCs) at a concentration of 2 mg/mL, using water as a positive control and saline as a negative control. Data are mean ± S.D. n = 3 independent samples per group.

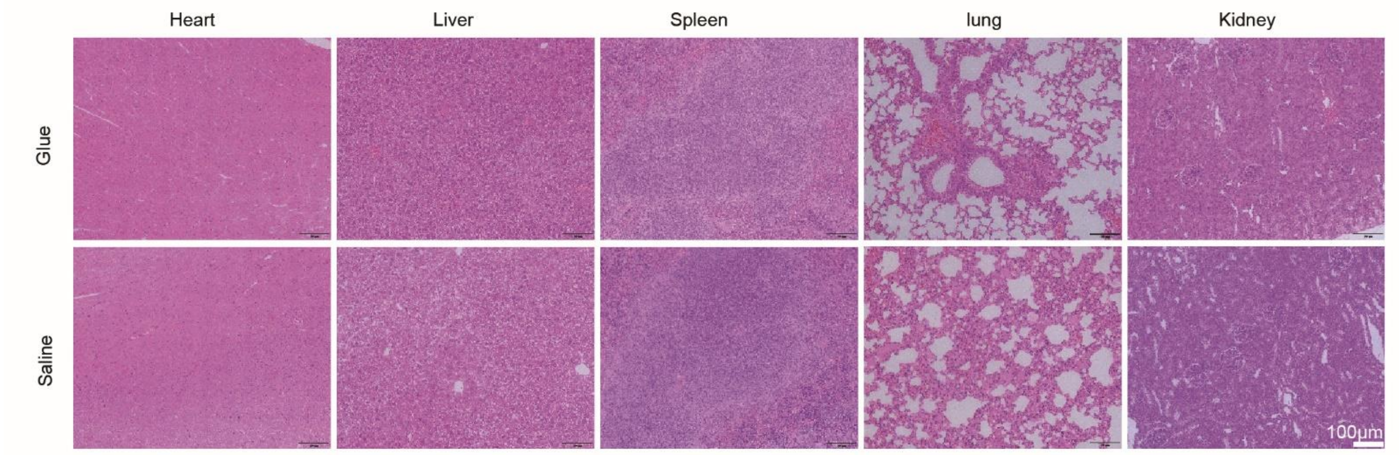

Supplementary Fig. 40 Histological images depicting the heart, liver, spleen, lung and kidneys of mice injected with 2 mg/mL unfolded BSA glue and normal saline for two weeks. The experiments were repeated independently at least three times with similar results.

.

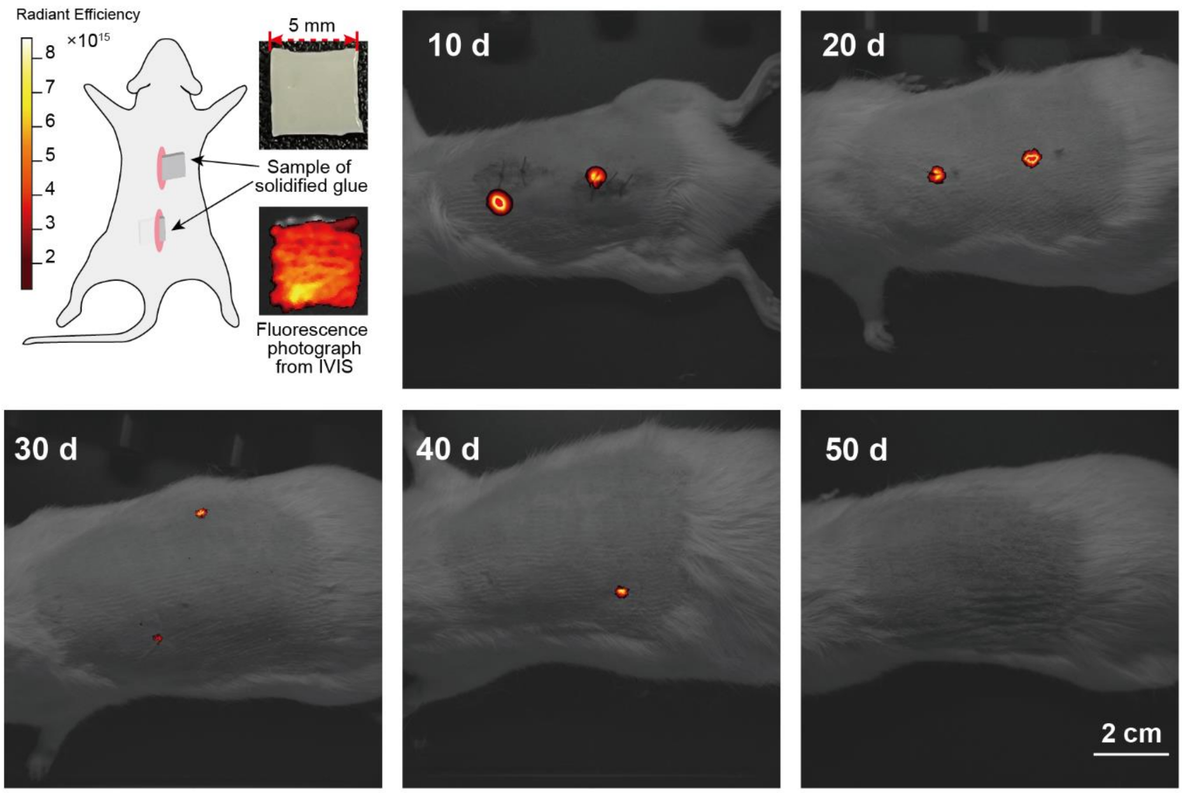

Supplementary Fig. 41 Diagram illustrating the procedure for implanting glue samples into rats, a digital and fluorescent photograph of a Cy5.5-labeled solidified glue sample, and fluorescence images of rats implanted with the glue samples during the in vivo degradation experiment.

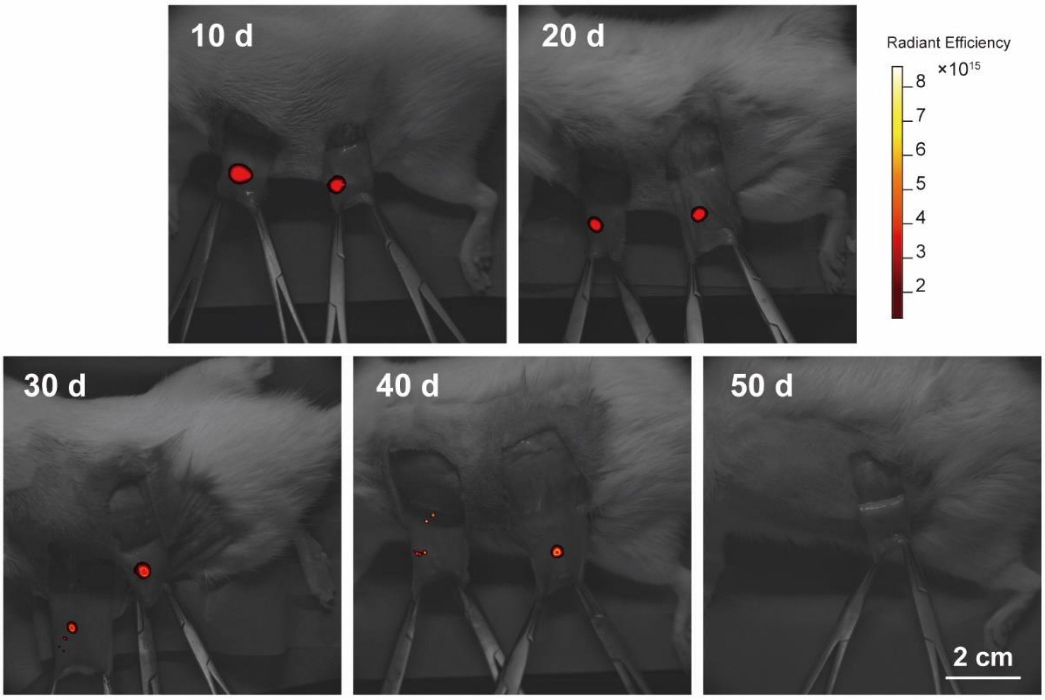

Supplementary Fig. 42 Fluorescence images of rats with open skin during the in vivo degradation experiment. The sample size of each group is 4.

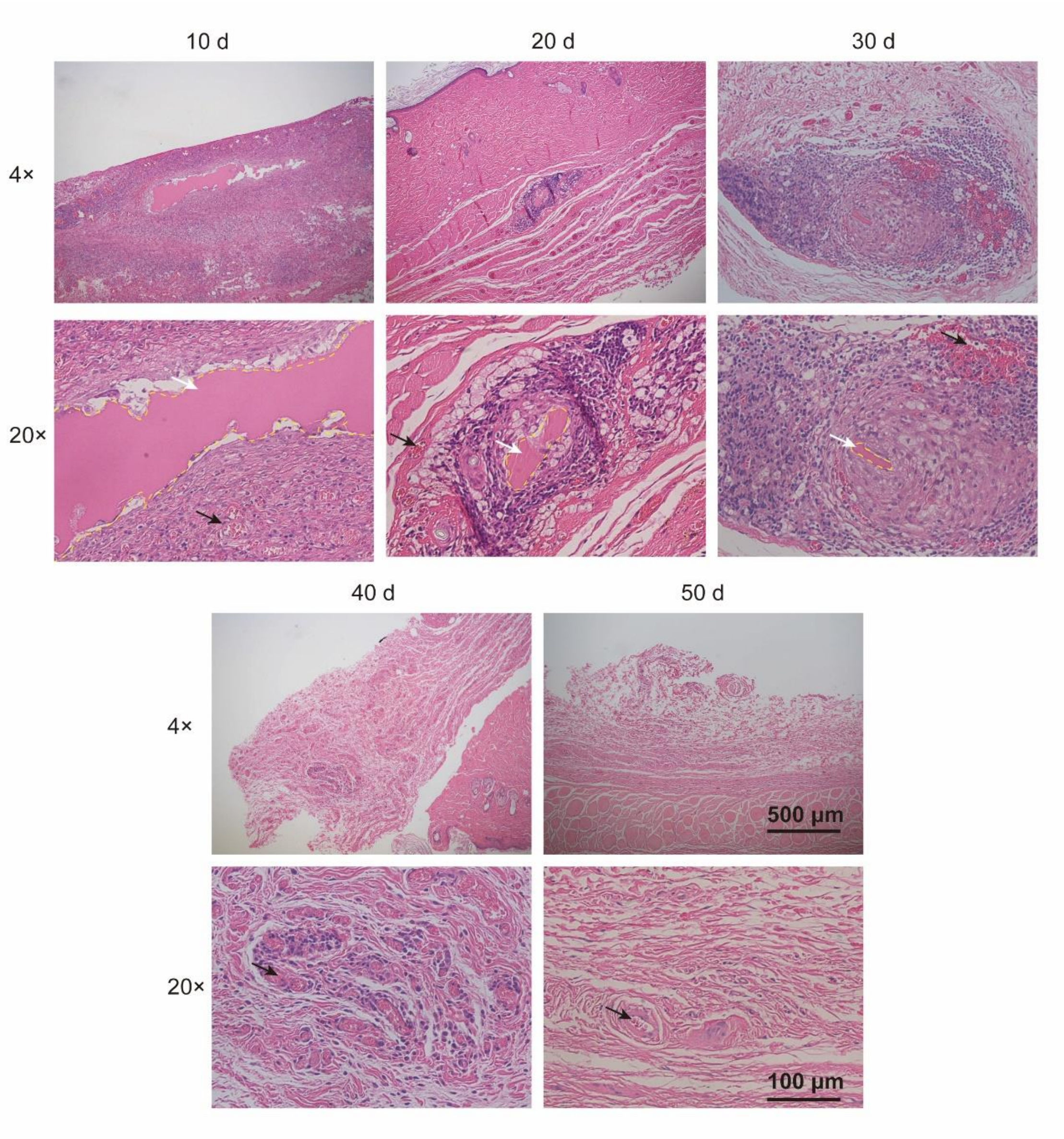

Supplementary Fig. 43 Hematoxylin and eosin (H&E) staining images of tissue around samples. The white arrow points to the glue sample and the black arrow points to the newly formed blood vessels. The experiments were repeated independently at least three times with similar results.

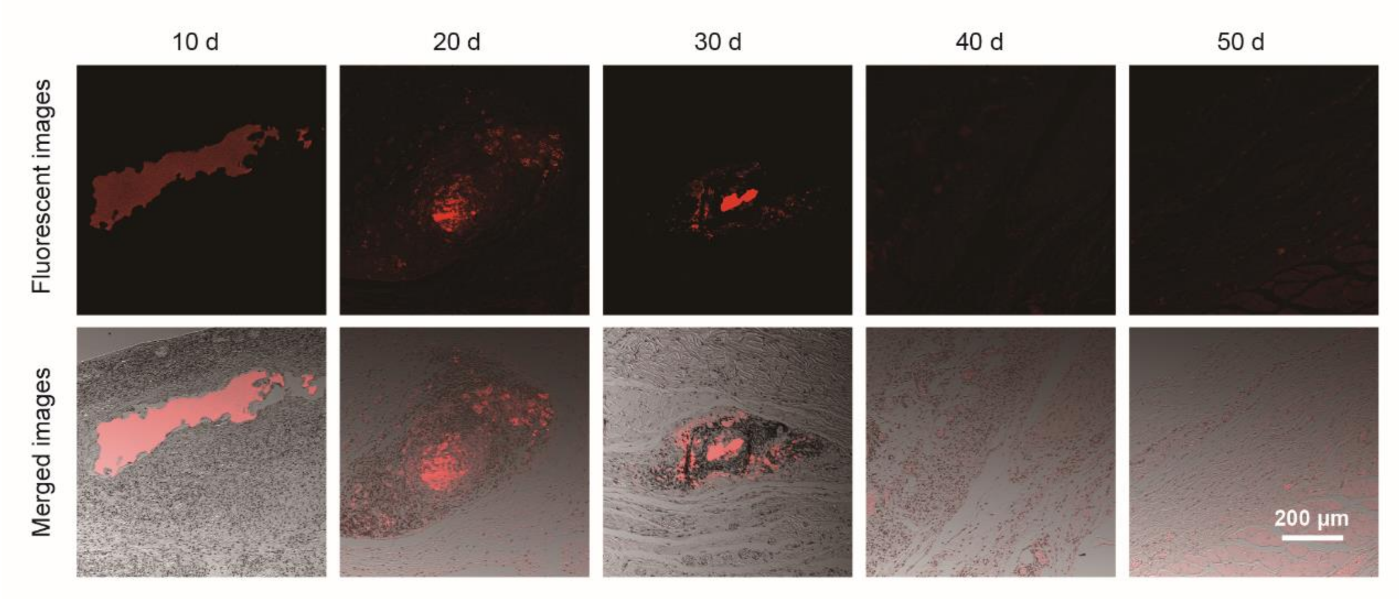

Supplementary Fig. 44 Laser scanning confocal fluorescence microscopy images of tissue around samples. The fluorescent channel is corresponding to the fluorescence of Cy5.5 (excitation, 640 nm; emission, 647LP nm). The experiments were repeated independently at least three times with similar results.

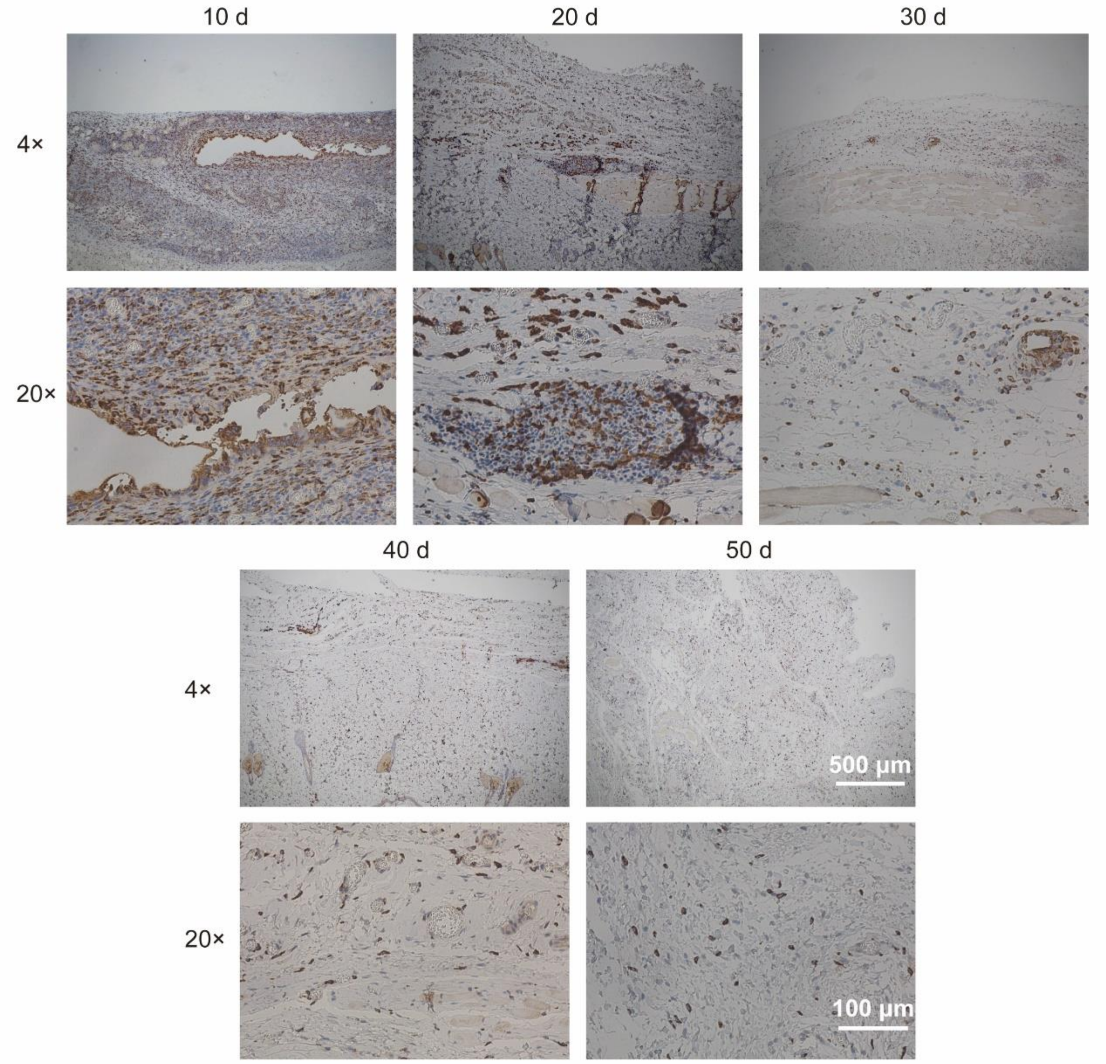

Supplementary Fig. 45 CD68 + staining images of tissue around glue samples under different days. The experiments were repeated independently at least three times with similar results.

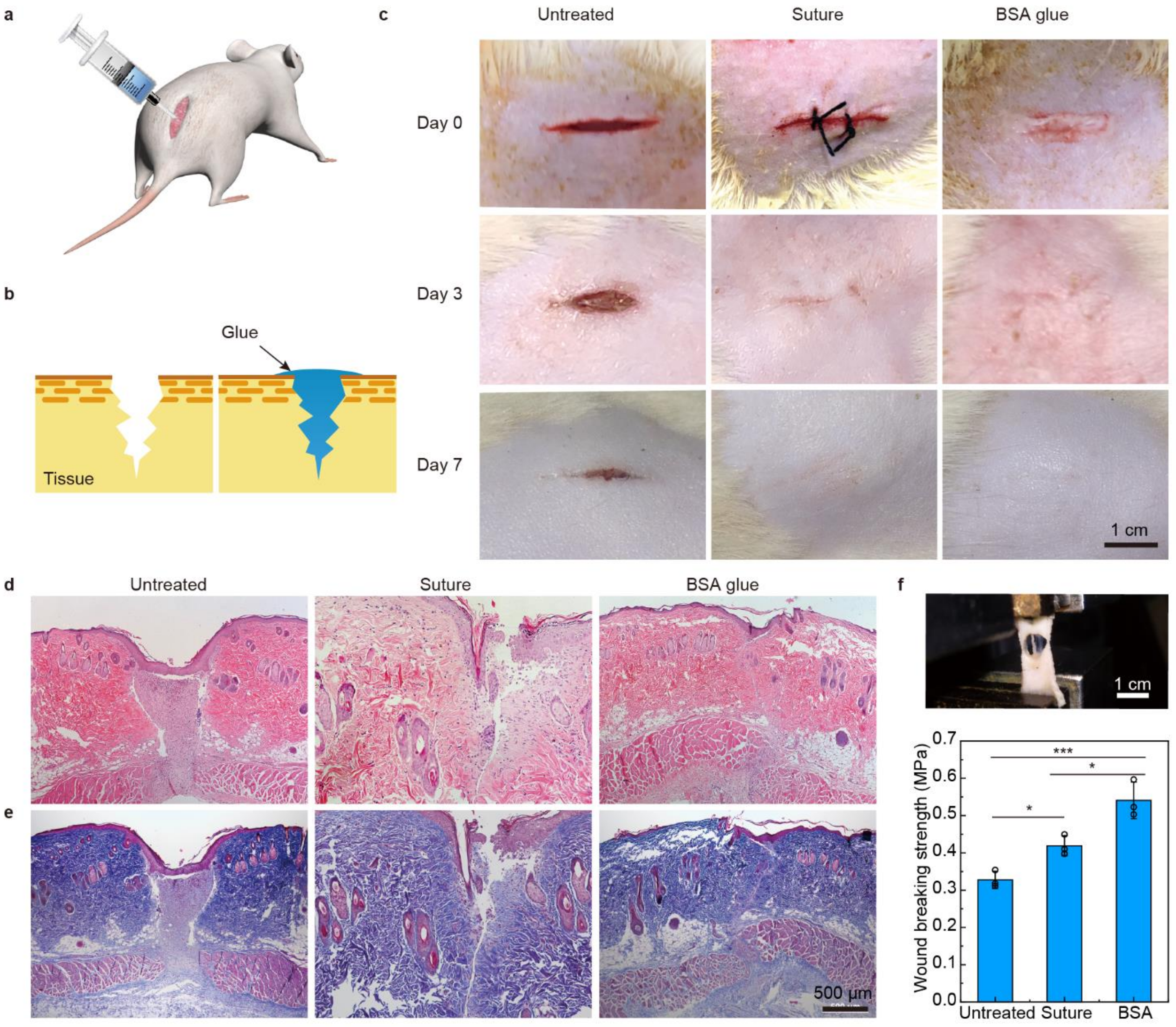

Supplementary Fig. 46 Application of unfolded BSA adhesive as a tissue glue. Illustration of rat incision treated with unfolded BSA glue (**a**, **b**). **c** Images of rat incisions untreated or treated with sutures and unfolded BSA glue after 0, 3 and 7 days. The sample size of each group is 5. Images of the wounded tissues stained with H&E (**d**) and MT (**e**) at day 7 after treatment. **f** Photograph of the tested sample and measurement of the wound breaking strength for skin closed by different manners at day 7. The statistical significances were calculated by one-way ANOVA by two-sided Tukey's multiple comparisons test. Adjusted P Values are Glue vs. Suture: 0.0127, Untreated vs. Suture 0.0430 and Untreated vs. Glue: 0.0007. Data are mean ± S.D. n = 3 independent rat samples per group.

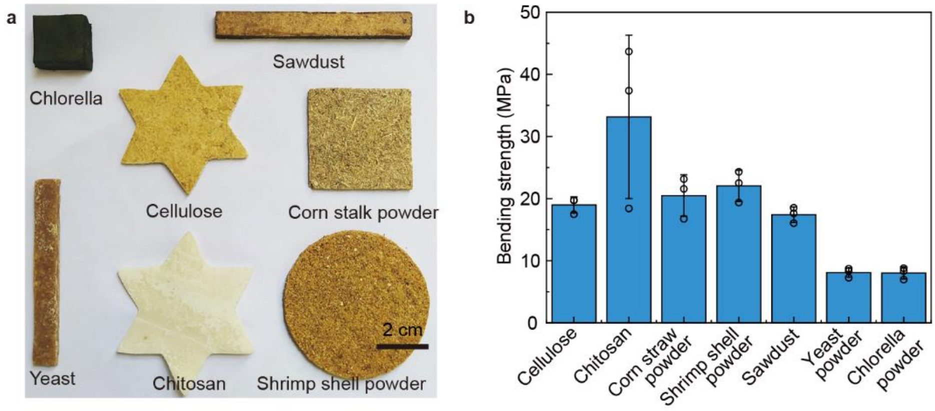

Supplementary Fig. 47 Photographs (**a**) and bending strength (**b**) of soyabean protein-based glue-bonded block materials, including chlorella, yeast, pine sawdust, corn stalk powder, cellulose powder, shrimp shell powder and chitosan powder. Data are mean ± S.D. n = 3 independent samples per group.

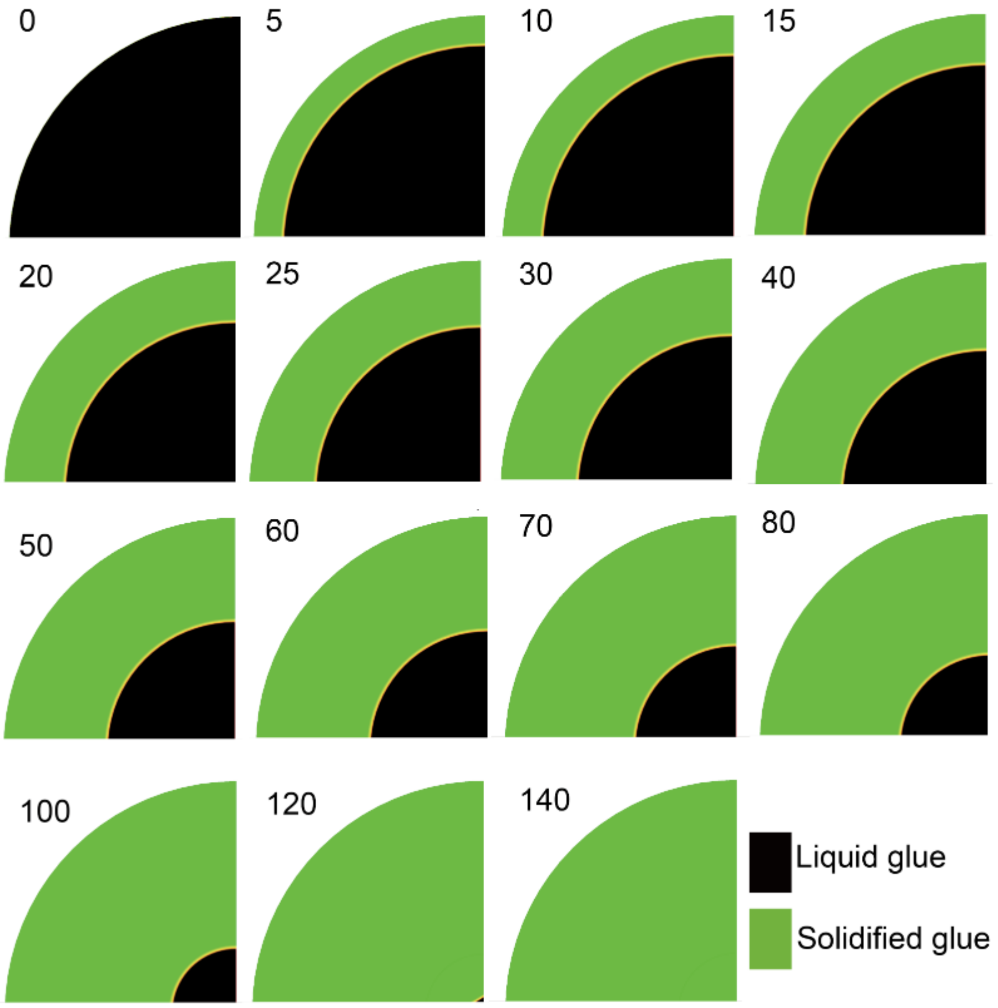

Supplementary Fig. 48 Time-dependent solidification process of the unfolded BSA glue between two glass slides obtained from the finite element simulation results. The unit of the number is minute.

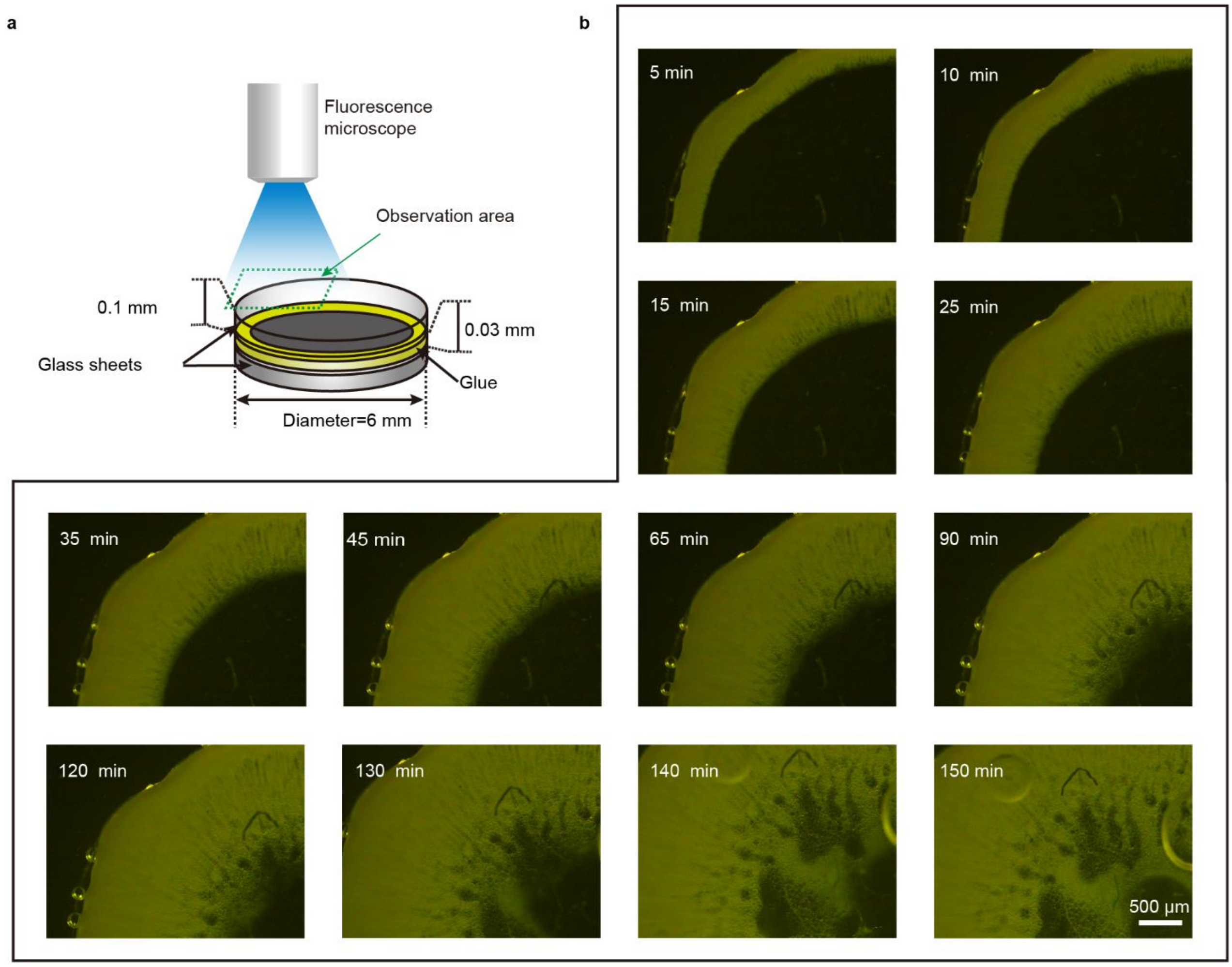

Supplementary Fig. 49 ThT staining fluorescence microscopy images of the solidification process of the unfolded BSA glue between two glass slides. **a** Schematic of the observation method. Two round glass slides (6 mm in diameter, 0.1 mm in thickness) were bonded by the glue (0.03 mm in thickness). Due to the limitation of the size of view field of microscope, 1/4 sector of the glass slide was observed. **b** The ThT staining fluorescence microscopy images of the glue-bonded slides at different time. The experiments were repeated independently at least three times with similar results.

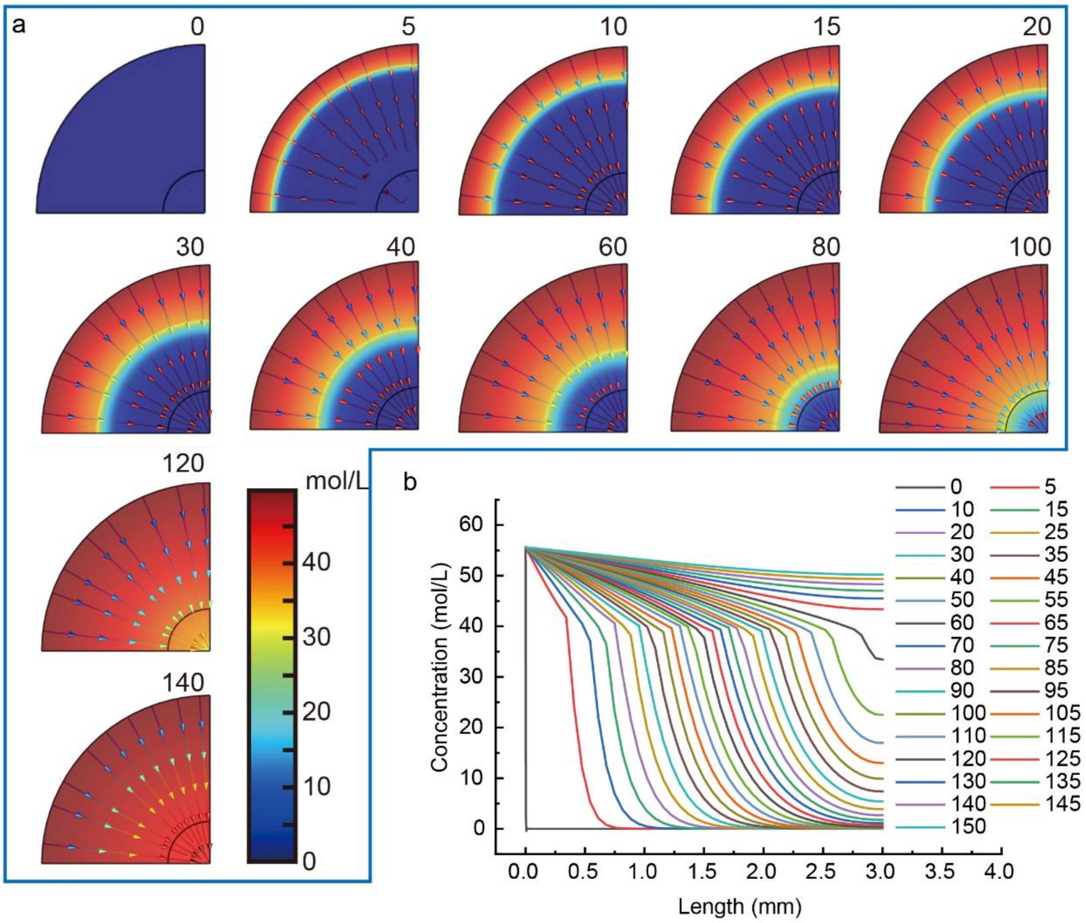

Supplementary Fig. 50 Finite element analysis of the diffusion process of water in the glue between two round slides after immersion in water. **a** Time-dependent distribution of the concentration of water in the glue. **b** The corresponding concentration curves of the concentration of water along the radial direction. The unit of the number is minute.

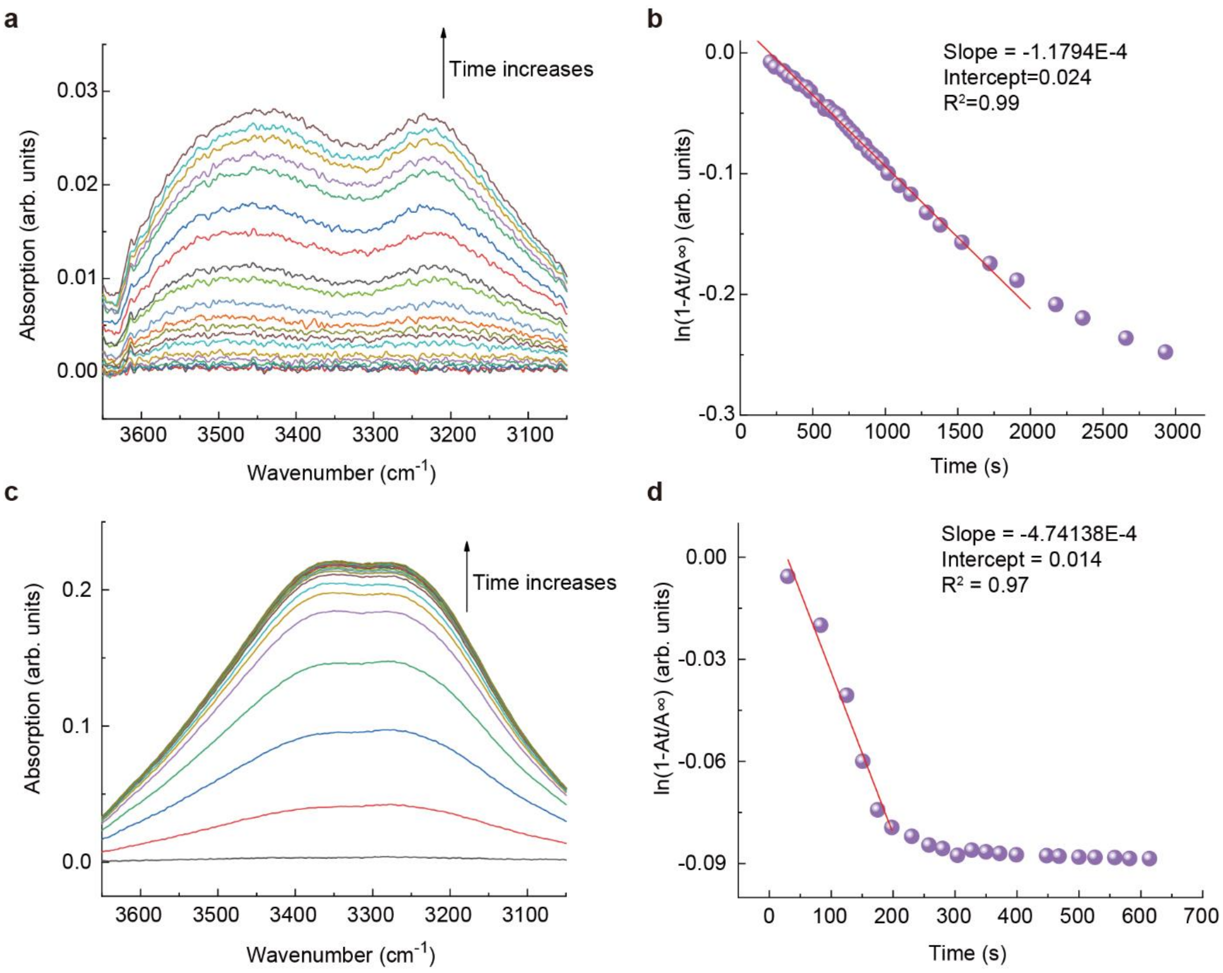

Supplementary Fig. 51. Measurement of the water diffusion coefficient by FTIR-ATR spectra. The FTIR-ATR spectra of the unfolded BSA (**a**) and the corresponding curve built by Equation 5 (**b**). The FTIR-ATR spectra of the solidified BSA (**c**) and the corresponding curve built by Equation 5 (**d**). The wavenumber selected for fitting was 3278 cm^{-1}.

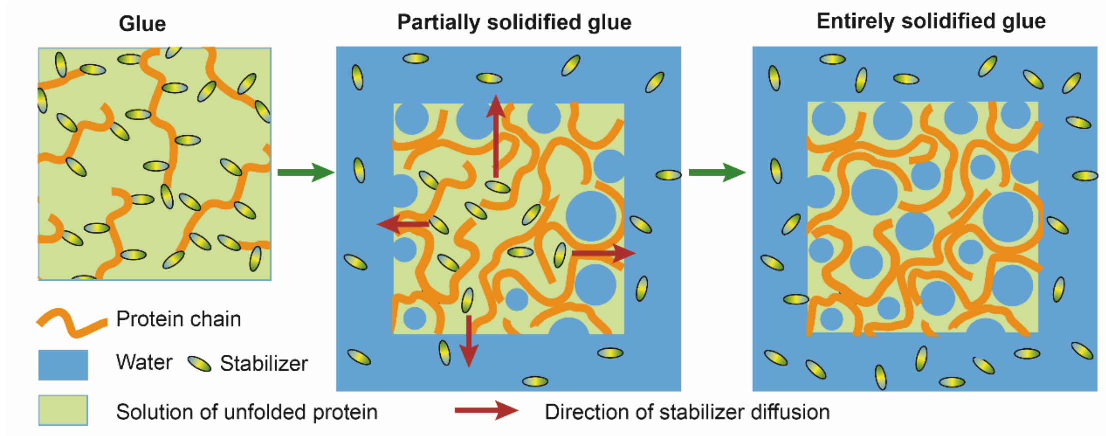

Supplementary Fig. 52 Schematic of the formation process of the water-filled cell during the solidification process of the protein-based glue.

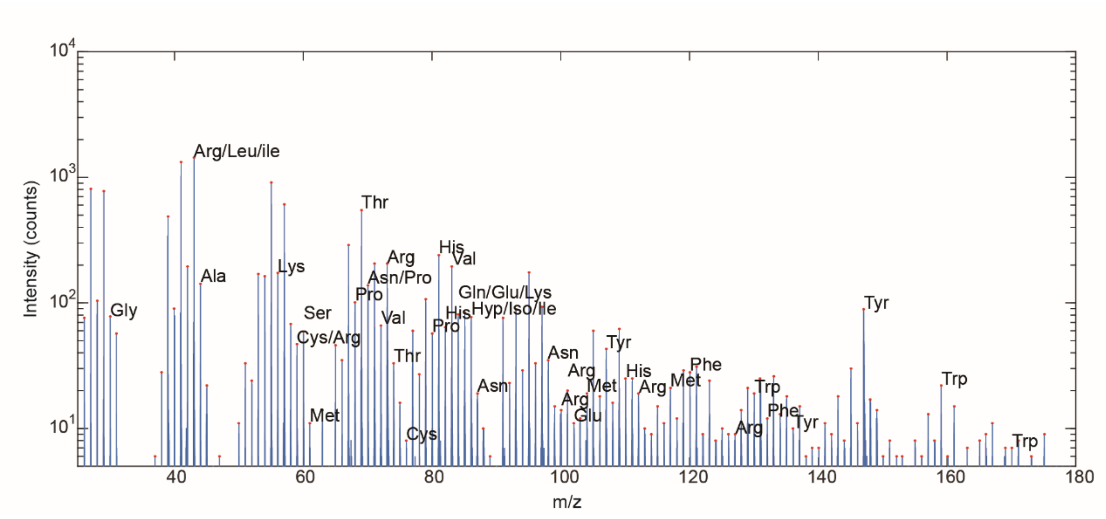

Supplementary Fig. 53 TOF-SIMS (Polarity: Positive) of the surface of solidified unfolded BSA glue.

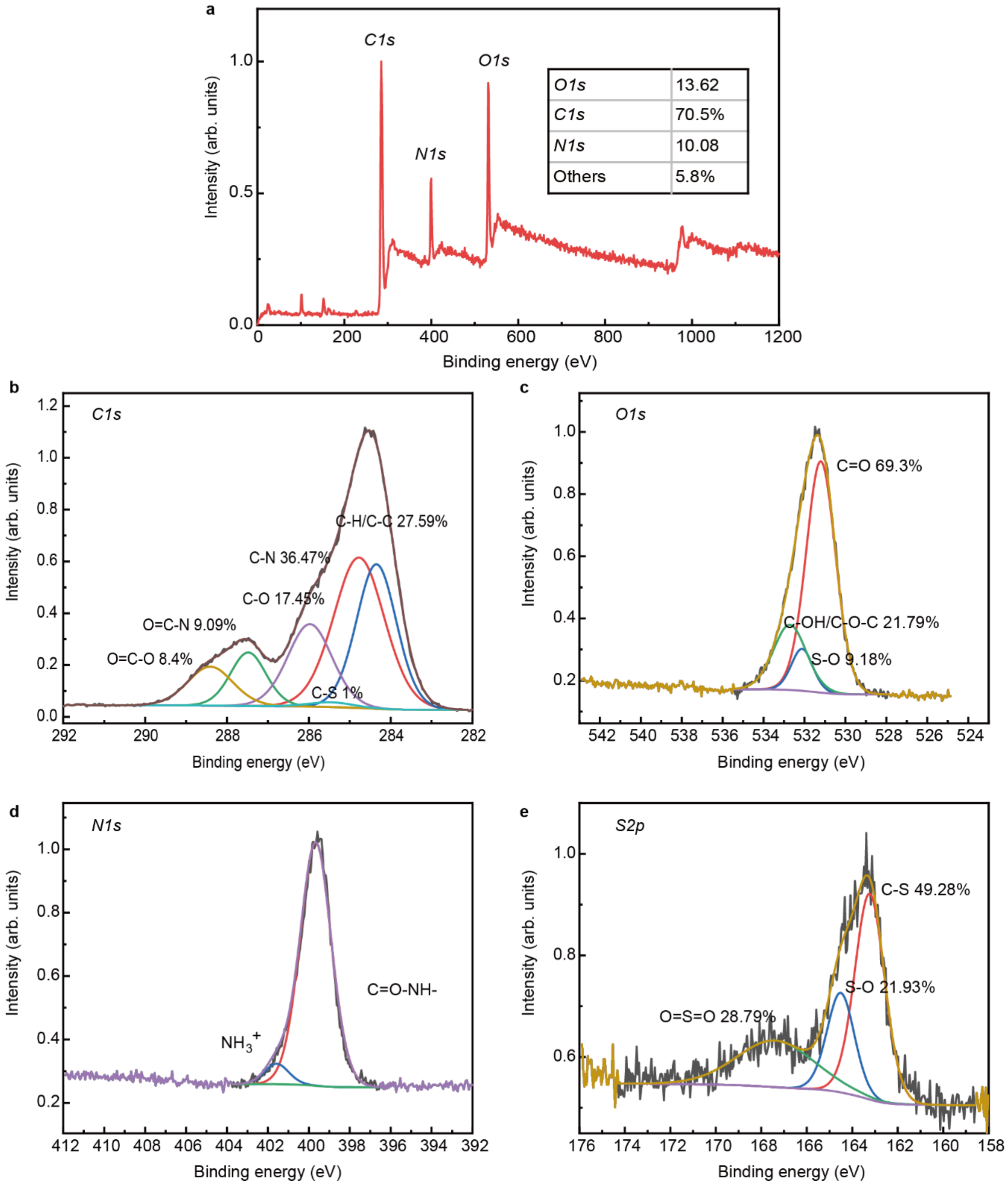

Supplementary Fig. 54 XPS spectra of the solidified glue. **a** Survey spectra of the solidified unfolded BSA. High-resolution scans of the XPS spectra of *C1s* (**b**), *O1s* (**c**), *N1s* (**d**) and *S2p* (**e**) for the solidified unfolded BSA glue. For its sensitivity to the surface (~5-10 nm), XPS was used to detect the surface chemistry of the solidified glue. The surface typically presented functional groups, including thiols (C-S), aliphatic carbon (C-H/C-C), amines (C-N), hydroxyls (C-O), amides (O=C-N) and carboxyl groups (O=C-O), amide (O=C-NH-) and quaternary ammonium (NH_3^+).

3. Supplementary tables

Supplementary Tab. 1 The parameters used in COMSOL.

Variable name	Description	Value
C_c	Critical solidification concentration	38.92 mol/L
D_0	Diffusivity of water in glue	7.84e-11 m^2/s
D_1	Diffusivity of water in solidified glue	7.2217e-10 m^2/s
C_0	Initial concentration of water	55.6 mol/L
R	Radius of the glass sheet	6 mm
T	Thickness of the glue	0.03 mm

Supplementary Tab. 2 Acute toxicity in mice (n=8).

Day	Death	Intraocular hypertension	Hair messy	Difficult breathing	Convulsion	Astasia	Hematuria	Coma
1	0	0	0	0	0	0	0	0
2	0	0	0	0	0	0	0	0
3	0	0	0	0	0	0	0	0
4	0	0	0	0	0	0	0	0
6	0	0	0	0	0	0	0	0
8	0	0	0	0	0	0	0	0
10	0	0	0	0	0	0	0	0
12	0	0	0	0	0	0	0	0
14	0	0	0	0	0	0	0	0

Supplementary Tab. 3 Serum biochemical changes in SD rat after implantation of solidified glue

Test names	Unit	Reference range	Control	10 days	20 days	30 days	40 days	50 days
ALT (Alanine aminotransferase)	U/L	6-114	72.4±46.02	51.88±3.21	51.57±7.71	51.7±13.02	50.89±3.44	57.35±13.88
AST (Aspartate aminotransferase)	U/L	37-205	122.87±15.16	164.41±30.65	126.26±9.85	194.73±47.12	125.16±17.06	141.49±49.18
TBIL (Bilirubin, total)	umol/L	2.57-36.85	15.5±0.92	7.81±0.95	8.43±2.27	9.88±4.73	9.88±2	7.25±0.69
DBIL (bilirubin, direct)	umol/L	2.24-16.892	8.56±0.16	4.51±0.71	5.64±1.68	6.67±2.67	3.93±0.9	3.28±0.3
ALB (Albumin)	g/L	29-48	34.33±2.79	28.85±0.69	29.2±0.85	27.08±6.51	33.43±1.17	33.9±1.36
ALP (Alkaline phosphatase)	U/L	12.04-610.97	147.97±58.04	353.38±41.7	227.37±7.94	225.34±68.9	214.85±27.57	275.15±34.78
γ-GT (Gamma-glutamyl transpeptidase)	U/L	0.5-5.3	3.57±1.41	2.26±0.53	2.6±0.8	3.19±1.21	1.3±0.27	1.37±0.3
TBA (serum bile acid)	umol/L	0-8.51	8.47±3.15	7.97±2.69	7.68±3	7.81±3.82	7.41±4.5	7.19±7.38
Urea	mmol/L	3.48-8.11	4.51±0.46	4.28±1.08	2.87±0.39	3.5±0.82	4.82±0.4	5.88±0.38
CREA(Creatinine)	umol/L	10.90-118.07	48.22±7.34	26.65±3.08	27.74±2.61	31.78±8.48	24.83±2.05	23.99±3.35
GLU (Glucose)	mmol/L	4.98-15.95	10.37±2.39	10.76±1.55	12.19±2.55	14.56±3.78	12.36±2.01	13.79±4.86
Ca^{2+}	mmol/L	2.3-3.25	2.32±0.02	2.2±0.04	2.36±0.05	2.06±0.2	2.1±0.05	2.22±0.08
Cl^{-}	mmol/L	94-116	106.74±0.7	104.43±1.42	96.4±1.05	109.3±0.18	110.19±0.85	107.04±3.96
Na^{+}	mmol/L	139-155	152.15±0.45	134.05±1.53	132.27±1.99	153.12±5.61	148.07±1.78	146.6±2.24
K^{+}	mmol/L	3.6-7.7	5.24±0.17	4.6±0.36	5.54±0.27	5.14±0.77	4.27±0.89	4.55±0.15

* Reference ranges were modified from Loeb et al (1999) (Loeb, WF and Quimby, FW. 1999. The Clinical Chemistry of Laboratory Animals, 2nd ed. Philadelphia: Taylor & Francis USA.)

Supplementary Tab. 4 Positive TOF-SIMS amino acid ions.

Positive Ion	Amino Acid	Mass
CH_4N	Gly	30.0346
CH_3N_2	Arg	43.0163
C_2H_6N	Ala	44.0492
CHS	Cys	44.9772
C_3H_6N	Lys	56.05
C_2H_3S	Cys	58.9941
CH_5N_3	Arg	59.05
C_2H_6NO	Ser	60.0448
C_2H_5S	Met	61.0095
C_4H_6N	Pro	68.053
C_4H_5O	Thr	69.0381
C_3H_4NO	Asn	70.0331
C_4H_8N	Pro	70.0704
$C_3H_3O_2$	Ser	71.0156
$C_4H_{10}N$	Val	72.0862
C_3H_7NO	Arg	73.0527
C_3H_8NO	Thr	74.066
C_2H_6SN	Cys	76.0292
C_5H_6N	Pro	80.05
$C_4H_5N_2$	His	81.0383
C4H6N2	His	82.0527
C_5H_7O	Val	83.0526
C_4H_6NO	Gln/Glu	84.0474
$C_5H_{10}N$	Lys	84.0855
$C_5H_{12}N$	Ile	86.0982
C_3H_7N2O	Asn	87.0576
$C_3H_6NO_2$	Asp/Asn	88.0297
$C_4H_4NO_2$	Asn	98.0144
$C_4H_{10}N_3$	Arg	100.0795
$C_4H_{11}N_3$	Arg	101.0963
$C_4H_8NO_2$	Glu	102.0415
$C_4H_{10}NS^+$	Met	104.05
C_7H_7O	Tyr	107.0505
$C_5H_8N_3$	His	110.0759
$C_5H_{10}N_3$	Arg	112.087
C_5H_9SO	Met	117.04
$C_8H_{10}N$	Phe	120.0874
$C_6H_{11}N_2O$	Arg	127.0868
C_9H_8N	Trp	130.0685
C_9H_7O	Phe	131.0459
C_9H_8O	Phe	132.06

Positive Ion	Amino Acid	Mass
$C_8H_{10}NO$	Tyr	136.0831
$C_9H_7O_2$	Tyr	147.04
$C_{10}H_{11}N_2$	Trp	159.0897
$C_{11}H_8NO$	Trp	170.0666

References

1. Döppers, L., Sammon, C., Breen, C. & Yarwood, J., FTIR–ATR studies of the sorption and diffusion of acetone/water mixtures in poly(vinyl alcohol). *Polymer* **47,** 2714 (2006).

2. Shiraki, K., Nishikawa, K. & Goto, Y., Trifluoroethanol-induced Stabilization of the α-Helical Structure of β-Lactoglobulin: Implication for Non-hierarchical Protein Folding. *Journal of Molecular Biology* **245,** 180 (1995).
