# Supplementary material for: Synthesis of robust underwater glues from common proteins via unfolding-aggregating strategy
Source: Nat Commun. 2023 Aug 24;14:5145. doi: 10.1038/s41467-023-40856-z (PMC10449925; doi:10.1038/s41467-023-40856-z)
Supplement: Supplementary file 3 — Description of Additional Supplementary Files [file 41467_2023_40856_MOESM3_ESM.pdf]

Description of Additional Supplementary Files

File Name: Supplementary Movie 1
Description: The trajectory of unfolded insulin bonding two silica sheets recorded from steered MD simulation

File Name: Supplementary Movie 2
Description: Writing words in water by using the unfolded BSA glue and its good stability against tap water washing

File Name: Supplementary Movie 3
Description: The instant adhesion of unfolded BSA glue for weight in water

File Name: Supplementary Movie 4
Description: The unfolded BSA glue bonded nylon boards can lift an adult (75kg). The video obtained informed consent from the research participants.

File Name: Supplementary Movie 5
Description: Comparing the stability of unfolded BSA glue and mussels byssus bonded glass slices after being immersed in alkaline solution for 48 hours (pH =12)

File Name: Supplementary Movie 6
Description: Patching the rubber tubing with a 5 mm hole by using the unfolded BSA glue

File Name: Supplementary Movie 7
Description: Repairing the accidental puncture of inflatable boat by using the unfolded BSA glue. The video obtained informed consent from the research participants.

File Name: Supplementary Movie 8
Description: Patching the pig bladder with a 5 mm hole by using the unfolded BSA glue

File Name: Supplementary Movie 9
Description: Using the unfolded BSA glue as a tissue adhesive to closure the incision of rat

File Name: Supplementary Movie 10
Description: Fixing the sand in trough against water flow by using the unfolded BSA glue
