# Supplementary material for: Synthesis of robust underwater glues from common proteins via unfolding-aggregating strategy
Source: Nat Commun. 2023 Aug 24;14:5145. doi: 10.1038/s41467-023-40856-z (PMC10449925; doi:10.1038/s41467-023-40856-z)
Supplement: Supplementary file 14 — Reporting Summary [file 41467_2023_40856_MOESM14_ESM.pdf]

nature portfolio

Corresponding author(s): Peng Yang
Last updated by author(s): Aug 7, 2023

Reporting Summary

Nature Portfolio wishes to improve the reproducibility of the work that we publish. This form provides structure for consistency and transparency in reporting. For further information on Nature Portfolio policies, see our Editorial Policies and the Editorial Policy Checklist.

Statistics

For all statistical analyses, confirm that the following items are present in the figure legend, table legend, main text, or Methods section.

n/a	Confirmed	
☐	☒	The exact sample size (*n*) for each experimental group/condition, given as a discrete number and unit of measurement
☐	☒	A statement on whether measurements were taken from distinct samples or whether the same sample was measured repeatedly
☐	☒	The statistical test(s) used AND whether they are one- or two-sided<br>*Only common tests should be described solely by name; describe more complex techniques in the Methods section.*
☒	☐	A description of all covariates tested
☐	☒	A description of any assumptions or corrections, such as tests of normality and adjustment for multiple comparisons
☐	☒	A full description of the statistical parameters including central tendency (e.g. means) or other basic estimates (e.g. regression coefficient) AND variation (e.g. standard deviation) or associated estimates of uncertainty (e.g. confidence intervals)
☐	☒	For null hypothesis testing, the test statistic (e.g. *F*, *t*, *r*) with confidence intervals, effect sizes, degrees of freedom and *P* value noted<br>*Give P values as exact values whenever suitable.*
☒	☐	For Bayesian analysis, information on the choice of priors and Markov chain Monte Carlo settings
☒	☐	For hierarchical and complex designs, identification of the appropriate level for tests and full reporting of outcomes
☒	☐	Estimates of effect sizes (e.g. Cohen's *d*, Pearson's *r*), indicating how they were calculated

Our web collection on statistics for biologists contains articles on many of the points above.

Software and code

Policy information about availability of computer code

Data collection	Gromacs 5.15, Chimera 1.14, LINCS algorithm, MMPBSA algorithm, COMSOL Multiphysics 5.5
Data analysis	All statistical analyses were performed on Graphpad Prism (version 9.0) or Excel 2019; CasaXPS (version 2.3.16) was used to analyze X-ray photoelectron spectroscopy; Chiascan Spectrometer Control Panel Application (version 4.1.5) was used to process circular dichroism spectrum; Peak Fit (version 4) was used to analyze Fourier transform infrared (FTIR) spectra; Fit2D (version 17.077) was used to analyze Synchrotron small-angle X-ray scattering; ImageJ (version 1.8.0) was used to calculated the size of Mussel byssus.

For manuscripts utilizing custom algorithms or software that are central to the research but not yet described in published literature, software must be made available to editors and reviewers. We strongly encourage code deposition in a community repository (e.g. GitHub). See the Nature Portfolio guidelines for submitting code & software for further information.

Data

Policy information about availability of data

All manuscripts must include a data availability statement. This statement should provide the following information, where applicable:

- Accession codes, unique identifiers, or web links for publicly available datasets
- A description of any restrictions on data availability
- For clinical datasets or third party data, please ensure that the statement adheres to our policy

The data supporting the findings from this study are available within the Article, Supplementary Information or Source Data file. Source data are provided with this paper.

Human research participants

Policy information about studies involving human research participants and Sex and Gender in Research.

Reporting on sex and gender	N/A
Population characteristics	N/A
Recruitment	N/A
Ethics oversight	N/A

Note that full information on the approval of the study protocol must also be provided in the manuscript.

Field-specific reporting

Please select the one below that is the best fit for your research. If you are not sure, read the appropriate sections before making your selection.

- [x] Life sciences
- [ ] Behavioural & social sciences
- [ ] Ecological, evolutionary & environmental sciences

For a reference copy of the document with all sections, see nature.com/documents/nr-reporting-summary-flat.pdf

Life sciences study design

All studies must disclose on these points even when the disclosure is negative.

Sample size	No statistical method was used to predetermine sample size. Sample size were based on preliminary experimentation. For in wound closure experiments, there are 5 rats per group. For In vivo degradability experiments, there are 4 rats per group. For acute toxicity test, there are 8 mice per group. The sample sizes are sufficient to identify differences between groups and minimize animal sacrifice. Details regarding sample size of all experiments are provided in the Methods section and figure legends.
Data exclusions	No data were excluded from the analysis.
Replication	Experiments were replicated and experimental findings were reproducible. Experimental repeat numbers are reported in Figure Legends.
Randomization	All samples, cells and animals were allocated randomly into experimental groups.
Blinding	In all experiments, colleagues who assisted in data collection were blinded to group allocation during data analysis.

Reporting for specific materials, systems and methods

We require information from authors about some types of materials, experimental systems and methods used in many studies. Here, indicate whether each material, system or method listed is relevant to your study. If you are not sure if a list item applies to your research, read the appropriate section before selecting a response.

Materials & experimental systems

n/a	Involved in the study	
[ ]	[x]	Antibodies
[ ]	[x]	Eukaryotic cell lines
[x]	[ ]	Palaeontology and archaeology
[ ]	[x]	Animals and other organisms
[x]	[ ]	Clinical data
[x]	[ ]	Dual use research of concern

Methods

n/a	Involved in the study	
[x]	[ ]	ChIP-seq
[x]	[ ]	Flow cytometry
[x]	[ ]	MRI-based neuroimaging

Antibodies

Antibodies used	CD68 Mouse monoclonal antibody (abcam, ab955), Lot: GR3380470-21
Validation	CD68 Mouse monoclonal antibody : Species: Human, Mouse, Rat, Rabbit; Application: WB, ICC, IHC-P.

Eukaryotic cell lines

Policy information about cell lines and Sex and Gender in Research

Cell line source(s)	HepG2, HUH-7, HL-7702, HEK293 and HT22 were bought from the national biomedical experimental cell resource bank (Beijing, China)
Authentication	None of the cell lines used were authenticated
Mycoplasma contamination	All cells tested negative for mycoplasma contamination.
Commonly misidentified lines (See ICLAC register)	No commonly misidentified cell lines were used in the study.

Animals and other research organisms

Policy information about studies involving animals; ARRIVE guidelines recommended for reporting animal research, and Sex and Gender in Research

Laboratory animals	SD rats, 5-8 weeks old; BALB/c mice, 6-8 weeks old. All mice or rates were housed in experimental animal room maintained on a 12:12-hour, light:dark cycle. The temperature and humidity were maintained ~20 °C and 45-55% respectively.
Wild animals	Mussels (perna viridis, 30-40g, about six month) were fished from the sea area of Rongcheng, Shandong Province by cutting the byssus. Immediately, the Mussels were transfer to a thermostatic water tank with local seawater under 22 °C. The water tank was transported to Xi'an under by vehicle. They were fed with Chlorella once a day. After the experiment finishing, they were released to their home sea area after the study.
Reporting on sex	The findings of this experiment apply to both females and males.
Field-collected samples	This study did not involve samples collected from the field.
Ethics oversight	All experiments were performed according to the guidelines of the laboratory animal care committee at Shaanxi Normal University(No. 2020209)

Note that full information on the approval of the study protocol must also be provided in the manuscript.
