# Supplementary material for: Synthesis of robust underwater glues from common proteins via unfolding-aggregating strategy
Source: Nat Commun. 2023 Aug 24;14:5145. doi: 10.1038/s41467-023-40856-z (PMC10449925; doi:10.1038/s41467-023-40856-z)
Supplement: Supplementary file 2 — Peer Review File [file 41467_2023_40856_MOESM2_ESM.pdf]

nature portfolio

Peer Review File

Synthesis of robust underwater glues from common proteins via unfolding-aggregating strategy

Open Access This file is licensed under a Creative Commons Attribution 4.0 International License, which permits use, sharing, adaptation, distribution and reproduction in any medium or format, as long as you give appropriate credit to the original author(s) and the source, provide a link to the Creative Commons license, and indicate if changes were made. In the cases where the authors are anonymous, such as is the case for the reports of anonymous peer reviewers, author attribution should be to 'Anonymous Referee' followed by a clear attribution to the source work. The images or other third party material in this file are included in the article's Creative Commons license, unless indicated otherwise in a credit line to the material. If material is not included in the article's Creative Commons license and your intended use is not permitted by statutory regulation or exceeds the permitted use, you will need to obtain permission directly from the copyright holder. To view a copy of this license, visit http://creativecommons.org/licenses/by/4.0/.

Editorial Note: This manuscript has been previously reviewed at another journal that is not operating a transparent peer review scheme. This document only contains reviewer comments and rebuttal letters for versions considered at Nature Communications.

Reviewer #3:
Remarks to the Author:
This manuscript is transfered to Nature Comm after the authors' revision. Compared to previous version, the revised manuscript is now well improved. But, several points should be still considered and revised.

1. Please provide essential information about Fig. 6g:
- the x- and y-axis titles of the inset graph
- equipment and X-ray wavelength (Å) used in XRD study.

2. Additional information is required to verify the correspondence between Fig. S43b, S43d and Equation 5. Please present the coefficient of determination (R2 value), and check whether the y-intercepts of regression line in the Fig. S43b and S43d match the value of ln(π/4) at Equation 5. Also, which wavenumber (cm-1) was selected as the characteristic peak from the FTIR-ATR spectra to be plotted?

3. From Fig. 3j, the CD spectrum of solidified glue around 215 nm, corresponding to the β-sheet, appears to be flat rather than has peak or valley. Can it be said that the β-sheet has significantly increased by solidification with this data, or is it possible to provide complementary data to support the claim?

Reviewer #4:
Remarks to the Author:
The adhesion to a surface is the cumulative effect of adhesive and cohesive forces. Maximizing one works against the other and the trick is to obtain the optimum of each component.
The destabilization of the proteins with a disulfide disruptor followed by the addition of trichloroethanol (TCE) to dissolve the protein converts that into a flowable liquid, which is able to make good contact with the surface. Removal of the organic TCE by immersion in water converts the protein back to a solid hence increasing adhesion. Although the result is interesting, there is nothing special in these results. It is what is expected from an understanding of interfacial science. The finding described here is not special as these results will not hold beyond being a publication. One cannot use a fluorinated solvent to apply a glue in a biomedical application, and probably not for commodity applications also due to the environmental footprint of fluorinated solvents. This is the challenge in designing glues for biomedical applications. Although several reports have demonstrated design of adhesives that do not use any solvent for application or the use of non-toxic components, they are not clinically advanced to displace current fibrin glues. The manuscript has not referenced these prior studies.
As summarized above, this manuscript is not suitable for publication in Nat. Commun.
There are also several other reasons why this manuscript is not suitable for publication, including the authors' propensity to use superlative and unsupported language that makes the manuscript sound more like an advertisement than a scientific report.
Then there are several (too many to keep track) of comparisons that are not fair or are inaccurate.
The experiments are not fully described to be repeatable. For example, the authors claim that these are underwater adhesives. (In underwater adhesives, the substrate preparation, glue application, and curing have to be done underwater). However, the experimental section does not provide sufficient

information of how these experiments were done. In fact, the lap shear experimental section says that 10L of glue was poured onto 1 cm x 1 cm glass slides. It is quite obvious that something is inaccurate in this description. There is only some video and images of application of the glue to a surface. This is not a substitute for detailed experimental protocols. Similarly, there are no details of the tensile experiments.

The mouse wound closure studies show deposition of the glue onto a dry skin wound. It is not surprising that a protein glue would stick to a dry skin with a narrow wound. What is not discussed is that the wound now has trichloroethanol in it, which would be toxic to the tissues. Additionally, the use of the adhesives in the wound experiments were done using urea as the 'stabilizer'. Was urea dissolved in water to be added to the protein? The use of urea and the resultant lap shear test results were not provided in the prior sections. The mechanism of action of urea and TCE cannot be the same. These were not described in the manuscript.

There are several experiments such as the above that are either not necessary or are distracting to the main message of the work.

It is understandable that MD simulations can predict interactions between proteins and surface. But is not easy to understand how MD simulations can translate those interactions into lap shear strength data.

Hence, due to the above I would recommend against publication in Nat. Commun.

Reviewer #1 (Remarks to the Author):

The authors have improved the manuscript by addressing the reviewer's comments. In revised manuscript, the authors addressed the concerns and questions about the toxicity of amyloid adhesives. In addition, they have adequately supplemented with references and requested data to support their claims.

Nevertheless, the stability studies of their materials are in vitro or preclinical studies, which are not complete to determine the complete harmlessness of the human body. In addition, I think it would be better if the description of the stability of their adhesive material was only at the level demonstrated by their data, and I recommend that the authors clearly state in the paper that further research on the stability of the human body is needed.

Responses: Thank you so much for your constructive suggestion. The stability of the glue in vivo is extremely important. We agree with the reviewer's comment, and add the following statement in the revised manuscript: "While cell and animal experiments have demonstrated the safety of glue, further research on the stability of glue in the human body is also necessary ". Furthermore, we have systematically analyzed the degradation performance of unfolded BSA glue in rats.

By mixing Cy5.5-labeled BSA into the glue, we achieved real-time observation of protein degradation in vivo. For the test samples, glue was pre-solidified in rectangular blocks (5×5×1 mm), so it would not flow freely in vivo and interfere with the observations. By implanting the sample into the back of the SD rat, the degradation of the glue can be observed in real-time using in vivo imaging systems (IVIS). As shown in Fig. RL1, the fluorescence emission range of the sample gradually decreases, and after 50 days of implantation, the fluorescence of the sample is barely visible. Opening the skin of the implanted area of the rat also shows that the fluorescence range of the sample decreases over time, indicating that this method can

indeed monitor the degradation of the glue sample (Fig. RL2). To further analyze the degradation process, we conducted tissue section analysis of the implanted area. Through confocal microscopy combined with H&E staining results, it can be seen that a large number of blood vessels are newly formed near the implantation site, and a large number of cells infiltrate the sample to degrade it (Fig. RL3, 4). Staining of macrophages with CD68 showed that after 10 days of sample implantation, the protein glue was infiltrated by a large number of macrophages, while after 50 days of sample implantation, the number of macrophages decreased significantly, indicating that degradation mainly depends on macrophages. This process is similar to the degradation of other degradable materials such as gelatin (Acta biomaterialia, 2011, 7(1): 278-286.). In summary, the unfolded protein glue has good degradability in vivo, and for sample blocks of 25 mm^3 (wet weight: 26.9 mg, dry weight: 2.8 mg), the degradation time is 50 days, and the degradation process mainly depends on macrophages.

In addition, we also conducted molecular analysis of various blood biochemical indicators in rats throughout the entire implantation period (Table RL1), and the results showed that the various indicators in the blood were within the normal range, indicating that the glue has good biological safety.

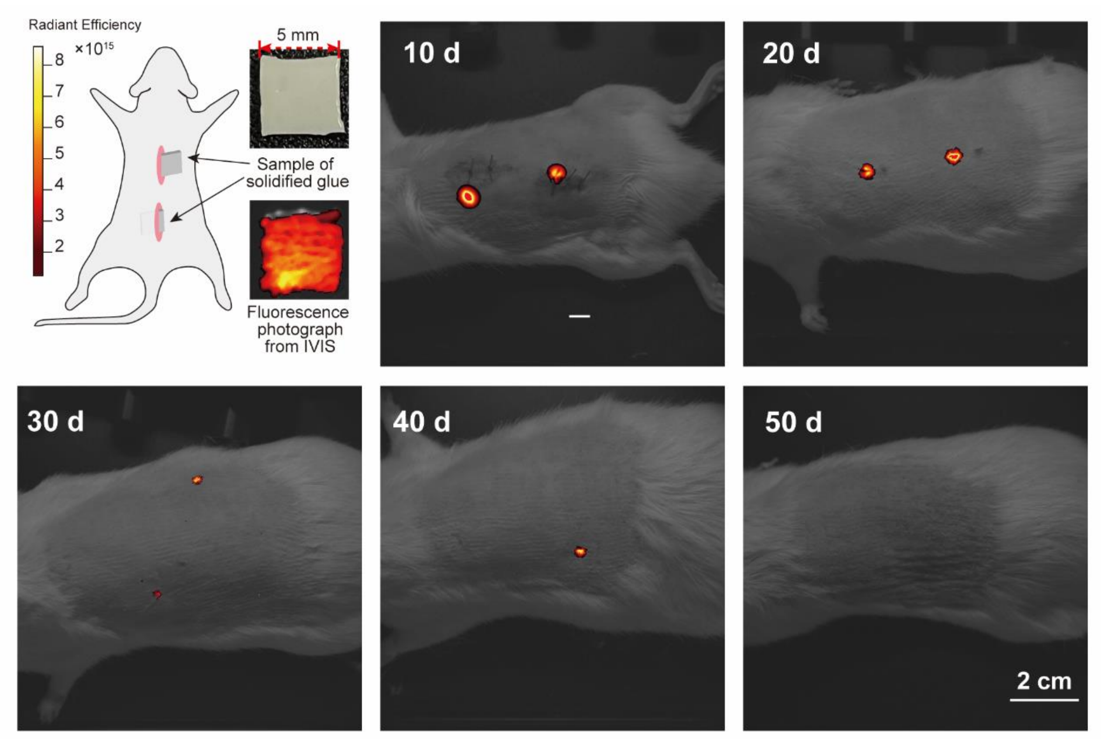

Fig. RL1 Diagram illustrating the procedure for implanting glue samples into rats, a digital and fluorescent photograph of a Cy5.5-labeled solidified glue sample, and fluorescence images of rats implanted with the glue samples during the in vivo degradation experiment.

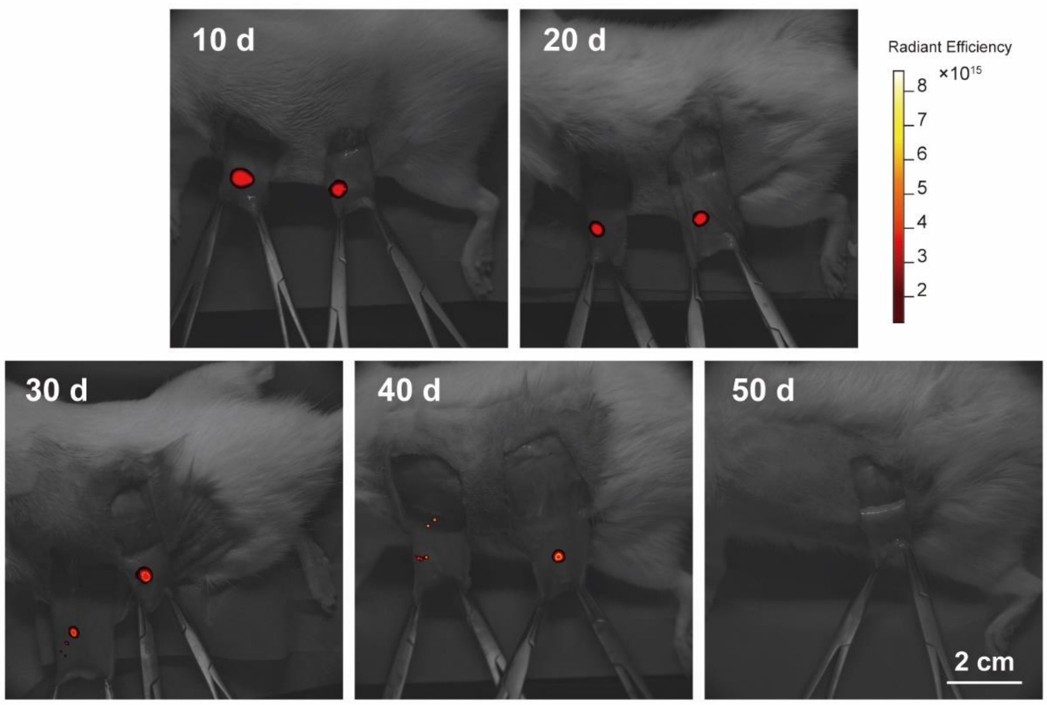

Fig. RL2 Fluorescence images of rats with open skin during the in vivo degradation experiment.

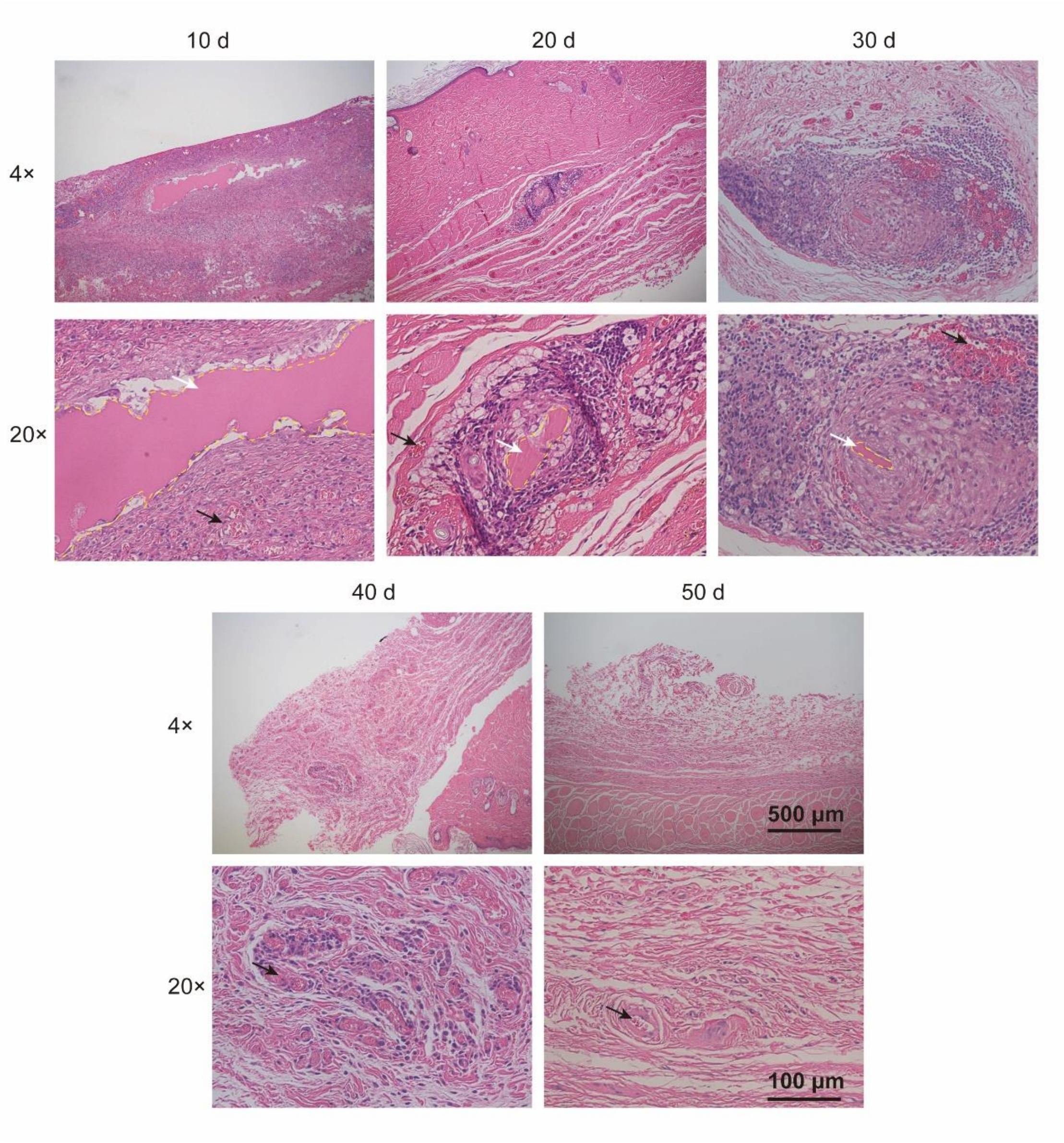

Fig. RL3 Hematoxylin and eosin (H&E) staining images of tissue around samples. The white arrow points to the glue sample and the black arrow points to the newly formed blood vessels.

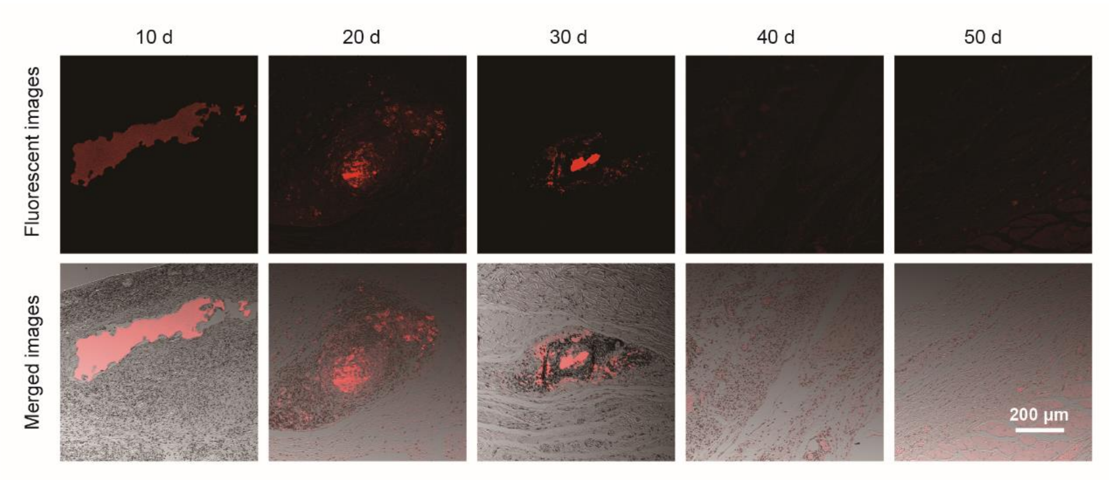

Fig. RL4 Laser scanning confocal fluorescence microscopy images of tissue around samples. The fluorescent channel is corresponding to the fluorescence of Cy5.5 (excitation, 640 nm; emission, 647LP nm).

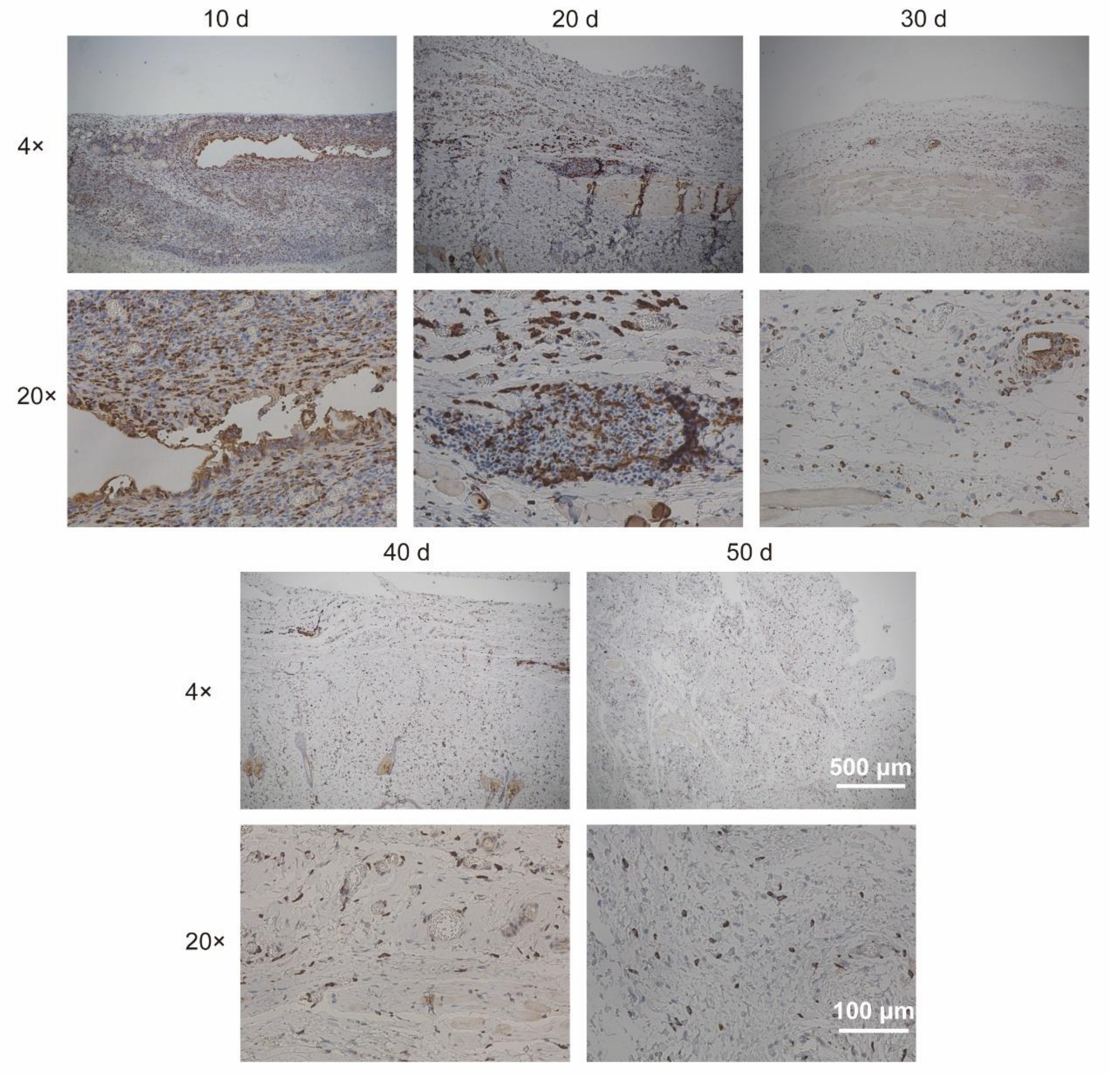

Fig. RL5 CD68 + staining images of tissue around samples under different days.

Table RL1. Serum biochemical changes in SD rat after implantation of solidified glue

Test names	Unit	Reference range	Control	10 days	20 days	30 days	40 days	50 days
ALT (Alanine aminotransferase)	U/L	6-114	72.4 ± 46.02	51.88 ± 3.21	51.57 ± 7.71	51.7 ± 13.02	50.89 ± 3.44	57.35 ± 13.88
AST (Aspartate aminotransferase)	U/L	37-205	122.87 ± 15.16	164.41 ± 30.65	126.26 ± 9.85	194.73 ± 47.12	125.16 ± 17.06	141.49 ± 49.18
TBIL (Bilirubin, total)	umol/L	2.57-36.85	15.5 ± 0.92	7.81 ± 0.95	8.43 ± 2.27	9.88 ± 4.73	9.88 ± 2	7.25 ± 0.69
DBIL (bilirubin, direct)	umol/L	2.24-16.89	8.56 ± 0.16	4.51 ± 0.71	5.64 ± 1.68	6.67 ± 2.67	3.93 ± 0.9	3.28 ± 0.3
ALB (Albumin)	g/L	29-48	34.33 ± 2.79	28.85 ± 0.69	29.2 ± 0.85	27.08 ± 6.51	33.43 ± 1.17	33.9 ± 1.36
ALP (Alkaline phosphatase)	U/L	12.04-610.97	147.97 ± 58.04	353.38 ± 41.7	227.37 ± 7.94	225.34 ± 68.9	214.85 ± 27.57	275.15 ± 34.78
γ-GT (Gamma-glutamyl transpeptidase)	U/L	0.5-5.3	3.57 ± 1.41	2.26 ± 0.53	2.6 ± 0.8	3.19 ± 1.21	1.3 ± 0.27	1.37 ± 0.3
TBA (serum bile acid)	umol/L	0-8.51	8.47 ± 3.15	7.97 ± 2.69	7.68 ± 3	7.81 ± 3.82	7.41 ± 4.5	7.19 ± 7.38
Urea	mmol/L	3.48-8.11	4.51 ± 0.46	4.28 ± 1.08	2.87 ± 0.39	3.5 ± 0.82	4.82 ± 0.4	5.88 ± 0.38
CREA(Creatinine)	umol/L	10.90-118.07	48.22 ± 7.34	26.65 ± 3.08	27.74 ± 2.61	31.78 ± 8.48	24.83 ± 2.05	23.99 ± 3.35
GLU (Glucose)	mmol/L	4.98-15.95	10.37 ± 2.39	10.76 ± 1.55	12.19 ± 2.55	14.56 ± 3.78	12.36 ± 2.01	13.79 ± 4.86
Ca^{2+}	mmol/L	2.3-3.25	2.32 ± 0.02	2.2 ± 0.04	2.36 ± 0.05	2.06 ± 0.2	2.1 ± 0.05	2.22 ± 0.08
Cl^-	mmol/L	94-116	106.74 ± 0.7	104.43 ± 1.42	96.4 ± 1.05	109.3 ± 0.18	110.19 ± 0.85	107.04 ± 3.96
Na^+	mmol/L	139-155	152.15 ± 0.45	134.05 ± 1.53	132.27 ± 1.99	153.12 ± 5.61	148.07 ± 1.78	146.6 ± 2.24
K^+	mmol/L	3.6-7.7	5.24 ± 0.17	4.6 ± 0.36	5.54 ± 0.27	5.14 ± 0.77	4.27 ± 0.89	4.55 ± 0.15

* Reference ranges were modified from Loeb et al (1999)

(Loeb, WF and Quimby, FW. 1999. The Clinical Chemistry of Laboratory Animals, 2nd ed. Philadelphia: Taylor & Francis USA.)

The authors could check out additional minor comments below.

minor comments

Please check the following for typo and errors in the manuscript.

It would be better to change the caption of figure 3 to bold like other figures.

In the Figure 4, the explanation of the captions is not matched, and caption (f) is duplicated.

In the Figure 5, the caption (b) is cut off.

There is no caption e in the Figure 6 in the description of the caption.

Responses: Thank you very much for pointing out these errors, and we have corrected them in the revised manuscript.

Reviewer #3 (Remarks to the Author):

This manuscript is transfered to Nature Comm after the authors' revision. Compared to previous version, the revised manuscript is now well improved. But, several points should be still considered and revised.

1. Please provide essential information about Fig. 6g:

- the x- and y-axis titles of the inset graph
- equipment and X-ray wavelength (Å) used in XRD study.

Responses: Thank you very much for pointing out these errors. In the revised version of the manuscript, x- and y-axis titles have been added to Fig. 6g, and the caption now includes information about the equipment used and X-ray wavelength (Å). Additionally, the experimental methods section now provides a detailed description of the testing procedure for XRD.

2. Additional information is required to verify the correspondence between Fig. S43b,

S43d and Equation 5. Please present the coefficient of determination (R2 value), and check whether the y-intercepts of regression line in the Fig. S43b and S43d match the value of ln(π/4) at Equation 5. Also, which wavenumber (cm-1) was selected as the characteristic peak from the FTIR-ATR spectra to be plotted?

Responses: Thank you very much for your suggestion. We have added the intercept and R^2 of the fitted equation to Fig. S49b and S49d. The wavenumber selected for fitting was 3278 cm^{-1}. For the uncured adhesive, the intercept was 0.024 and R^2 was 0.99, while for the cured adhesive, the intercept was 0.014 and R^2 was 0.97. It is clear that neither of the intercepts obtained is equal to ln(4/π) (~0.24). This was due to our oversight in not giving the applicable conditions of the equation. We have made corresponding revisions in the new article. The formula (2) we used is simplified by eliminating all terms in the series beyond the first of formula (1). This simplification only holds when $A_t/A_\infty \geq 0.5$. As shown in Fig. RL6, the first term of the formula is significantly different from the 50th term when t is small, indicating that formula (2) is not applicable for small t. Therefore, when we use formula (3) for linear fitting of diffusion coefficient, the intercept of the fitting curve is not equal to ln(4/π).

$$\frac{A_t}{A_\infty} = 1 - \frac{8\gamma}{\pi[1-\exp(-2\gamma l)]} \sum_{n=0}^{\infty} \left[\frac{\exp\left(\frac{-D(2n+1)^2\pi^2 t}{4l^2}\right)\left(\frac{(2n+1)\pi}{2l}\exp(-2\gamma l)+(-1)^n(2\gamma)\right)}{(2n+1)\left(\frac{((2n+1)\pi)^2}{4l^2}+4\gamma^2\right)} \right] \tag{1}$$

$$\frac{A_t}{A_\infty} = 1 - \frac{8\gamma}{\pi[1-\exp(-2\gamma l)]} \times \left[\frac{\exp\left(\frac{-D\pi^2 t}{4l^2}\right)\left(\frac{\pi}{2l}\exp(-2\gamma l)+(2\gamma)\right)}{\left(\frac{\pi^2}{4l^2}+4\gamma^2\right)} \right] \tag{2}$$

$$\ln\left(1-\frac{At}{A_\infty}\right) = \ln\left(\frac{4}{\pi}\right) - \frac{D\pi^2}{4l^2}t \tag{3}$$

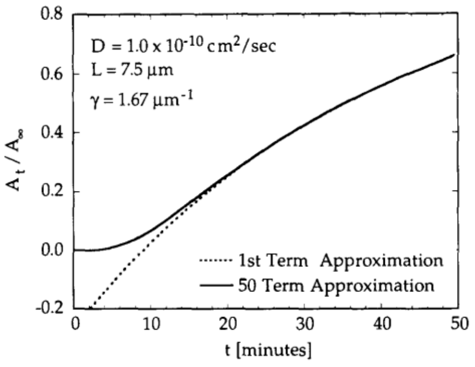

Fig. RL6 Comparison of 50 term approximation and first term approximation of formula (1) (Polymer, 1993, 34(6): 1146-1153)

3. From Fig. 3j, the CD spectrum of solidified glue around 215 nm, corresponding to the β-sheet, appears to be flat rather than has peak or valley. Can it be said that the β-sheet has significantly increased by solidification with this data, or is it possible to provide complementary data to support the claim?

Responses: Thank you for your comments. CD spectrum is a very sensitive method for reflecting changes in protein secondary structure. Although the CD characteristic peaks of the solidified glue appear to become flatter after curing, there are significant changes in the secondary structure. During the measurement, we ensured that both the control and experimental groups had the same protein concentration, which was 0.1 mg/mL. In order to further quantitatively analyze the changes in secondary structure by CD spectrum, we used an online tool called BeStSel developed by Micsonai et al. (https://bestsel.elte.hu/index.php) to estimate the specific content of the secondary structure, and the results were shown in Table RL2. After solidifying, the content of β-sheet increased by 35% from 0.4%, which is also supported by the deconvolution of the amide I FTIR spectra and ThT and Congo red staining results (Fig. 3i and S10b).

Table RL2 | Estimated secondary structure content by BeStSel

Second structure	Solidified glue (%)	Native BSA solution (%)
α-helix	2.6	64.7
β-sheet	35.3	0.4
Turn	13.2	10.7
Other	48.9	24.2

Reviewer #4 (Remarks to the Author):

1. The adhesion to a surface is the cumulative effect of adhesive and cohesive forces. Maximizing one works against the other and the trick is to obtain the optimum of each component. The destabilization of the proteins with a disulfide disruptor followed by the addition of trichloroethanol (TCE) to dissolve the protein converts that into a flowable liquid, which is able to make good contact with the surface. Removal of the organic TCE by immersion in water converts the protein back to a solid hence increasing adhesion. Although the result is interesting, there is nothing special in these results. It is what is expected from an understanding of interfacial science. The finding described here is not special as these results will not hold beyond being a publication.

Responses: Thank you for your comments. Underwater stable adhesion has always been an important area in interfacial science. There are currently two challenges that must be overcome in order to achieve underwater adhesion. One is how to break the material's hydration layer, and the other is how to resist water swelling and hydrolysis. This has caused many adhesives that have good adhesive properties in dry conditions to almost lose their effectiveness underwater. Therefore, the development of underwater

adhesives is therefore focused on overcoming these two points. In this study, an unfolding-aggregating strategy was established to successfully overcome these two challenges, thereby making common proteins into underwater adhesives. We believe that our results are not "nothing special", and at least three highlights make this system stand out:

1) **A new design principle was proposed to convert dozens of common commercial water-soluble proteins into underwater glue without any additional chemical modification.** In the past, some specific proteins were often required for developing underwater protein adhesives, which were very costly and difficult to obtain. However, the strategy proposed in this article can transfer common commercial water-soluble proteins into underwater glue as long as the proportion of high amyloid-propensity (P_{HAP}) segments in the protein is greater than 16.77%. It is always a challenging task to transfer water-soluble proteins into underwater gule as well as maintain good underwater stability to prevent dissolution or swelling. However, we have overcome this problem through the universal strategy of unfolding and aggregation. The revelation of this universal law has not been reported in the literature. In addition, ordinary commercial proteins can be transfer into underwater glue using this method, which solves the shortcomings of existing protein underwater glue, such as limited and high cost raw materials.

2) **A new strategy has been developed to control the protein conformation evolution ("unfolding-aggregating") and integrate the amyloid-like structure into protein-based glue.** The amyloid-like structure greatly enhances the stability and mechanical strength of protein materials. Previous underwater adhesives often used gene fusion expression (Nature Nanotechnology, 2014, 9(10): 858-866) or chemical modification methods (Advanced Science, 2021, 8(16): 2004786) to merge

the amyloid-like structure with the polyphenolic groups inspired by mussels and oysters to improve the strength of underwater adhesives. However, these methods are complex and difficult to overcome the disadvantage of polyphenolic groups' alkaline intolerance. This study shows that by simply controlling the folding and aggregation of proteins, the amyloid-like structure can be introduced into protein underwater glue to obtain high-strength and stable underwater adhesives.

3) **The mechanism behind amyloid-mediated bioadhesion is partly deciphered that may include conformation-directed adhesion enhancement.** Research on amyloid-mediated bioadhesion has been a hotspot, but the mechanisms remain unclear. On the one hand, marine organisms such as barnacles use protein amyloid assembly during adhesion, and the study of their adhesion mechanism can provide new ideas for marine antifouling. On the other hand, decoding the secrets of amyloid assembly can help develop stronger and more stable underwater adhesives. In this study, the process of adhesion caused by the amyloid-like aggregation of common proteins was characterized, and the concept of conformation-directed adhesion enhancement was proposed. In detail, flexible hydrophobic chains break through the hydration layer, protein-rich groups provide functional sites, a large number of β-sheet microcrystalline regions provide strength, and amorphous regions provide toughness. These abundant multi-scale conformations of the protein ultimately combine to form a strong and stable protein underwater glue.

2. One cannot use a fluorinated solvent to apply a glue in a biomedical application, and probably not for commodity applications also due to the environmental footprint of fluorinated solvents.

Responses: Thank you for your comments. In this paper, we mainly

emphasize a new scientific finding, namely, the transformation of common commercial water-soluble proteins into underwater glue through unfolding-aggregating. To achieve stable unfolded protein, reducing agents for disulfide bonds and unfolding stabilizers need to be added. Trifluoroethanol is just one of the many stabilizers we chose. In addition to trifluoroethanol, we also used stabilizers such as guanidine hydrochloride, urea, and arginine. Although the environmental hazards of trifluoroethanol cannot be ignored, it is still widely used in industrial applications, such as the preparation of many pesticides and drugs, and even the popular electrospinning technology requires a large amount of as solvents. In fact, the global consumption of trifluoroethanol has been increasing, with a market size of $580 million in 2021, and it may reach $911 billion by 2029. (https://www.maximizemarketresearch.com/market-report/global-trifluoroethanol-market/78154/), which indicates that its industrial applications are to some extent allowed. On the other hand, in this article, trifluoroethanol has been shown to have the best stabilizing effect on unfolding proteins and the best adhesive properties when used to prepare adhesives. Therefore, we believe that TFE-stabilized glue can be used in emergency situations, such as leaks in inflatables while at sea (Fig. 5c). Additionally, the strength of adhesives prepared using guanidine hydrochloride can also reach above 2.5 MPa, fully satisfying scenarios that require a large amount of adhesive use.

For the adhesive used in biomedical applications, we utilize the urea/cysteine system with good biosafety, as both urea and cysteine are substances found naturally in living organisms. We also carefully explained the principle of using urea as a stabilizer, which is included in the Supporting Information. Unfolded protein underwater glue prepared using this system exhibit excellent biological safety. We extensively and thoroughly characterized the in vivo and in vitro toxicity and degradability of this system (Fig. S33-S44), and the results showed that it is not only non-toxic but also has good degradability

(completely degraded within 50 days), making it suitable for use in the biomedical field.

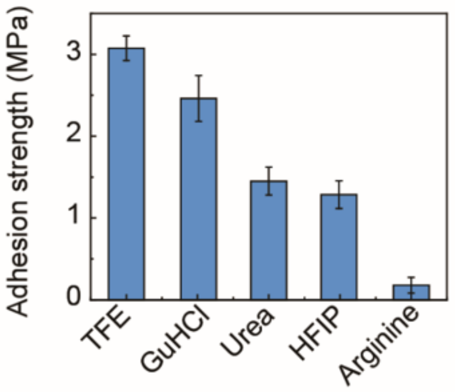

Fig. RL7 The adhesion strength of unfolded BSA prepared with different stabilizers.

3. This is the challenge in designing glues for biomedical applications. Although several reports have demonstrated design of adhesives that do not use any solvent for application or the use of non-toxic components, they are not clinically advanced to displace current fibrin glues. The manuscript has not referenced these prior studies.

Responses: Thank you for your comments. Currently, there are very few adhesives that can replace fibrin glues, because as David Mooney stated, most newly developed tissue adhesives suffer from weak adhesive strength and poor mechanical properties (Chem. Rev. 2021, 121, 18, 11336–11384). In addition, they also believe that "An ideal tissue adhesive should possess a number of characteristics, including (1) biological compatibility and nontoxicity, (2) chemistry suitable to create robust tissue adhesion, (3) mechanical similarity to the underlying tissue, (4) mechanical capacity to withstand repeated dynamic forces imposed by the tissue, (5) acceptable swelling profile to minimize tissue compression, and (6) biodegradability at a rate compatible with tissue healing." In our study, we have demonstrated through a large number of experiments that the glue has good biological safety (Fig.

S33-38), excellent adhesive strength (Fig. 4e), adjustable modulus to adapt to different tissues (Fig. S30), can withstand repeated dynamic forces (Fig. S26), good resistance to swelling (Fig. 3h), and the ability to completely degrade within 50 days (Figs. S39-44). Therefore, it is evident that this protein glue indeed has the potential to be used as a tissue adhesive.

In addition, we have cited a review of tissue adhesives in the section on wound closure in rats, adding the following content: "A number of clinically used tissue adhesives, such as fibrin, gelatin resorcinol, and cyanoacrylates, are considered inadequate due to toxicity or mechanical properties.[55]"

4. There are also several other reasons why this manuscript is not suitable for publication, including the authors' propensity to use superlative and unsupported language that makes the manuscript sound more like an advertisement than a scientific report.

Responses: Thank you for your comments. Admittedly, some descriptions in our manuscript may have overly emphasized superiority, so we have revised some of the conclusions**.** Through carefully revising the paper (adding a large amount of theoretical and empirical evidence), the scientificity of the paper has been greatly improved. Our work is based on careful investigation from theoretical and experimental respects. The scientific writing in the revised manuscript can be further reflected by that: all the conclusions of this study are drawn by a number of theoretical calculations and experimental verifications. In the theoretical calculation, a "unfolding-aggregating" controlled strategy is computed through molecular dynamics simulation, a Rosetta energy calculation is used to determine which protein can be converted into underwater glue, and a Finite Element analysis is used to evaluate the solidification process. The experimental verification consists of synchrotron small-angle X-ray scattering to investigate the glue preparation

mechanism, high resolution ultrathin-section TEM to examine the internal sea-island structure, TOF-SIMS to determine the surface adhesion mechanism, and many in vitro and in vivo experiments to prove the biosafety and potential biological application of the materials.

We have revised some of the conclusions, such as changing the conclusion regarding adhesive strength to the following sentence: "Moreover, unfolded BSA glue has a significantly higher adhesion strength than most protein-based adhesives and even compares to synthetic adhesives (Fig. 4k)."

We have also deleted some sentences:

"The excellent stability and robustness of unfolded BSA glue is superior to most underwater adhesives."

“The powerful underwater adhesion (for glass) allowed the glue to easily lift a weight being 2.5×10^{6} times higher than the adhesive mass, largely exceeding the strength of some typical biological creatures, e.g., ants (the force produced by the mandibles of the ant approximately reaching 500 times its body weight[42])”

5. Then there are several (too many to keep track) of comparisons that are not fair or are inaccurate.

Responses: Thank you for your comments. There are three main comparisons in this study. The first is comparing the alkali-resistance of our glue to the natural adhesive of mussels, the second is comparing the adhesion strength of our glue to commercial glue, and the third is comparing the adhesion strength of our glue to previously-reported underwater glues. The first two comparisons are based on experimental data under the same test conditions, using the lap shear adhesion test, which is conducted entirely underwater (see Lap Shear Adhesion section on page 2 of the Supporting Information for more details). The comparison with previously-reported

underwater glues are also been checked carefully, and we can sure that all of the reference data were measured by lap shear adhesion test.

6. The experiments are not fully described to be repeatable. For example, the authors claim that these are underwater adhesives. (In underwater adhesives, the substrate preparation, glue application, and curing have to be done underwater). However, the experimental section does not provide sufficient information of how these experiments were done. In fact, the lap shear experimental section says that 10 L of glue was poured onto 1 cm x 1 cm glass slides. It is quite obvious that something is inaccurate in this description. There is only some video and images of application of the glue to a surface. This is not a substitute for detailed experimental protocols. Similarly, there are no details of the tensile experiments.

Responses: Thank you for pointing out this error. In the experimental section, we mistakenly wrote 10 μL as 10 L. This mistake has been corrected this in the revised Supporting Information. To improve the reproducibility of the experiment, we have carefully modified the protocol.

Substrate preparation: All the substrates to be tested, including steel, aluminum, titanium alloy, ceramics, glass, nylon 66, PVC, PMMA, PP, bone, and pigskin, were cut into 1 cm ×5 cm strips and a small hole is opened at one end of the strip for fixation during tensile testing (Fig. RL8 a-b). No further treatment was carried out on any of the substrates.

Underwater adhesion experiment: Firstly, a glass crystallizing dish with a diameter of 180 mm was filled with two-thirds of the total volume of tap water. The substrate was placed flat in the bottom of the container and 10 μL of protein glue was dropped on the end of the substrate away from the hole using a pipette. Another piece of substrate was quickly covered over it, with an overlap area of 1 cm × 1 cm. The two substrates were clamped to prevent slipping before the glue solidified. After 24 hours, the two substrates will be

firmly bonded and the clamp was removed.

Lap Shear Adhesion: The bonded substrate was transferred into a custom-made transparent box filled with water (Fig. RL8c) to ensure that the entire test is carried out underwater. The adhesion strength was measured by a universal material machine with a 100 N load cell (UTM2103, Shenzhen Suns Technology STOCK Co., Ltd.). During the measuring, the holders and samples were immersed in water. The motorized part was moved along the vertical (shear adhesion test) axis with a retraction rate of 1 mm/min until the adhesive interface separated. The adhesion strength of the glue was determined by dividing the maximum force by the overlapping area. Each condition was tested with at least 3 samples.

Tensile tests: The sample for tensile tests were prepared by pouring glue into a container filled with water and waiting for 4 hours until the glue completely solidified. The solidified glue at the bottom of the container was peeled off and cut into dumbbell-shaped blocks measuring 4 cm in length, 1 cm in width, and 0.1cm in thickness. The two ends of the sample were then fixed onto the fixture of a universal material testing machine with a 100 N load cell. (UTM2103, Shenzhen Suns Technology STOCK Co., Ltd.). The stress–elongation curve was recorded at 10 mm/min at room temperature. The tensile strength was obtained from the failure point, and the elastic modulus was determined by the average slope over 0–20% of elongation from the stress–elongation curve.

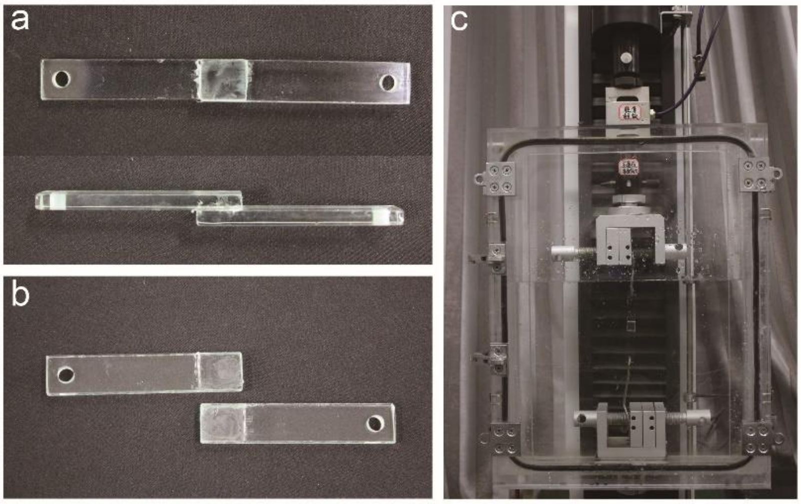

Fig. RL 8 Bonded glass slides (a and b) and adhesion strength testing equipment (c). Overlapping area is 1 cm^2.

7. The mouse wound closure studies show deposition of the glue onto a dry skin wound. It is not surprising that a protein glue would stick to a dry skin with a narrow wound. What is not discussed is that the wound now has trichloroethanol in it, which would be toxic to the tissues. Additionally, the use of the adhesives in the wound experiments were done using urea as the 'stabilizer'. Was urea dissolved in water to be added to the protein?

Responses: Thank you for your comments. According to the observations during the animal experiments, the wounds on the skin are not dry; cutting the skin of rats will result in a wet environment with some blood flowing out (Fig. RL9). Therefore, we believe that wound closure experiments are not "either not necessary or are distracting to the main message of the work," but instead provide strong evidence for the adhesive performance and resultant applications.

The TFE-stabilized glue is highly adhesive and suitable for some demanding situations, such as dealing with inflatable objects at sea. However, it is not suitable for biomedical applications. For biomedical experiments, our urea-stabilized adhesive is the safer choice due to its tissue compatibility. The

preparation method for this glue is explained in the Supporting Information, specifically, by first preparing a 50% aqueous solution of urea and then adding protein.

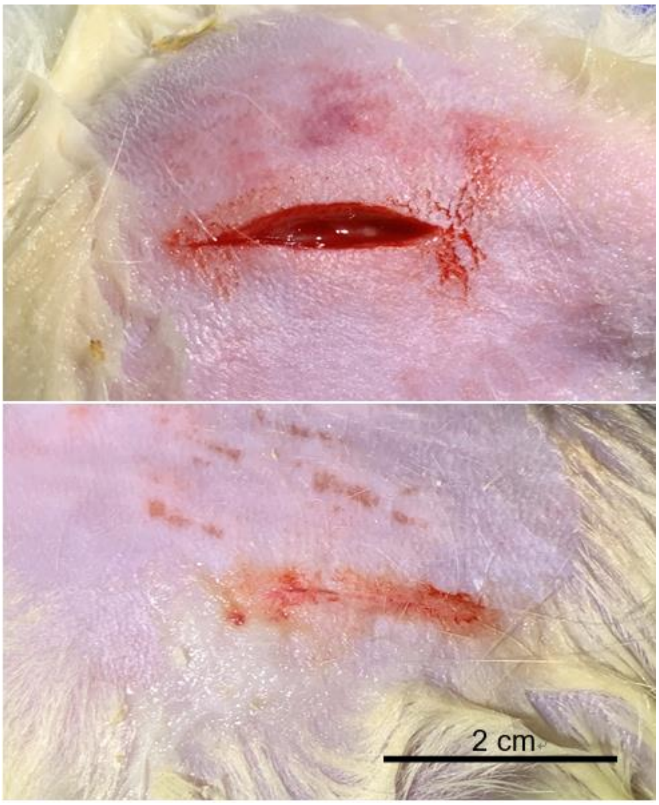

Fig. RL9 Photograph of the untreated skin wound (upper) and unfolded BSA glue-closed skin wound (lower).

8. The use of urea and the resultant lap shear test results were not provided in the prior sections. The mechanism of action of urea and TCE cannot be the same. These were not described in the manuscript. There are several experiments such as the above that are either not necessary or are distracting to the main message of the work.

Responses: Thank you for your comments. The resultant lap shear test results were listed in Fig. RL7.

The mechanism of action of urea is similar to TFE, which can also stabilize

the unfolding state of protein. To determine whether urea has a similar effect as TFE, we characterized the glue prepared using urea. NPM characterization of the urea-treated BSA showed a strong fluorescence enhancement, indicating that disulfide bonds were reduced in the system (Fig. RL10a). The system also showed a decrease in the intramolecular packing density of proteins, as determined by synchrotron small-angle X-ray scattering (SAXS), indicating the unfolding of protein in the urea contained system (Fig. RL10b). The corresponding Kratky plots of urea-treated BSA showed a plateau instead of converging around zero, suggesting that the BSA in urea-treated glue was noticeably unfolded. Protein unfolding was further proven by viscosity analysis (Fig. RL10c). The change in viscosity with the concentration of BSA in a urea solution (50% in volume) indicated a better fitting with the Einstein equation (R^2 = 0.97) than with the Huggins equation (R^2 = 0.95) (Fig. RL10d). This suggested that the unfolded BSA adopted a chain-like structure instead of its native spherical structure. Following stabilizer-controlled unfolding, the solidification of unfolded proteins to form underwater glue could be triggered by the urea removal in water. When the concentration of urea drops below 4%, the unfolded protein can aggregate (Fig. RL10e). Upon injection into water for 5 min, the glue solidification occurred sufficiently to afford a written pattern tightly adhered on the glass surface that withstand strong rinsing with tap water from the faucet (Fig. RL10f). With Congo red staining, a visible red color was present on the glue due to the specific binding of Congo red with the β-sheet structure inside the adhesive (Fig. RL10g). Further thioflavin-T (ThT) staining of the solidified glue resulted in significant enhancement of the ThT fluorescence intensity, indicating the possible presence of amyloid-like structures in the protein glue. In addition to the staining assay, spectral analysis including far-ultraviolet circular dichroism (far-UV CD) and Fourier-transform infrared spectroscopy (FTIR), provided direct evidence of the conformational change during protein unfolding and aggregation. According to CD spectra, the solidified glue exhibited distinctive characteristic peaks of β-

sheet (216 nm) (Fig. RL10h). Deconvolution of the amide I FTIR spectra during the solidification further demonstrated that the characteristic β-sheet peaks (1630 and 1691 cm^{-1}) increased significantly compared to those of native BSA (Fig. RL10i). All of the above results demonstrate that urea, similar to TFE, can maintain the protein's folded and unfolded states, and after adding this system to water, urea can also diffuse into water, leading to protein aggregation. It is well known that breaking disulfide bonds exposes hydrophobic patches in proteins, and the effective way to stabilize the protein's folded state is to prevent the hydrophobic domain from coming together and aggregating. Urea, guanidine hydrochloride, and TFE can all bind well with the hydrophobic domain in proteins (The Journal of Physical Chemistry B, 2002, 106(6): 1491-1500.), thereby preventing protein aggregation. It is based on this principle that we can obtain stable unfolded proteins.

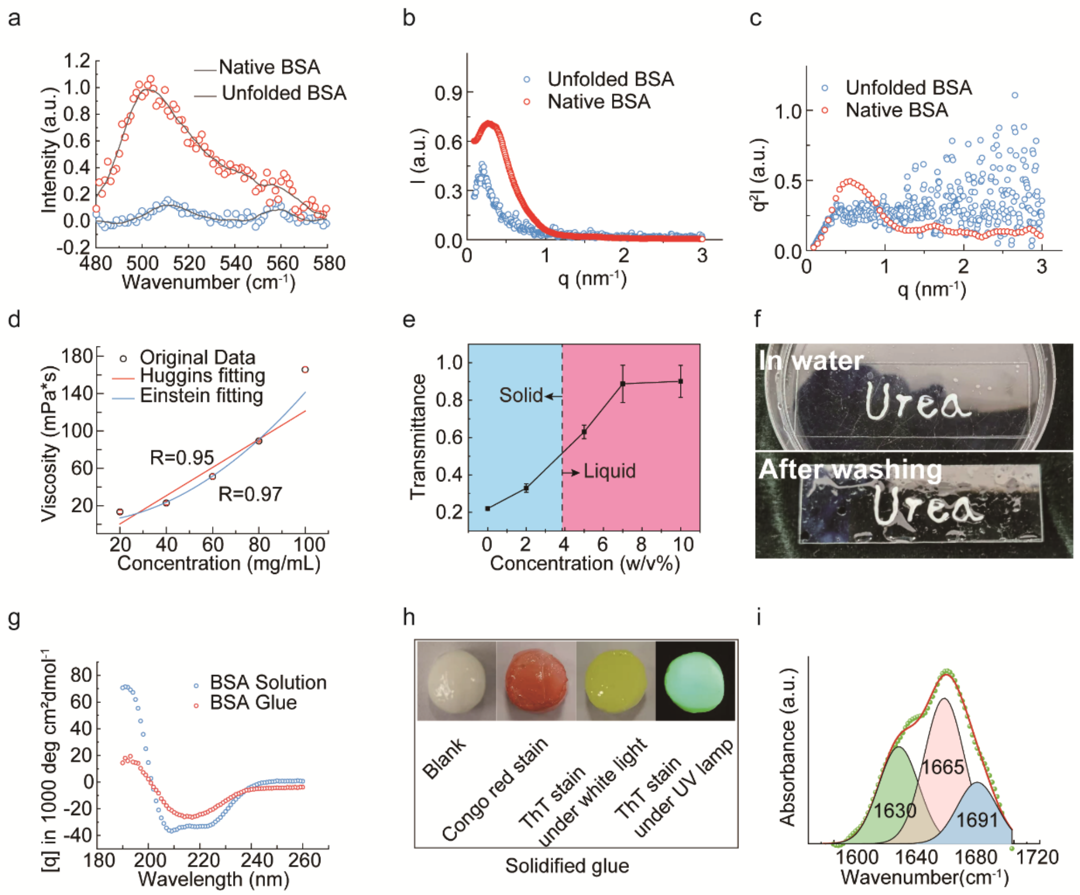

Fig. RL10 The unfolding and aggregation of urea/cysteine system-treated

BSA. (a) Raman spectra of native BSA and unfolded BSA. The peak between 480 ~ 580 cm-1 belongs to the S-S bond of the protein. Scattering plots (I ~ q) (b) and corresponding Kratky plots ($I \cdot q^2 \sim q$) (c) of native and unfolded BSA. I, scattering intensity; q, scattering vector. (d) Concentration dependence of the viscosity change of unfolded BSA solution. It shows a better fitting with the Einstein equation (R^2 = 0.97) than with the Huggins equation (R^2 = 0.95), indicating that the BSA in this system is unfolded. (e) Light transmittance (wavelength = 600 nm) of unfolded BSA solution with urea added at different concentration. (f) The word “Urea" written on an underwater glass slide by using the unfolded BSA glue and it can withstand strong rinsing with tap water from the faucet. (g) CD spectra of the unfolded BSA and its aggregation triggered by water. (h) Photograph of ThT and Congo red staining of the solidified unfolded BSA glue. (i) The deconvolution of amide I from 1600 to 1720 cm^{-1} for solidified BSA.

9. It is understandable that MD simulations can predict interactions between proteins and surface. But is not easy to understand how MD simulations can translate those interactions into lap shear strength data.

Responses: Thank you for pointing out this problem. Lap shear strength can be calculated according to molecular dynamics simulations. In this paper, a steered molecular dynamic simulation (SMD) was conducted to analyze the tensile stress. In detail, one silica slide was fixed, and another silica slide was dragged by a constant force of 1000 kJ/mol/nm in a direction parallel to the silica slide. The calculation of lap shear strength is based on the method by Amir et al. (arXiv preprint arXiv:2011.11218, 2020), and its calculation process is as follows:

The shear adhesion strength (τ) can be calculated by Equation 6

$$\tau = \frac{F_{shearing}}{substrate\ contact\ area\ (A)} \quad (6)$$

where $F_{shearing}$ is the tensile breaking force and the *substrate contact area (A)* is the area of the silica slide. The method of calculating lap shear strength using MD simulation has been added to the Supporting Information.

Reviewers' Comments:

Reviewer #1:
Remarks to the Author:
[Note from the Editor: Reviewer #4 was unfortunately not able to look over the revision again and therefore reviewer #1 was asked to assess the response to reviewer #4.]

The authors seem to have made an effort through further discussion and testing to address the raised issues (particularly biocompatibility). Analysis of their adhesive materials is considered meaningful. However, for the medical application of the developed adhesive material, continuous additional research is needed for commercialization. Although it is judged that the authors have proven it to somewhat to the preclinical experiment, it is recommended to tone down the entire paper, including the descriptions of the potential utility and the need for additional research, for application to their adhesive material. If these are reflected, I agree that the paper will be accepted for publication.

Reviewer #3:
Remarks to the Author:
The revised version is now acceptable as is in Nature Communications.

Reviewer #1 (Remarks to the Author):

The authors seem to have made an effort through further discussion and testing to address the raised issues (particularly biocompatibility). Analysis of their adhesive materials is considered meaningful. However, for the medical application of the developed adhesive material, continuous additional research is needed for commercialization. Although it is judged that the authors have proven it to somewhat to the preclinical experiment, it is recommended to tone down the entire paper, including the descriptions of the potential utility and the need for additional research, for application to their adhesive material. If these are reflected, I agree that the paper will be accepted for publication.

Responses: Thank you so much for your constructive suggestion. We agree with the reviewer's comment, and have toned down the descriptions of the potential medical applications of the bioglue, while also including additional statements emphasizing the need for further research to establish its biocompatibility.

The revised content is outlined below:

Deleted sentence:

1.“Differently, this protein-based glue has potential for clinical application because of its good biosafety, favorable degradability, robust adhesion strength…”

Changed sentences:

1.“According to cytotoxicity tests using neuronal cells (HT22) (Supplementary Fig. 35) and various visceral cells (HepG2, HUH-7, HL-7702, and HEK293) (Supplementary Fig. 36-38), hemolysis (Supplementary Fig. 39), acute toxicity (Supplementary Tab. 2), and pathology examinations of organs (Supplementary Fig. 40), the urea-stabilized unfolding BSA adhesive displays excellent biocompatibility.” → *“According to cytotoxicity tests using neuronal cells (HT22) (Supplementary Fig. 35) and various visceral cells (HepG2, HUH-7, HL-7702, and HEK293) (Supplementary Fig. 36-38), hemolysis (Supplementary Fig. 39), acute toxicity (Supplementary Tab. 2), and pathology examinations of organs (Supplementary Fig. 40), the urea-stabilized unfolding BSA adhesive does not exhibit obvious biotoxicity.”*

2.“Also, serum biochemical analyses were conducted during the entire implantation period (Supplementary Tab. 3) of rats, and the results indicated that the various indicators in the blood were within the normal range, which also indicated that the glue was biologically safe.” →“Also, serum biochemical analyses were conducted during the entire implantation period (Supplementary Tab. 3) of rats, and the results indicated that the various indicators in the blood were within the normal range, which indicates that the glue has almost no influence on the function of organs and tissues of rats, as well as the metabolic state.”

***Added* sentence:**

1.“Although these cell and animal experiments have provided preliminary evidence of the biosafety of glue, further research is still needed to prove its usefulness in clinical applications.”
